# Dynamic phenotypic heterogeneity and the evolution of multiple RNA subtypes in hepatocellular carcinoma: the PLANET study

**DOI:** 10.1093/nsr/nwab192

**Published:** 2021-10-29

**Authors:** Weiwei Zhai, Hannah Lai, Neslihan Arife Kaya, Jianbin Chen, Hechuan Yang, Bingxin Lu, Jia Qi Lim, Siming Ma, Sin Chi Chew, Khi Pin Chua, Jacob Josiah Santiago Alvarez, Pauline Jieqi Chen, Mei Mei Chang, Lingyan Wu, Brian K P Goh, Alexander Yaw-Fui Chung, Chung Yip Chan, Peng Chung Cheow, Ser Yee Lee, Juinn Huar Kam, Alfred Wei-Chieh Kow, Iyer Shridhar Ganpathi, Rawisak Chanwat, Jidapa Thammasiri, Boon Koon Yoong, Diana Bee-Lan Ong, Vanessa H de Villa, Rouchelle D Dela Cruz, Tracy Jiezhen Loh, Wei Keat Wan, Zeng Zeng, Anders Jacobsen Skanderup, Yin Huei Pang, Krishnakumar Madhavan, Tony Kiat-Hon Lim, Glenn Bonney, Wei Qiang Leow, Valerie Chew, Yock Young Dan, Wai Leong Tam, Han Chong Toh, Roger Sik-Yin Foo, Pierce Kah-Hoe Chow

**Affiliations:** Genome Institute of Singapore, Agency for Science, Technology and Research, Singapore 138672, Singapore; Key Laboratory of Zoological Systematics and Evolution, Institute of Zoology, Chinese Academy of Sciences, Beijing 100101, China; Center for Excellence in Animal Evolution and Genetics, Chinese Academy of Sciences, Kunming 650223, China; Genome Institute of Singapore, Agency for Science, Technology and Research, Singapore 138672, Singapore; Genome Institute of Singapore, Agency for Science, Technology and Research, Singapore 138672, Singapore; School of Biological Sciences, Nanyang Technological University, Singapore 637551, Singapore; Genome Institute of Singapore, Agency for Science, Technology and Research, Singapore 138672, Singapore; Genome Institute of Singapore, Agency for Science, Technology and Research, Singapore 138672, Singapore; Key Laboratory of Zoological Systematics and Evolution, Institute of Zoology, Chinese Academy of Sciences, Beijing 100101, China; Genome Institute of Singapore, Agency for Science, Technology and Research, Singapore 138672, Singapore; Cell and Developmental Biology, Division of Biosciences, Faculty of Life Sciences, Bloomsbury, London WC1E 6AP, UK; Genome Institute of Singapore, Agency for Science, Technology and Research, Singapore 138672, Singapore; Genome Institute of Singapore, Agency for Science, Technology and Research, Singapore 138672, Singapore; Division of Surgery and Surgical Oncology, National Cancer Centre, Singapore 169610, Singapore; Genome Institute of Singapore, Agency for Science, Technology and Research, Singapore 138672, Singapore; Genome Institute of Singapore, Agency for Science, Technology and Research, Singapore 138672, Singapore; Genome Institute of Singapore, Agency for Science, Technology and Research, Singapore 138672, Singapore; Genome Institute of Singapore, Agency for Science, Technology and Research, Singapore 138672, Singapore; Division of Surgery and Surgical Oncology, National Cancer Centre, Singapore 169610, Singapore; Department of Hepato-Pancreato-Biliary and Transplant Surgery, Singapore General Hospital, Singapore 169608, Singapore; Department of Hepato-Pancreato-Biliary and Transplant Surgery, Singapore General Hospital, Singapore 169608, Singapore; Department of Hepato-Pancreato-Biliary and Transplant Surgery, Singapore General Hospital, Singapore 169608, Singapore; Department of Hepato-Pancreato-Biliary and Transplant Surgery, Singapore General Hospital, Singapore 169608, Singapore; Department of Hepato-Pancreato-Biliary and Transplant Surgery, Singapore General Hospital, Singapore 169608, Singapore; Department of Hepato-Pancreato-Biliary and Transplant Surgery, Singapore General Hospital, Singapore 169608, Singapore; Division of Hepatobiliary and Pancreatic Surgery, Department of Surgery, University Surgical Cluster, National University Health System, Singapore 119228, Singapore; Division of Hepatobiliary and Pancreatic Surgery, Department of Surgery, University Surgical Cluster, National University Health System, Singapore 119228, Singapore; Hepato-Pancreato-Biliary Surgery Unit, Department of Surgery, National Cancer Institute, Bangkok 10310, Thailand; Division of Pathology, National Cancer Institute, Bangkok 10400, Thailand; Department of Surgery, Faculty of Medicine, University of Malaya, Kuala Lumpur 59100, Malaysia; Department of Surgery, Faculty of Medicine, University of Malaya, Kuala Lumpur 59100, Malaysia; Department of Surgery and Center for Liver Disease Management and Transplantation, The Medical City, Pasig City, Metro Manila, Philippines; Department of Laboratories, The Medical City, Pasig City, Metro Manila, Philippines; Department of Pathology, Singapore General Hospital, Singapore 169608, Singapore; Department of Pathology, Singapore General Hospital, Singapore 169608, Singapore; Institute for Infocomm Research, A*STAR, Singapore 138632, Singapore; Genome Institute of Singapore, Agency for Science, Technology and Research, Singapore 138672, Singapore; Department of Pathology, National University Health System, Singapore 119228, Singapore; Division of Hepatobiliary and Pancreatic Surgery, Department of Surgery, University Surgical Cluster, National University Health System, Singapore 119228, Singapore; Department of Pathology, Singapore General Hospital, Singapore 169608, Singapore; Division of Hepatobiliary and Pancreatic Surgery, Department of Surgery, University Surgical Cluster, National University Health System, Singapore 119228, Singapore; Department of Pathology, Singapore General Hospital, Singapore 169608, Singapore; Translational Immunology Institute (TII), SingHealth Duke-NUS Academic Medical Centre, Singapore 168753, Singapore; Division of Gastroenterology and Hepatology, University Medicine Cluster, National University Hospital, Singapore 119228, Singapore; Genome Institute of Singapore, Agency for Science, Technology and Research, Singapore 138672, Singapore; School of Biological Sciences, Nanyang Technological University, Singapore 637551, Singapore; Department of Biochemistry, Yong Loo Lin School of Medicine, National University of Singapore, Singapore 117597, Singapore; Cancer Science Institute of Singapore, National University of Singapore, Singapore 117599, Singapore; Division of Medical Oncology, National Cancer Center Singapore, Singapore 169610, Singapore; Genome Institute of Singapore, Agency for Science, Technology and Research, Singapore 138672, Singapore; Cardiovascular Research Institute, National University of Singapore, National University Healthcare System, Singapore 119228, Singapore; Genome Institute of Singapore, Agency for Science, Technology and Research, Singapore 138672, Singapore; Division of Surgery and Surgical Oncology, National Cancer Centre, Singapore 169610, Singapore; Department of Hepato-Pancreato-Biliary and Transplant Surgery, Singapore General Hospital, Singapore 169608, Singapore; Singhealth-Duke-NUS Academic Surgery Program, Duke-NUS Graduate Medical School, Singapore 169857, Singapore; Institute of Molecular and Cell Biology, Agency for Science, Technology and Research, Singapore 138673, Singapore

**Keywords:** hepatocellular carcinoma, intra-tumor heterogeneity, phenotypic evolution, tumor evolution

## Abstract

Intra-tumor heterogeneity (ITH) is a key challenge in cancer treatment, but previous studies have focused mainly on the genomic alterations without exploring phenotypic (transcriptomic and immune) heterogeneity. Using one of the largest prospective surgical cohorts for hepatocellular carcinoma (HCC) with multi-region sampling, we sequenced whole genomes and paired transcriptomes from 67 HCC patients (331 samples). We found that while genomic ITH was rather constant across stages, phenotypic ITH had a very different trajectory and quickly diversified in stage II patients. Most strikingly, 30% of patients were found to contain more than one transcriptomic subtype within a single tumor. Such phenotypic ITH was found to be much more informative in predicting patient survival than genomic ITH and explains the poor efficacy of single-target systemic therapies in HCC. Taken together, we not only revealed an unprecedentedly dynamic landscape of phenotypic heterogeneity in HCC, but also highlighted the importance of studying phenotypic evolution across cancer types.

## Teaser

Using a prospective cohort for Hepatocellular Carcinoma (the PLANET study), this work revealed a dynamic landscape of phenotypic intra-tumor heterogeneity, providing several novel approaches for patient treatment and prognosis prediction.

## INTRODUCTION

Hepatocellular carcinoma (HCC) is the third leading cause of cancer mortality with more than 50% of cases being from Asia [1]. While surgical resection may be curative in patients with early-stage HCC [2], recurrences are common [3,4]. Several recent studies have characterized the genomic landscape [5–8] and identified molecular subtypes as well as potential therapeutic targets for HCC [9,10]. However, no predictive genomic biomarker for systemic treatment has been clinically validated [11]. Currently approved first-line therapies for advanced HCC, namely lenvatinib and sorafenib, confer overall objective response rates (ORRs) of 24% and 9.2% and median overall survival (OS) of 13.6 months and 12.3 months respectively [12]. While combination therapy with atezolizumab (PD-L1 antibody) plusbevacizumab

(vascular endothelial growth factor A/VEGF-A inhibitor) has shown increased efficacy with a reported ORR of 27% in the recent IMBrave150 trial [13], the best systemic therapies for HCC confer ORRs and OS that compare poorly with treatments for other solid organ cancers.

Intra-tumor heterogeneity (ITH) is central to tumor evolution and can contribute significantly to the poor treatment response in HCC [14]. Exploratory studies on small retrospective cohorts have examined the landscape of genomic ITH [15–22] and an intermediate level of DNA ITH was found when comparing HCC with other tumor types [14]. However, most previous studies have focused mainly on the genomic changes in the DNA without systematic exploration of phenotypic evolution. Since phenotypic changes often accompany disease progression, linking multilayer (i.e. phenotypic and genomic) ITH to the clinical trajectory can pave the way for patient treatment and prognosis, but has not been explored in a prospective cohort for HCC.

The Precision Medicine in Liver Cancer across an Asia-Pacific NETwork (PLANET) is a prospective cohort studying the impact of ITH on the clinical trajectory of surgically resected HCC (NCT03267641, Methods). We took advantage of clinical guidelines for HCC in the Asia-Pacific, which recommend surgical resection over a broad pathological range [23,24], providing a unique opportunity to investigate the impact of ITH on the clinical trajectory of resected HCC across AJCC (American Joint Committee on Cancer) pathological stages [25]. Here we report our genomic analysis of ITH in HCC for 67 patients from four countries in the Asia-Pacific. Through multi-region sampling of surgically resected HCC and whole genome sequencing of 331 samples, we found that while DNA ITH stays constant across pathological TNM stages, transcriptome and immune ITH have a rapid increase in ITH in stage II patients. Strikingly, 30% of patients were found to contain more than one RNA subtype within a single tumor (i.e. mixed subtypes), and this occurred in tandem with the transition from less aggressive phenotypes (e.g. low cell cycle) in early-TNM-stage tumors to the more aggressive phenotypes (e.g. upregulated cell cycle) in later-TNM-stage HCC. This phenotypic heterogeneity can significantly reduce the efficacy of monotherapies targeting a small number of lesions, but may confer synergy when combination therapies targeting different dimensions of tumor phenotypes are employed. Through integrative analysis, multiple ITH features, in particular phenotypic ITH, were found to be more informative than genomic ITH in predicting patient prognosis. For the first time, we reveal an unprecedentedly dynamic landscape of phenotypic heterogeneity in HCC, highlighting the importance of studying phenotypic evolution and novel therapies tackling a vibrant landscape of tumor evolution in HCC.

## RESULTS

### Patient recruitment and clinical phenotypes of the PLANET cohort

Through the Asia-Pacific Hepatocellular Carcinoma (AHCC) trials group [26], we enrolled 67 HCC patients from four Asia-Pacific countries (Singapore, Thailand, Malaysia and Philippines, Supplementary Table 1a, Supplementary Note 1) with different ethnic backgrounds: Chinese (*n* = 46), Malay (*n* = 7), Thai (*n* = 4), Indonesian (*n* = 5), Burmese (*n* = 3), Cambodian (*n* = 1) and subcontinental Indian (*n* = 1). As a prospective surgical cohort, patients were enriched for early stage (49.25% in stage I) with intermediate grade (Edmondson grade II and III, Supplementary Fig. 1). More than 60% patients were viral positive cases (59.7% Hepatitis B virus positive, 4.5% Hepatitis C virus positive) with varying degrees of cirrhosis and fibrosis (Metavir score, 20.9% with no fibrosis and 35.8% with cirrhosis, Supplementary Fig. 1). A full description of the patient cohort and clinical phenotypes can be found in Supplementary Note 1 and Supplementary Fig. 1.

### The genomic landscape of the PLANET cohort

**Figure 1.**
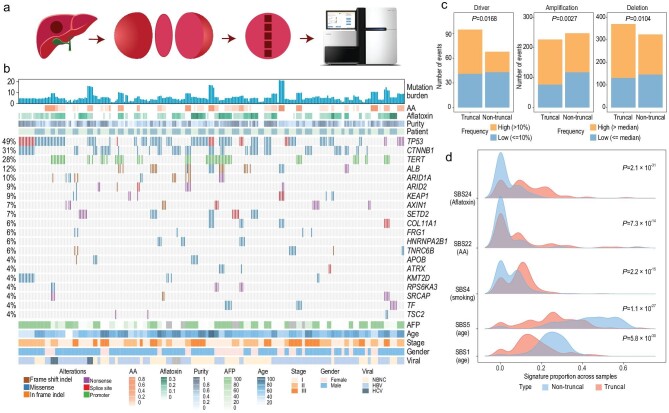
Genomic landscape of the cohort. (a) Schematic representation of the grid sampling. A central slice is taken out from the tumor and consecutive sectors were sampled along a grid line. (b) Oncoprint plot of the common drivers (≥4%) across the cohort (see also Supplementary Fig. 3). Columns are the samples and rows are the genes. Percentage of alterations are shown on the left. Multiple sectors belonging to one patient were annotated (patient, top). Mutation burden is plotted as a bar plot on the top. Clinical features are shown in the bottom annotation panel. (c) The relationship between truncal status and frequency of the somatic alterations (SNVs, amplifications and deletions at the cytoband level). (d) The distribution of signature contributions for the truncal and non-truncal mutations. *P*-values were calculated with two-sided paired Wilcoxon tests.

To survey the degree of tumor heterogeneity, multiple regions (2–11 sectors per tumor depending on the size of the tumor) were harvested using an established grid sampling protocol (Fig. 1a, Methods) [21]. In total, we sequenced 331 samples (264 tumors and 67 adjacent normal tissues) of which 318 samples were subjected to whole genome sequencing (WGS; average depth of 46.7X) and 13 samples were subjected to whole exome sequencing (WES; average depth of 85X, Supplementary Table 1b).

By comparing tumor sectors against their adjacent normal, we characterized Single Nucleotide Variants (SNVs), driver mutations and mutational signatures as well as copy number variations in the PLANET cohort. Even though basic genomic features of HCC have been investigated in several recent studies [5–8], multi-regional sampling provides an important approach with regard to timing genomic changes. We summarize major findings here and present the details in Supplementary Note 2. Firstly, across the 67 patients, the tumor mutation burden (TMB) ranged from 0.5 to 16.3 mut/Mb (median 3.967 mut/Mb, Fig. 1b) with limited variations within each tumor (Supplementary Fig. 2), but large differences between patients. Secondly, by integrating 1349 publicly available HCC genomes, we identified 62 driver genes in HCC using several statistical approaches (Supplementary Note 3) with 48 of the driver genes found in the current cohort (Fig. 1b, Supplementary Fig. 3). While common driver genes such as *TP53* and *CTNNB1* were often shared across all sectors of the same patient (defined as truncal events) across many patients, low frequency drivers such as *FRG1* and *ARID1B* tended to be non-truncal (Fig. 1b, Supplementary Figs 3 and 4). These observations suggest that common driver mutations often arise early in HCC, and rare driver mutations tend to be acquired late during tumorigenesis (Fig. 1c). Thirdly, we identified 13 mutational signatures in the PLANET cohort (Supplementary Note 2, Supplementary Fig. 5, Supplementary Table 1c). Signatures related to environmental stimuli such as aristolochic acid (AA, SBS22), smoking (SBS4) and aflatoxin B1 (SBS24) are more frequent in the early (truncal) part of the evolution (Fig. 1d), implying their higher activity in tumor initiation (Supplementary Fig. 5). Lastly, arm-level copy number alterations (CNAs) were shared across multiple sectors of the same patient (Fig. 1c, Supplementary Fig. 6a, Supplementary Table 1d) [6], suggesting that large chromosomal events are early events in the history of tumorigenesis [27]. However, detailed inspection of focal CNAs revealed that a significant proportion of focal CNAs were subclonal in many patients, driving further diversification in each tumor (Methods, Supplementary Note 2, Supplementary Fig. 6b, Supplementary Table 1e). In summary, the PLANET cohort provided a unique resource for timing genomic changes and revealed many late mutational events in the genetic diversification of HCC.

### Subclonal drivers empower local adaptation in HCC

**Figure 2.**
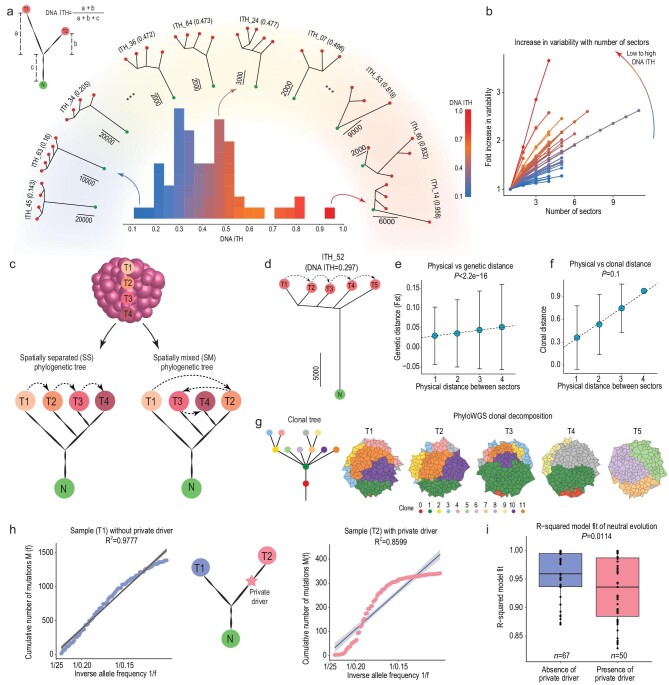
DNA heterogeneity and non-neutral evolution. (a) For any two sectors of a patient, DNA ITH is calculated as the total number of private mutations over the total number of mutations of the two sectors. A wide range of DNA ITH existed across the patient cohort. The histogram in the inner circle displayed the level of DNA ITH. Representative phylogenies from low/medium/high ITH quantiles are shown in the rainbow with color scale ranging from blue (lowest ITH) to red (highest ITH). (b) The relationship between number of tumor samples and the fold increase in the observed variability (see Methods, the same color scale as Fig. 2a). (c) An illustration of spatially separated (SS) and spatially mixed (SM) tree pattern. (d) Sample phylogeny of patient ITH_52. (e) IBD pattern of patient ITH_52. The regression between the physical distances of the patient's sectors (X-axis) and their genetic distance (FST) (Y-axis). (f) Clonal decomposition using PhyloWGS. The regression relationship between the physical distance and cosine distance of the clonal composition of the tumor sectors (Methods). (g) The left panel is the phylogenetic relationship of the clones. Right panel shows the clonal composition of the tumor sectors. (h) Cartoon illustration of the testing of non-neutral evolution in the sample without any private driver (T1) and the sample with private driver (T2). (i) The R-square fit (testing neutral evolution) in samples with private drivers and without private drivers.

Based on the proportion of shared mutations (Fig. 2a), we calculated the degree of tumor heterogeneity for all patients. Across the cohort, we observed a wide range of ITH in DNA ranging from homogeneous tumors (late diversification) to extremely heterogeneous tumors (early diversification) (Fig. 2a, Supplementary Fig. 7, Supplementary Table 2a). High levels of ITH suggests that sampling additional sectors will significantly increase the detected variability (Fig. 2b) and a single biopsy sample will often under-represent the genomic landscape of a patient's tumor.

The unique grid sampling strategy employed in this work allowed us to study the spatial organization of tumor heterogeneity (Fig. 1a). Previous studies in colorectal cancers (CRC) found that subclones within a tumor often distributed in a spatially variegated manner and spatial mixing is a hallmark of cancer progression from adenoma to carcinoma [28,29]. In order to test the presence of spatial mixing, we compared physical locations of the tumor sectors against their phylogenetic relationship (Fig. 2c, Methods) [30]. Surprisingly, only a minor proportion of HCCs showed some levels of spatial mixing (SM, *n* = 10 or 20.4% among 49 patients with at least three sectors, Supplementary Fig.7, Supplementary Table 2b), while for the majority of the tumors, the branching pattern closely matched the physical locations of tumor sectors (e.g. ITH 52 as an example, Fig. 2d). In order to further dissect the spatial organization of ITH in HCC, we applied population genetic [31] as well as clonal deconvolution methods [32,33] across the patients with no spatial mixing (*n* = 39). We found: (i) when we measured the genetic divergence between sectors with varying levels of physical separation, that physically proximal sectors were also genetically more similar (Fig. 2e, *P*-value = 1.2 × 10^–120^), a pattern often known as isolation-by-distance (IBD) in evolutionary genetics [31], and (ii) a clear linear relationship between the physical distance of the sectors and their clonal compositional distance (e.g. for patient 52 in Fig. 2f and g, Supplementary Fig. 7) [32,33]. Taken together, spatial heterogeneity in HCC segregated in an IBD manner and spatial mixing is uncommon in HCC (Supplementary Note 4, Supplementary Fig. 7).

Since spatial mixing is not associated with tumor progression in HCC, we investigated if other evolutionary forces may be driving tumor progression. A few recent studies have described non-neutral evolution across a number of cancer types including lung and colon cancers [34,35], but the situation in HCC remained unknown [35]. Since many driver mutations were specific to subsets of tumor sectors (Fig. 1b) and may drive tumor progression in these tumors via natural selection (i.e. adaptive evolution), we thus explicitly tested the evidence of non-neutral evolution, comparing samples with subclonal driver mutations against their sister samples without private driver mutations (Fig. 2h, Supplementary Table 2c). Using the neutrality test, comparing the variant allele frequency distribution (i.e. site frequency spectrum or SFS) against the prediction from an exponentially growing population [34], we found that samples with private driver mutations tend to have poor fit to the neutral expectation (Fig. 2i and *P*-value = 0.0114), indicating that subclonal drivers are driving adaptive evolution in many tumors. Taken together, we revealed the unique spatial organization of heterogeneity (i.e. IBD) with a significantly underappreciated amount of adaptive evolution in HCC.

### Mixed transcriptomic subtypes in HCC and their evolutionary trajectory

**Figure 3.**
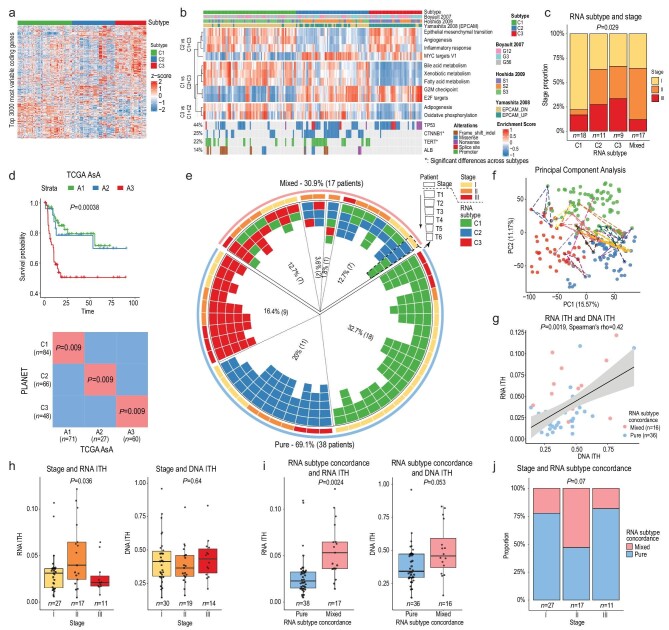
RNA subgroup and mixed subtypes. (a) Heatmap of the expression of the top 3000 MAD coding genes (rows) across the three RNA subtypes (C1, C2 and C3) in all the samples (columns). (b) Enrichment of important functional pathways and major driver mutations across the three subtypes. CGP are liver-related chemical and genetic perturbation gene sets (see Methods). (c) Correlation between RNA subtypes and stages across all samples. (d) Subclass mapping between subtypes in the PLANET cohort and the TCGA Asian cohorts (Methods), and Kaplan-Meier survival analysis of the subtypes in the TCGA Asian cohorts. (e) Circos plot of the RNA subtypes. The Circle shows the RNA subtypes of the tumor sectors (arranged in physical order) of 17 patients with mixed RNA subtypes as well as the pure subtype patients. (f) Principal Component Analysis (PCA) plot of the transcriptome from tumor sectors with lines linking tumor sectors of patients with mixed RNA subtypes. (g) Correlation between DNA ITH and RNA ITH. (h) The relationship between stage and RNA ITH (left) and the relationship between stage and DNA ITH (right). (i) Correlation between mixed subtypes and RNA ITH (left) and DNA ITH (right). (j) The proportion of mixed subtype patients as a function of stage.

The genomic analysis revealed a unique evolutionary trajectory at the DNA level, however it remained unknown how genomic ITH can affect phenotypic evolution [15–22]. Earlier studies have described several transcriptomic subtypes with distinctive clinical and molecular features in HCC [9,36,37]. To dissect the landscape of transcriptomic ITH in HCC, we obtained transcriptomic data from the same sectors of the tumor with WGS (*n* = 55 patients or 198 samples). Using the non-negative matrix factorization (NMF) algorithm, we identified three RNA subtypes (C1-3; Fig. 3a, Methods, Supplementary Table 3a). Gene set enrichment analysis showed that samples belonging to the C1 subtype (*n* = 84) were enriched for metabolic pathways typical of normal liver function and showed up-regulation of pathways associated with better overall survival (Fig. 3b). In contrast, samples belonging to the C2 (*n* = 66) and C3 subtypes (*n* = 48) showed up-regulation of cell-cycle-related pathways and down-regulation of several metabolic pathways (Fig. 3b). Driver genes such as CTNNB1 mutations are enriched in C2 subtype, while TERT mutations are deficient in the C3 subtype. In general, the C1 subtype is enriched for early-TNM-stage patients, while C2/C3 subtypes were more common in later-stage patients (Fig. 3c, *P*-value = 0.029). When we compared RNA subtypes from PLANET with public cohorts including The Cancer Genome Atlas (TCGA), we found very good concordance with two Asian cohorts (Fig. 3d, Supplementary Fig. 8).

Inspecting RNA subtype distributions across patients, 38 of our patients had tumor sectors consisting of a single RNA subtype (i.e. 18 C1, 11 C2 and 9 C3). Surprisingly, the other 17 patients had a coexistence of multiple RNA subtypes across different sectors (i.e. mixed subtypes) (Fig. 3e, Supplementary Table 3a) and these sectors were located very far away in the transcriptomic space (Fig. 3f). Specifically, we observed seven patients with coexisting C1 and C2 subtypes, seven patients with coexisting C1 and C3 subtypes, two patients with coexisting C2 and C3 subtypes and one patient who had all three subtypes within the same tumor (Fig. 3e). The presence of multiple subtypes poses a fundamental question of what evolutionary forces might have led to the coexisting subtypes. One possible reason might be the higher tumor heterogeneity at the DNA level. When we correlated the degree of RNA ITH with DNA ITH, we observed a significant correlation (*P*-value = 0.0019, Fig. 3g, Supplementary Table 3b) controlling for covariates such as tumor purity (Supplementary Fig. 9). However, DNA ITH only contributed a fraction of the transcriptomic heterogeneity (Spearman's rho = 0.42), suggesting other forces could be co-driving the phenotypic evolution in HCC (see Discussion).

In a rapidly expanding population, different clones can drive diversification at the later stage of tumorigenesis, leading to multiple lineages at the time of diagnosis (aka the branched evolution model) [38]. Under this model, the degree of tumor heterogeneity will be higher in advanced-stage tumors. Strikingly, when we stratified our patients by their TNM stages, we observed higher RNA ITH in TNM stage II tumors (Fig. 3h, *P*-value = 0.036). This pattern is consistent when we calculated RNA ITH using different subsets of the transcriptome (e.g. genes positively correlated with tumor purity, Supplementary Fig. 10). Since patients with mixed subtypes often have much higher RNA heterogeneity (Fig. 3i, *P*-value = 0.0024), mixed subtype patients were also slightly enriched in stage II patients (Fig. 3j, *P*-value = 0.07). Given that C2 and C3 tend to be more dominant in later stage tumors (Fig. 3c), mixed subtype tumors may reflect the transitional phase where multiple RNA subtypes coexist in the same tumor as the more aggressive phenotypes (i.e. C2 and C3) become dominant in the tumor during disease progression. Interestingly, we found no differences in the degree of DNA ITH across stages (Fig. 3h) and only a slight increase in DNA ITH in tumors with mixed subtypes (Fig. 3i, *P*-value = 0.053), suggesting that DNA ITH may only contribute partially to the phenotypic evolution in HCC.

### Mixed immune subtypes and the correlation between RNA and immune ITH

**Figure 4.**
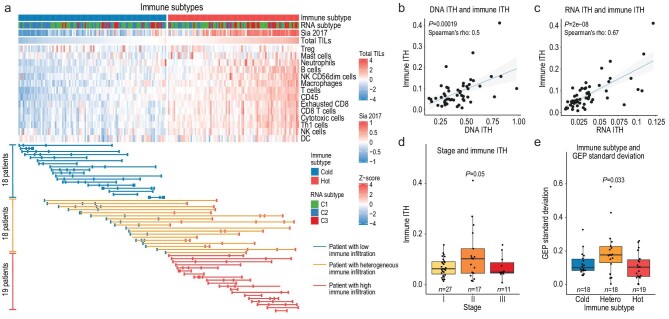
The immune subtype and immune ITH. (a) Tumor sectors are clustered by the level of estimated immune infiltration (Methods). Each row is an immune cell type and each column is a tumor sector. If a patient has all sectors classified as having low levels of immune infiltration (cold, blue), the patient's samples are linked by a blue line. Red or orange lines are used for purely hot or mixed subtype patients. (b) The linear relationship between DNA ITH and immune ITH. (c) The linear relationship between RNA ITH and immune ITH. (d) The relationship between stage and immune ITH. (e) The relationship between stage and the standard deviation of the GEP score.

In order to understand the evolution of the immune microenvironment, we estimated the immune cell composition within a sample and clustered tumor samples into ‘immunologically hot’ and ‘immunologically cold’ tumors (Fig. 4a, Supplementary Table 4a) [39,40]. Interestingly, while the majority of the tumors were either immunologically hot (*n* = 19) or cold (*n* = 18), a significant proportion (*n* = 18, proportion = 33%) of the patients were also immunologically mixed (Fig. 4a). Using estimated immune compositions, we calculated the degree of immune heterogeneity and correlated immune ITH with the heterogeneity at the DNA and RNA levels (Supplementary Table 4b). Interestingly, a significant correlation was observed between immune ITH and genomic ITH, and the correlation is stronger between RNA and immune ITH (Fig. 4b and c) even when we calculate RNA ITH using genes unrelated to immune genes (Supplementary Fig. 11). With immunohistochemistry (IHC) staining, we were able to confirm that transcriptomically hot tumors indeed showed higher immune infiltration (Supplementary Fig. 12) and that genomic ITH correlates with immune ITH with a varying degree of significance (Supplementary Fig. 13).

When we compared the degree of immune ITH across patients of different tumor stages, we found that stage II tumors also showed the highest levels of immune ITH (Fig. 4d, *P*-value = 0.05). When we calculated the GEP score, a pan-cancer predictor for the response to immune checkpoint blockade (ICB) [41], stage II patients also had a higher variance in the GEP score (Fig. 4e, *P*-value = 0.033). In summary, immune ITH had a strong correlation with genomic ITH and attained the highest level in stage II patients.

### Other layers of ITH and their correlation with genomic ITH

One important somatic event for HCC is viral integration. Using BATVI [42], a powerful tool for HBV viral integration, we found that 26 patients from 37 viral positive patients had viral integrations. Two of the patients had integrations only in the adjacent normal tissue. Among the 24 patients with integration in the tumor, we found that a significant proportion of the integrations, especially those in the hotspot regions around *TERT* and *KMT2B* (*MLL4*), were often truncal events that happened in the early history of tumorigenesis (Supplementary Fig. 14). When we calculated integration ITH across patients, we found that heterogeneity in viral integration significantly correlated with DNA ITH (*P*-value = 0.03), but not so significantly with RNA and immune ITH (Supplementary Fig. 14), suggesting that viral integration is an active process along the history of genomic (DNA) changes for HCC with minimum changes to the phenotypic heterogeneity.

Similar to viral integration, when we inferred telomere length as well as fusion gene ITH, we also found significant correlations between genomic ITH and telomere length variation (Supplementary Fig. 15) as well as fusion gene ITH and RNA ITH (Supplementary Fig. 16). In addition to molecular events, when we scored histological heterogeneity using an H&E-stained section of patient tumor slides, we found a positive correlation between histological heterogeneity and genomic ITH even though it did not reach statistical significance due to a limited sample size (*n* = 12, Supplementary Fig. 17). In summary, we revealed a multilayer phenotypic and genomic heterogeneity with high correlation among multiple ITH features.

### Treatment strategies tackling a dynamic landscape of ITH

Our genomic and transcriptomic analyses revealed extensive tumor heterogeneity which may greatly affect current systemic therapies in HCC. To fully dissect possible impacts of ITH, we first explored the heterogeneity of driver mutations with therapeutic potentials [19,43,44]. Using two well-annotated databases, CGI [45] and OncoKB [46], we first curated potential targetable mutations in the PLANET cohort that showed different levels of supporting evidence for their therapeutic potentials. For example, level 1 are mutations in the current clinical guidelines for other indications, while level 2 and 3 are mutations with clinical or pre-clinical evidence respectively, and level 4 are other unconfirmed mutations that occur in the targetable genes (Methods). It is worth pointing out that most of these targetable genes were not derived from HCC, but were from therapeutical implications in other cancer types.

**Figure 5.**
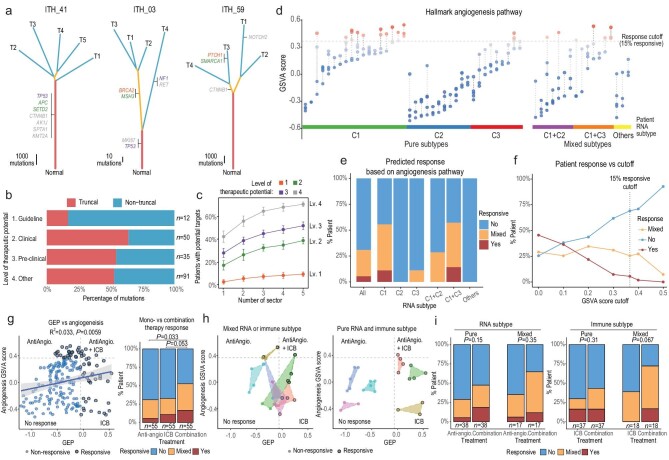
The impact of ITH on possible patient treatment response. (a) Representative patients with varying levels of ITH for potentially targetable mutations are shown. Mutations were classified based on the level of evidence for their therapeutic potential (1, clinically approved for other cancer types; 2, supported by clinical data; 3, supported by pre-clinical data; 4, other mutations in targetable genes, Methods). (b) Proportion of truncal and non-truncal mutations for potentially targetable genes. (c) Proportion of patients found to contain potentially targetable mutations when increasing the number of sectors examined from a tumor (Methods). (d) Activation level shown as the Gene Set Variation Analysis (GSVA) score for the angiogenesis pathway (one of the target pathways for sorafenib and lenvatinib). The upper 15% (response rate) quantile was set as the cutoff value delineating treatment response. (e) The predicted response across patients based on different RNA subtypes. (f) Predicted response rates based on varying levels of cut-off values (Methods). (g) The correlation between the two agents targeted by combination therapy. Based on GSVA and GEP scores, the predicted response to combination therapy for all samples is shown. Samples can be divided into different response quadrants (left) and the corresponding patient-level responses are shown for anti-angiogenesis, ICB and combination therapies (right). (h) Predicted response across sectors for selected patients with high (left) and low (right) phenotypic ITH. (i) Comparison of patient-level predicted response between monotherapies and combination therapy among patients with high and low phenotypic ITH.

Across the PLANET cohort, the degree of ITH for these potentially targetable mutations varied dramatically across patients from being all truncal (ITH_41) to all non-truncal (ITH_59, Fig. 5a, Supplementary Table 4c). Surprisingly, 81.8% of the level 1 mutations were subclonal (Fig. 5b), which seems to be much higher than common HCC drivers (Fig. 1c, Supplementary Fig. 4). Even though targetable mutations for drugs outside the clinical guideline had higher truncal proportions, a substantial proportion of these mutations remained subclonal (Fig. 5b, Supplementary Fig. 18). High subclonality also seems to be true for copy-number-based biomarkers. For example, *FGF19* amplification, a biomarker for FGFR inhibitors in clinical trials [47,48], had a high level of heterogeneity with more than 60% of the amplification being subclonal (Supplementary Fig. 6). In view of such high genomic ITH, increasing the number of samples from a tumor would increase the chance of therapeutic targets (Fig. 5c), which might significantly improve the poor performance in the biomarker-based treatments in HCC.

In addition to genomic heterogeneity, transcriptomic heterogeneity may also pose a serious challenge to current treatment strategy. For example, first-line systemic tyrosine-kinase inhibitors (TKI) for HCCs target important pathways such as the angiogenesis pathway [49], which was unevenly expressed across the tumor sector. Concordant with the transcriptomic subtype analysis, patients with C2 subtype showed a low activation level of the angiogenesis, and a much higher ITH in the angiogenesis pathway was observed in patients with mixed RNA subtype (*P*-value = 0.0039). Assuming tumors with low activation levels would not respond to TKIs, a cut-off for treatment response was set to match the reported response rate of 15% (Methods) [13]. With the high transcriptomic ITH, only 5.5% of the patients were predicted to be responsive for all sectors while 25.5% of the patients would show mixed responsiveness (Fig. 5d and e). Such mixed responsiveness was found to be rather invariant to the cut-off values used (Fig. 5f), indicating mixed treatment response as a general property of the high phenotypic ITH. A qualitatively similar trend was found when we applied the same analysis to other targets of first-line systemic TKIs, as well as immunotherapies (Supplementary Fig. 19). In summary, we found that high phenotypic heterogeneity in HCC could lead to a mixed response for a wide range of therapeutic targets.

Recently, combination therapies targeting both the angiogenesis pathway and ICB have shown great potential to improve patient response [13], yet the impact of phenotypic ITH on combination therapy remains unknown. Notably, we observed only a weak correlation between the targets of these two agents (Fig. 5g), suggesting that the response of this combination therapy may be rather independent. Interestingly, such orthogonality did increase the predicted response rates for combination therapy compared to monotherapy (Fig. 5g). For example, patients with C2 subtype would be expected to show a low response rate for first-line TKIs, yet a substantial proportion of them contained sectors likely responsive to ICB, especially patients with mixed subtypes (Supplementary Fig. 19). Interestingly, patients with mixed subtypes seem to have a higher chance of containing responsive sectors for combination therapies due to high dispersion in the transcriptomic landscape (Fig. 5h and i). Thus, heterogeneity may not always play an adverse role in affecting the response rate in the case of combination therapies. Taken together, combining treatments targeting orthogonal pathways can increase the overall response rate across a wide range of patients, providing a unique strategy for tackling the extremely high ITH in HCC.

### Tumor heterogeneity contributes significantly to patient prognosis

**Figure 6.**
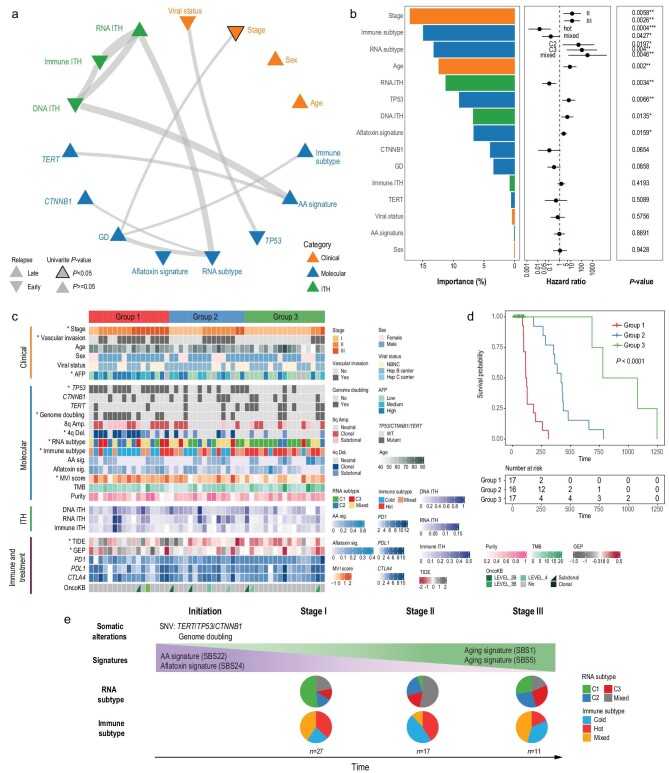
Integrative survival analysis and natural history of HCC evolution. (a) Correlation network of the selected clinical, molecular and ITH features. The edges of the network indicate correlation between features (thicker lines indicate smaller correlation *P*-values). Upward triangles represent a hazard ratio (HR) <1 (later recurrence) and downward triangles represent an HR >1 (earlier recurrence). For features with multiple levels such as stage, the HR of the most significant level is used. A black border around the triangle indicates significance (log-rank score test *P*-value < 0.05) in the univariate Cox model. (b) Ranking of importance among variables in the multivariate Cox model. HRs and *P*-values from the multivariate Cox-model are also shown. (c) Survival groups predicted using the multivariate Cox model. Asterisks indicate that the feature is significantly correlated to the predicted subgroups. Immune markers and treatment options were not used in the Cox model but are shown as annotations. (d) Kaplan-Meier curves for the predicted survival groups. (e) Schematic representation of the natural history of HCC evolution with key events in different clinical stages. Pie charts show the patient-level proportion of RNA and immune subtypes across different stages from the same set of patients used in the above Cox models.

From multilayer heterogeneity, we found that both RNA and immune ITH strongly correlate with disease progression, suggesting important potentials using multiple ITH features for patient prognosis. In order to combine multiple sets of information in patient prognosis, we first explored whether the degree of multilayer ITH correlates with other features of tumor biology, including clinical and molecular features (e.g. driver mutation status). Using four fundamental clinical features (e.g. stage), eight molecular features (e.g. RNA subtype) and three ITH features (degree of DNA, RNA and immune ITH) (Fig. 6a, Supplementary Table 5a), we computed pairwise correlations among all these features (e.g. Supplementary Fig. 20) and found significant correlations between ITH features as well as between ITH features and other types of features. These observations suggest that the degrees of ITH are not fully independent of other layers of information and ITH features can be integrated with other clinical and molecular phenotypes for integrative survival analysis.

In order to combine information across layers, we constructed a multivariate Cox model to integrate all the features, and stratified patients into three subgroups based on recurrence-free survival (RFS) (Fig. 6b–d, Supplementary Fig. 21, Supplementary Table 5b). Multiple molecular and clinical features (e.g. stage or RNA subtype) tend to be distributed unevenly across the three subgroups (Fig. 6c), suggesting distinctive phenotypes across survival subgroups. Interestingly, while TNM stage remains an important predictor of RFS, multiple ITH and molecular features also contribute significantly to the survival model (Fig. 6b). In order to explicitly test the importance of the ITH features, we compared a baseline model without ITH features with a full model with ITH features (Fig. 6b, Supplementary Note 5). The multivariate Cox model with ITH features performed much better than the baseline model (paired t-test *P*-value = 7 × 10^–4^, based on Harrell's concordance index (c-index) or *P*-value = 0.015 based on the likelihood ratio test, Supplementary Fig. 22). In summary, with the largest prospective cohort studying intra-tumor heterogeneity for HCC, we found that multiple ITH features form an important layer of information, which contributes significantly to patient prognosis and survival.

## DISCUSSION

Using one of the largest prospective surgical cohorts with multi-region sampling for HCC, we have dissected the degree of ITH across multiple layers and provided several novel insights into the evolution and treatment of HCC. First of all, this study demonstrated the importance of studying phenotypic evolution, a pivotal layer under-studied in many previous studies. In contrast with an intermediate level of DNA ITH found for HCC [14], the phenotypic heterogeneity seems to be rather high. Even in HCC treated with surgical resection, a significant proportion of the tumors (∼50%) already carry biologically aggressive RNA subtypes (∼25% as mixed subtypes, ∼25% as advanced C2/C3 subtypes, Fig. 6e). Using a classical approach from evolutionary genetics, when we model gene expression patterns across multiple sectors using an Ornstein-Uhlenbeck process, we indeed found stronger statistical evidence for a model with multiple expressional levels for many patients with mixed subtypes (Supplementary Fig. 23, Supplementary Note 6, Supplementary Table 6). Since genomic ITH only explains 42% of the total variability in transcriptomic heterogeneity (Fig. 3g), this low correlation might have allowed the genotypic (DNA) and phenotypic (RNA) ITH to decouple from one another and evolve in different trajectories (Fig. 6e). When we tested the correlation between multilayer ITH and patient clinical features (e.g. viral status), no significant correlation was found, suggesting that HCC etiology might not be a strong determinant of tumor heterogeneity. Thus, the study of phenotypic evolution opens new directions for future studies identifying important factors (e.g. epigenetic changes in cellular plasticity or tumor microenvironment changes) and mechanisms that might drive rapid phenotypic evolution within HCC (Supplementary Note 6). This posits the interesting question of how phenotypic evolution could have occurred in other cancer types and whether HCC is a phenotypically more heterogeneous cancer type. In summary, our study not only revealed an unprecedentedly dynamic landscape of phenotypic heterogeneity in HCC, but also highlighted the importance of studying phenotypic evolution across cancer types.

Secondly, the spatial sampling of tumor sectors revealed an IBD pattern where different parts of the tumor and the immune microenvironment evolve and subsequently attain different phenotypic subtypes within a single tumor (Fig. 6e). Such spatial segregation could allow subclonal driver mutations to reside in different locations of the tumor, further driving local adaptation. To our knowledge, this dynamic phenotypic evolution and coexistence of multiple phenotypic subtypes (RNA and immune subtypes) has not been previously reported in any cancer type. Previous population genetic modeling suggested that the IBD pattern can be compatible with many evolutionary scenarios (e.g. different growth models, Supplementary Note 7) [21]. Thus, the study of spatial heterogeneity in HCC provided a unique model for tumor evolution, worth testing in other cancer types.

Finally, the heterogeneous genomic and transcriptomic landscape of HCC might explain why monotherapies targeting alterations suggested by a single biopsy have been so poor in HCC [50]. As monotherapies might not be able to target heterogeneous parts of HCC, combination therapies targeting multiple vulnerabilities of the tumor can yield better outcomes. Using anti-angiogenesis and ICB therapies as an example, we illustrated how combination therapy on weakly correlated targets could have improved the treatment response in a heterogenous landscape like HCC (Fig. 5g–i). Moreover, treatment responses would be affected by both the mean expression level of drug targets and the level of ITH across sectors. A homogenous tumor with low expression of the target may not respond to treatment at all, while patients with highly heterogeneous tumors may benefit more from combination therapies due to higher dispersion on the transcriptomic landscape (Fig. 5h). In sum, the PLANET cohort provided a unique resource for the community to explore possible new combination therapies tackling an unprecedentedly heterogeneous landscape, further improving personalized treatment in HCC.

## MATERIALS AND METHODS

### Patient recruitment and spatial sampling

Sixty-seven patients were recruited from six regional hospitals from the AHCC trial group. A full set of patient recruitment criteria is described in Supplementary Note 1. The PLANET study was approved by Singhealth Centralized Institutional Review Board (2016/2626 and 2018/2112) and informed consent was taken from each patient before enrollment.

### Tissue sampling and genomic sequencing

A single slice was harvested in the tumor through the capsule, and multiple sectors (regions) along one axis of the tumor were then harvested. Non-tumor liver tissues (≥2 cm away) from the tumor were also harvested. Genomic DNA and mRNA were extracted from the patient samples and subsequently sequenced at Novogene-AIT Inc. and the Genome Institute of Singapore.

### Genomic analysis

Raw genomic data followed read mapping, mark duplicates, realignment, recalibration and variant calling (Supplementary Methods). Signature analysis was conducted using the NMF method. Viral integration was identified using BATVI [42] and telomere length was estimated using the TelSeq method. Potentially targetable mutations were annotated using CGI [45] and OncoKB [46].

We measured the level of tumor heterogeneity in DNA (DNA ITH) as the number of private mutations divided by the total number of mutations (Fig. 2a). Using the list of somatic mutations called from each sample, we calculated the hamming distance between all sample pairs and inferred the phylogenetic relationship between tumor samples using the Neighbor-joining algorithm [30]. We used the unbiased estimator from Weir and Cockerham 1984 to estimate F statistic (FST) [31]. PyClone [33] and PhyloWGS [32] were used for the clonal decomposition. We computed cancer cell fraction (CCF) for all mutations, adjusting tumor purity and copy number using the method provided in R package EstimateClonality, available at https://bitbucket.org/nmcgranahan/pancancerclonality/src/master/. A neutrality test was conducted as the linear regression (i.e. goodness of fit) between 1/VAF (variant allele frequency) and the number of cumulative mutations.

### RNA analysis

RNA sequence data followed an in-house pipeline (https://github.com/gis-rpd/pipelines). We selected the top 3000 most variable coding genes based on their median absolute deviation (MAD) across the cohort for RNA clustering. NMF clustering and bootstrapping were used to assign subtypes to samples for each patient. SubMap (http://software.broadinstitute.org/cancer/software/genepattern/) was used to measure the similarity between different clustering results. Gene set enrichment analysis was carried out using the GSVA package in R. For each patient, Spearman distances (1-Spearman correlation) between the coding gene expression of all pairwise tumor samples were computed. The mean of all pairwise sector distances was taken as the RNA ITH value of the patient. FusionCatcher was used to identify fusion genes. Immune cell populations of tumor samples with available RNA-seq data were estimated using the method of Danaher *et al*. [39].

### Feature correlation and integrative survival analysis

For testing correlation among variables, Fisher's exact test, linear regression or Kruskal–Wallis test were used. The multivariate survival model was implemented in the coxph function in R. Harrell's concordance index (c-index) was calculated using the concordance.index function from the survcomp R package. A full description of all the methods is given in Supplementary Methods.

## DATA AVAILABILITY

The raw data for this study have been deposited in the European Genome-Phenome Archive (EGA, http://www.ebi.ac.uk/ega/) under accession code EGAS00001003813. All clinical records, somatic mutations, copy number variations and raw expression counts from our study are hosted in OncoSG (https://src.gisapps.org/OncoSG/) under dataset ‘Hepatocellular Carcinoma (GIS, 2020)’, which is publicly available.

## Supplementary Material

nwab192_Supplemental_Files
