# Supplementary material for: Dynamic phenotypic heterogeneity and the evolution of multiple RNA subtypes in hepatocellular carcinoma: the PLANET study
Source: Natl Sci Rev. 2021 Oct 29;9(3):nwab192. doi: 10.1093/nsr/nwab192 (PMC8973408; doi:10.1093/nsr/nwab192)
Supplement: nwab192_Supplemental_Files [file nwab192_supplemental_files.zip › Supplementary_Figures.pdf]

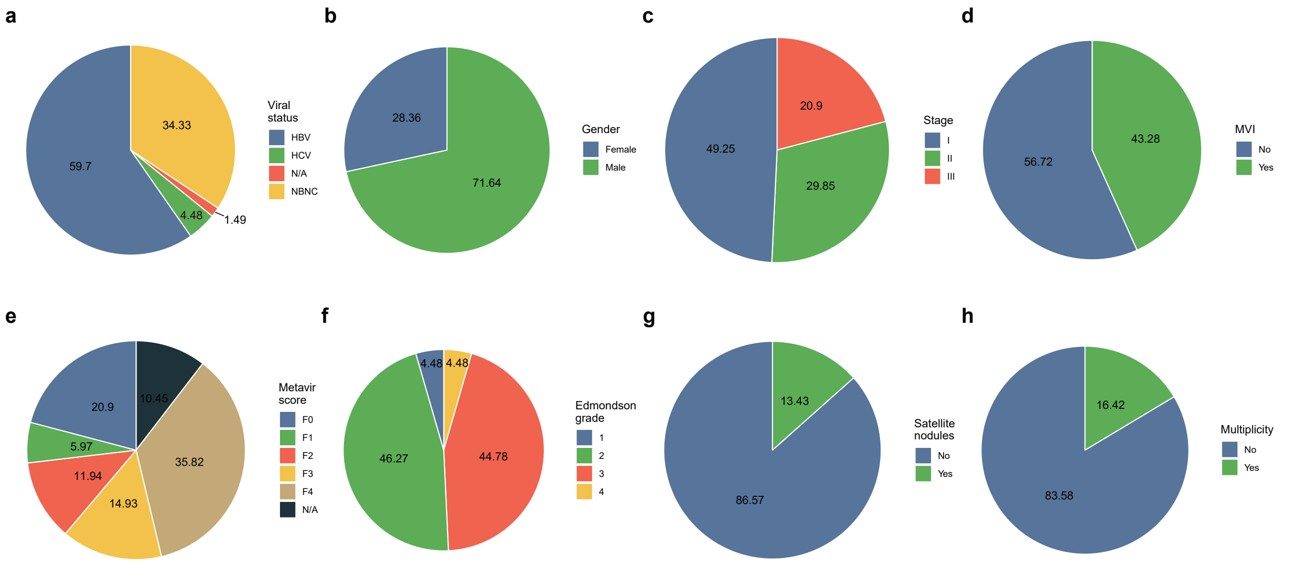

Supplementary Figure 1: **Clinical phenotypes of the patient cohort.** The clinical distribution of the patient cohort, a) viral status, b) gender, c) stage, d) MVI status, e) Metavir score, f) Edmondson grade, g) Satellite nodule, h) Multiplicity

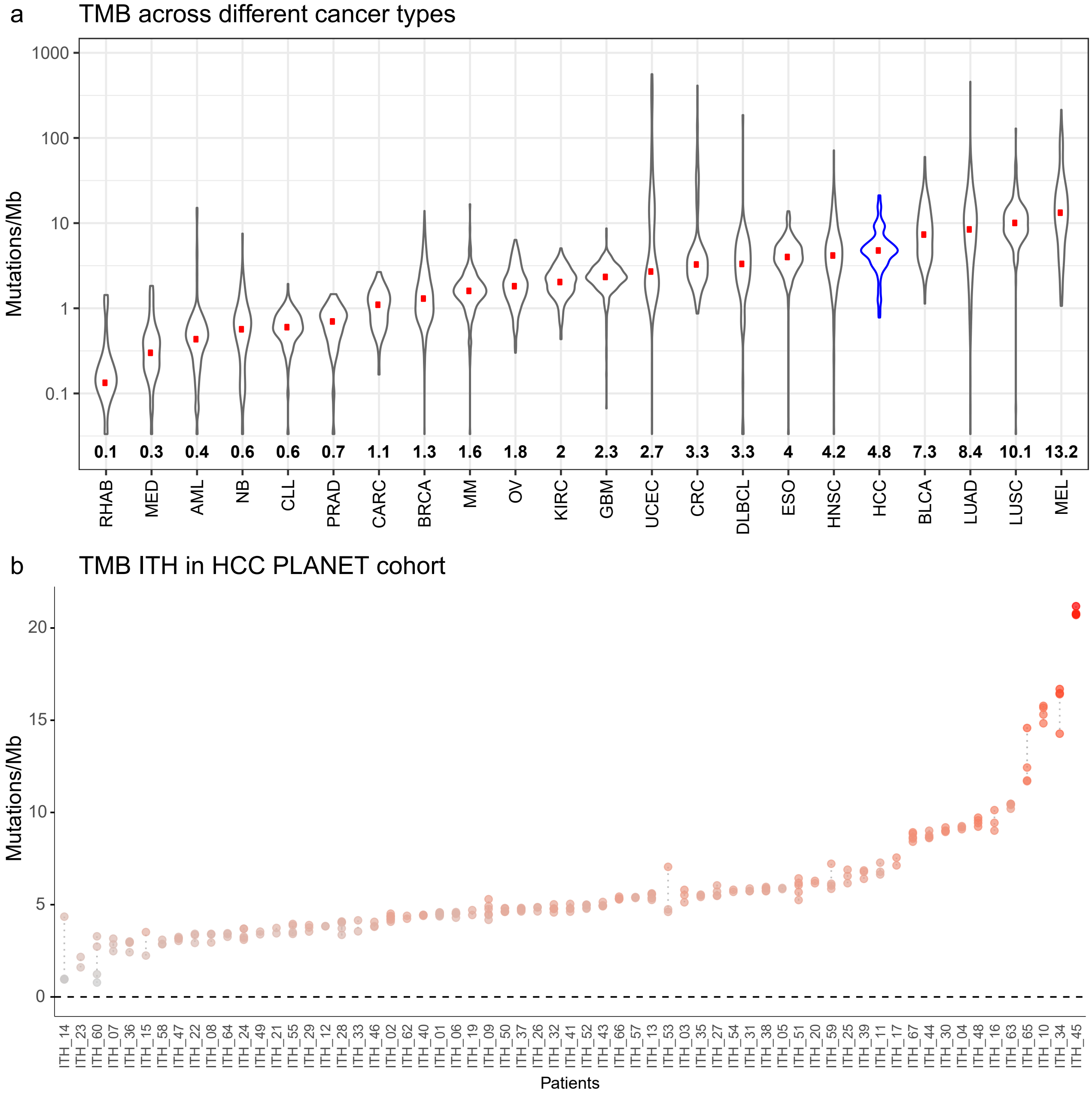

Supplementary Figure 2: **Mutation burden landscape.** (a) Total mutation burdern (TMB) from different cancer types. Mutation burden across many cancer types including HCC from the PLANET cohort. Numbers at the bottom are the median number of mutations per Mb. Mutation data for other cancer types were extracted from http://www.tumorportal.org/. AML, acute myeloid leukemia; BLCA, bladder carcinoma; BRCA, breast cancer; CARC, carcinoid; CLL, chronic lymphocytic leukemia; CRC, colon and rectal cancer; DLBCL, diffuse large b-cell lymphoma; ESO, esophageal adenocarcinoma; GBM, glioblastoma multiforme; HCC, hepatocellular carcinoma; HNSC, head and neck; KIRC, kidney clear cell carcinoma; LUAD, lung adenocarcinoma; LUSC, lung squamous cell carcinoma; MED, medulloblastoma; MEL, melanoma; MM, multiple myeloma; NB, neuroblastoma; OV, ovarian cancer; PRAD, prostate adenocarcinoma; RHAB, rhabdoid tumor; UCEC, corpus endometrial carcinoma. (b) TMB ITH across patients in the HCC PLANET cohort. Each dot represent the TMB level from a sector. Only samples with purity of over 0.3 were plotted to avoid bias from low purity samples.

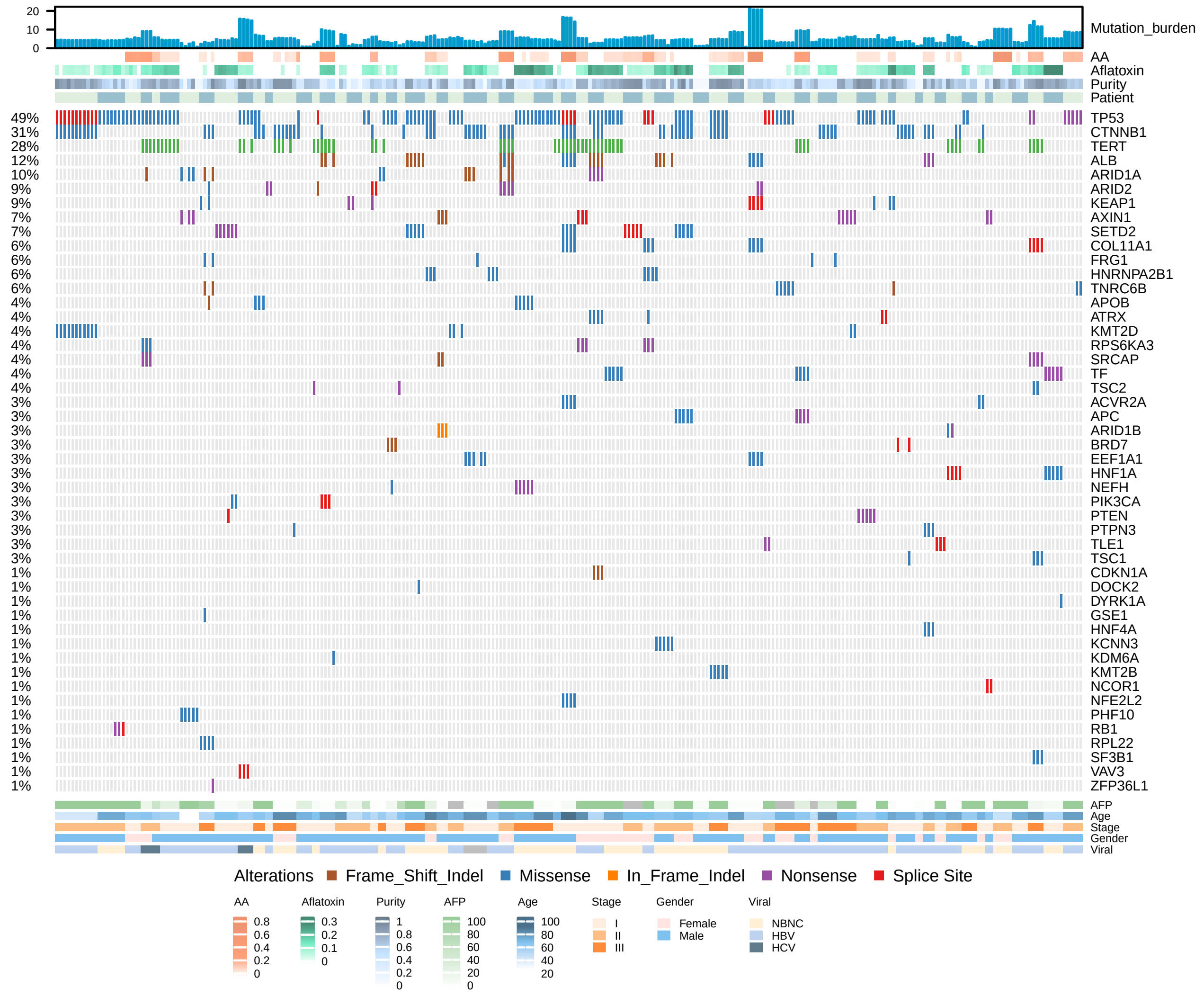

Supplementary Figure 3: **Oncoprint plot for all driver genes in the PLANET cohort.** Full version of oncoprint plot across the PLANET cohort for all driver genes (n=48). Rare drivers (<4%) are also shown in this plot (not in Figure 1b).

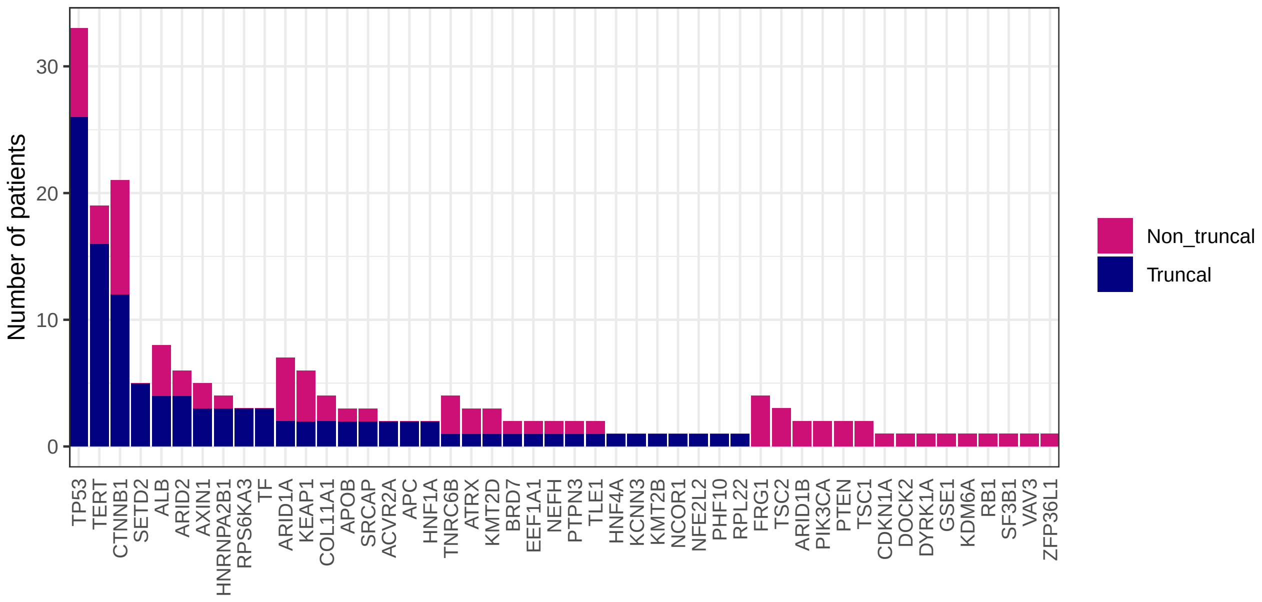

Supplementary Figure 4: **Clonal status of HCC drivers in the PLANET cohort.** Barplot showing the number of patients with truncal and non-truncal mutations in each driver gene. A driver is designated as truncal driver if it is mutated in all sectors, and as non-truncal driver otherwise.

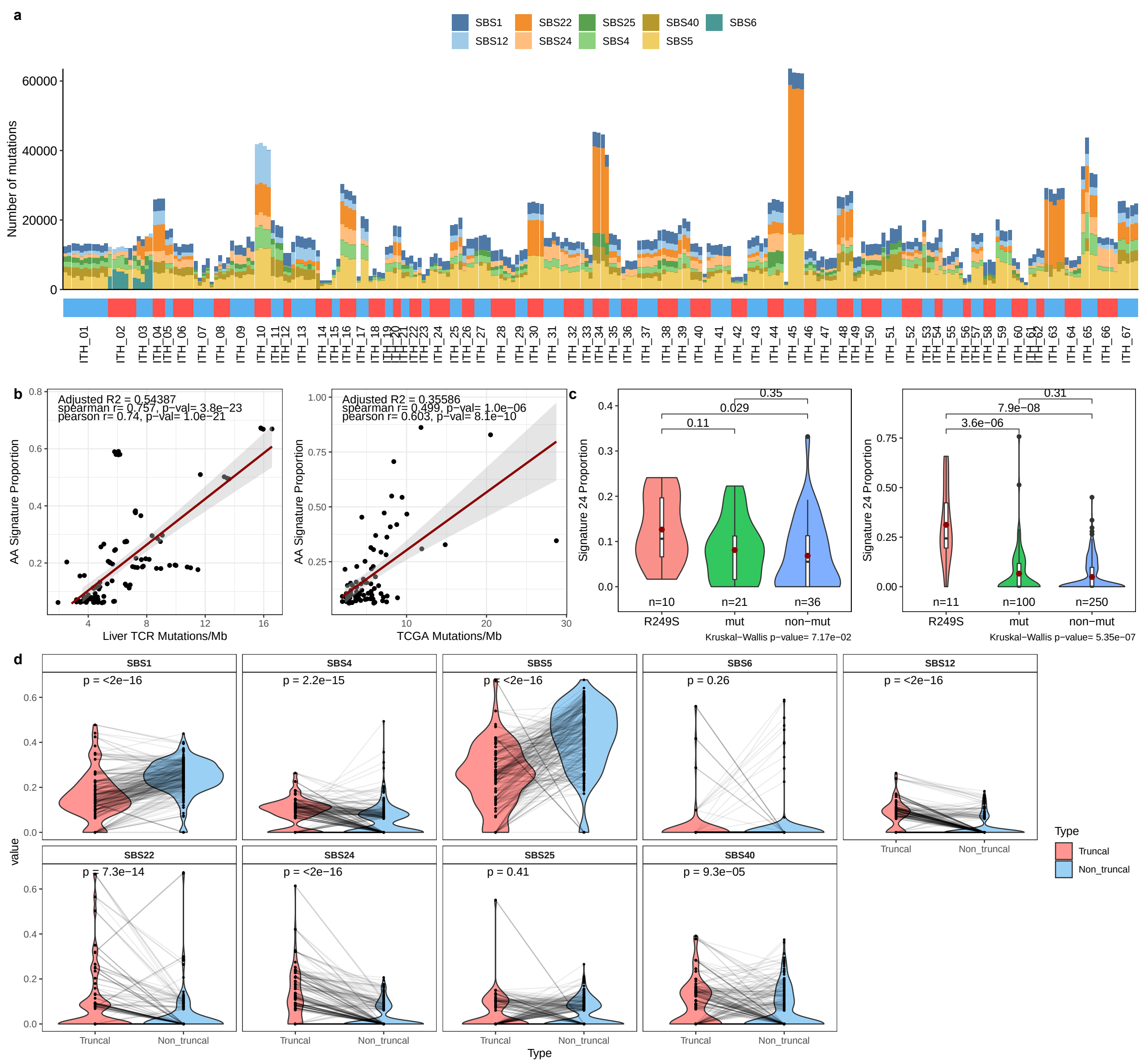

Supplementary Figure 5: **Mutational signatures.** (a) Number of mutations attributed to each signature type across samples. (b) Correlation between mutation burden and AA signature proportion in this study (left) and in the TCGA cohort (right). (c) Comparison of Aflatoxin B1 signature (SBS24) proportion in TP53-R249S mutated samples, other TP53 mutated samples and TP53 wild type samples in this study (left) and in the TCGA cohort (right). (d) Boxplots showing signature proportion changes in truncal and non-truncal mutations. P-value is calculated using two-sided Wilcoxon test.

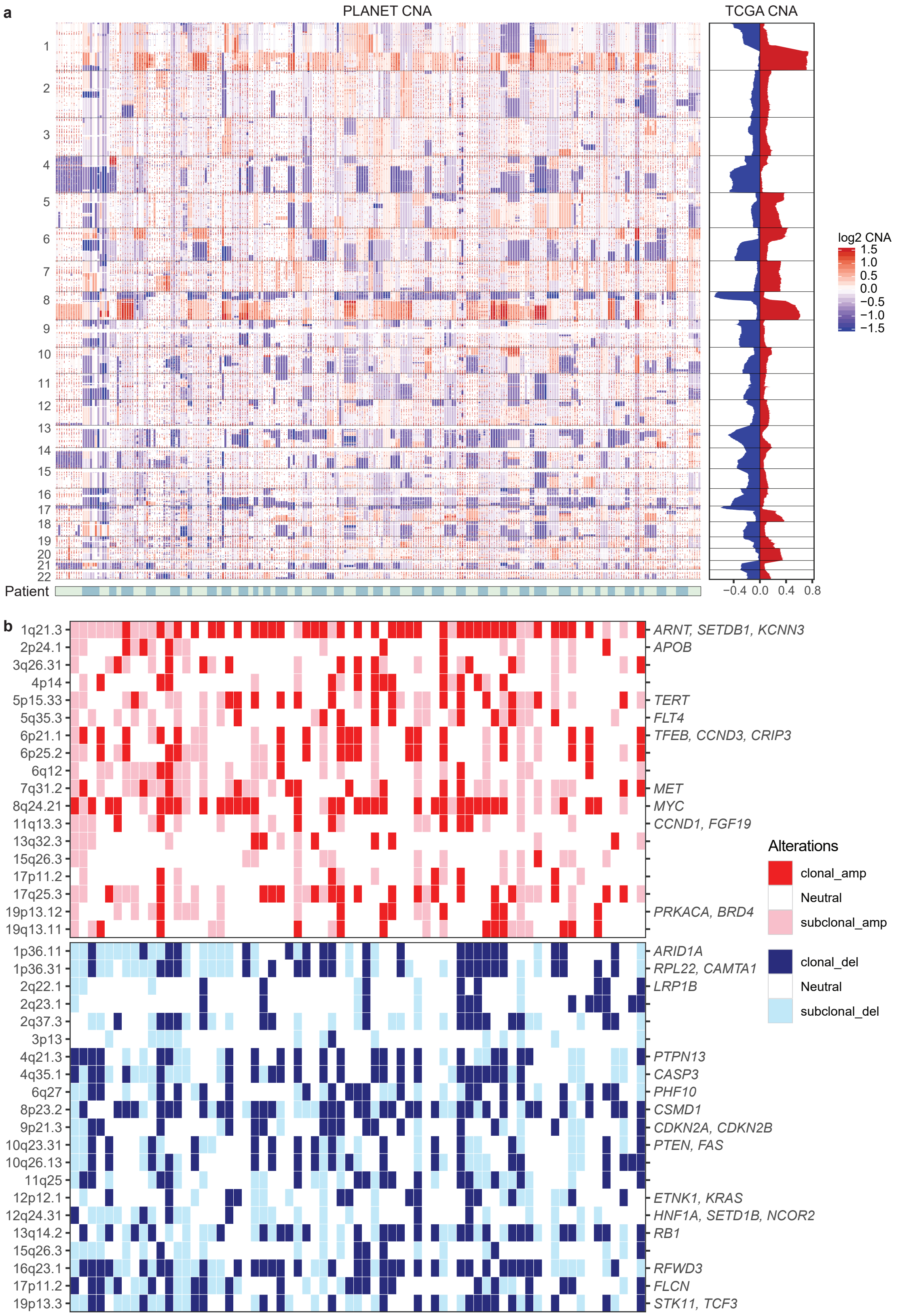

Supplementary Figure 6: **Copy number alterations in the PLANET cohort.** (a) Genome-wide copy number landscape of the PLANET cohort. Columns are samples and rows are chromosomes. Patient info is indicated at the bottom. The frequencies of CNV events in the TCGA cohort are shown on the right. (b) Clonality of the amplifications (top, red) and deletions (bottom, blue) of cytobands as inferred by GISTIC. Clonal (dark colors) and subclonal (light colors) amplifications and deletions are shown for each patient (column). Important COSMIC driver genes are listed on the right.

Supplementary Figure 7: Clonal deconvolution of the patient cohort (page 6-39 , see figure legend on page 39).

Patient 1: ITH_01

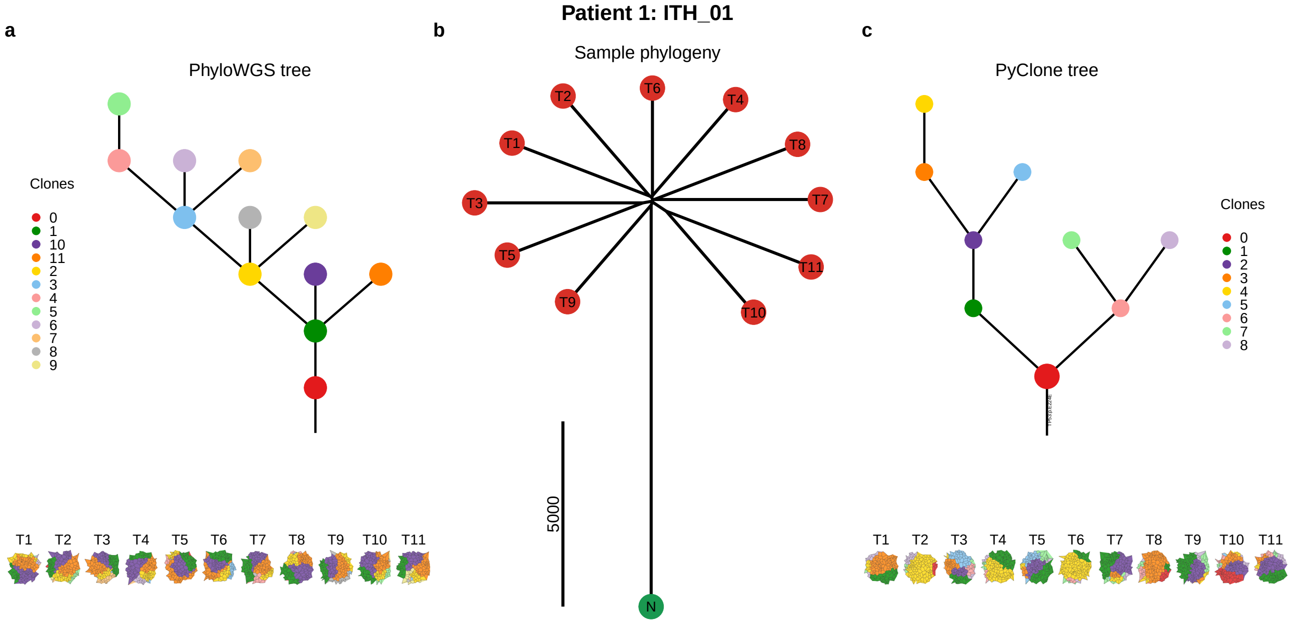

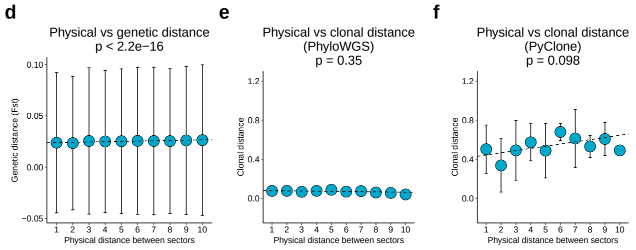

g

Clinical phenotypes

PID	Age	Sex	Race	Stage	TumorSize	Recurrence	AFP	MVI	HBV	HCV
ITH_01	43	Male	Malay	II	9	Yes	110	Yes	Pos	Neg

Patient 2: ITH_02

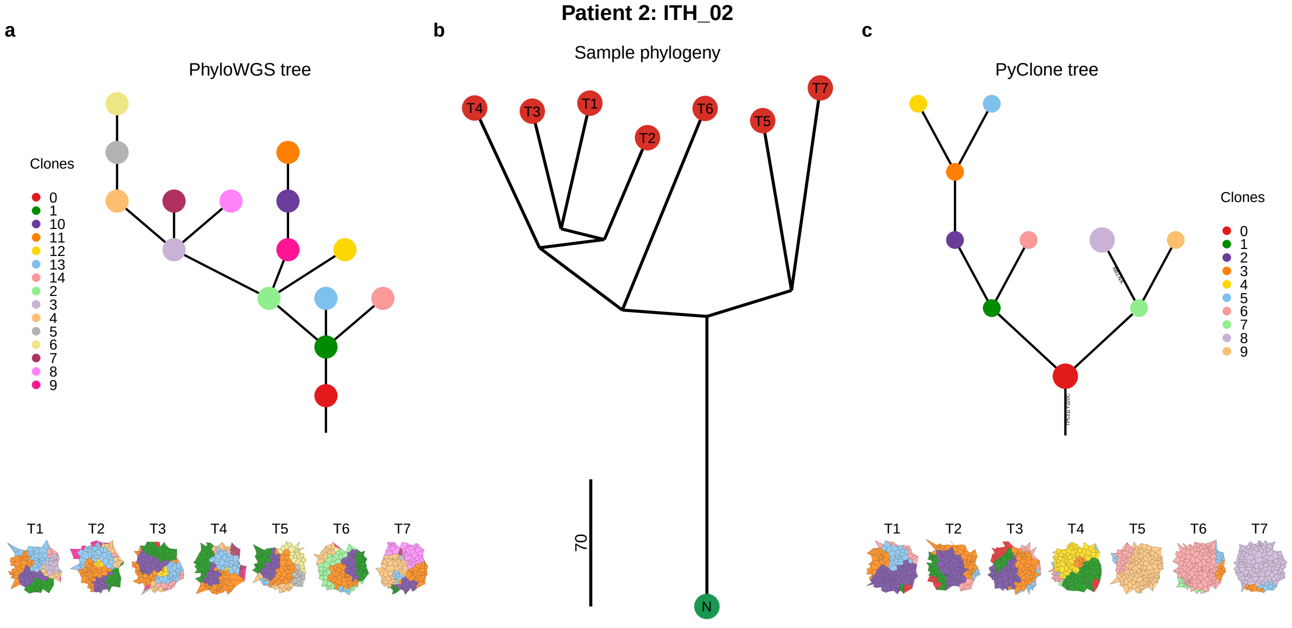

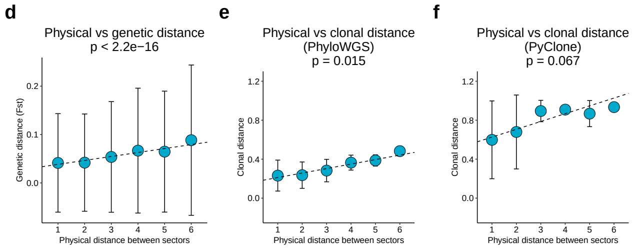

g

Clinical phenotypes

PID	Age	Sex	Race	Stage	TumorSize	Recurrence	AFP	MVI	HBV	HCV
ITH_02	72	Male	Indonesian Chinese	II	14	Yes	348	Yes	Neg	Neg

Patient 3: ITH_03

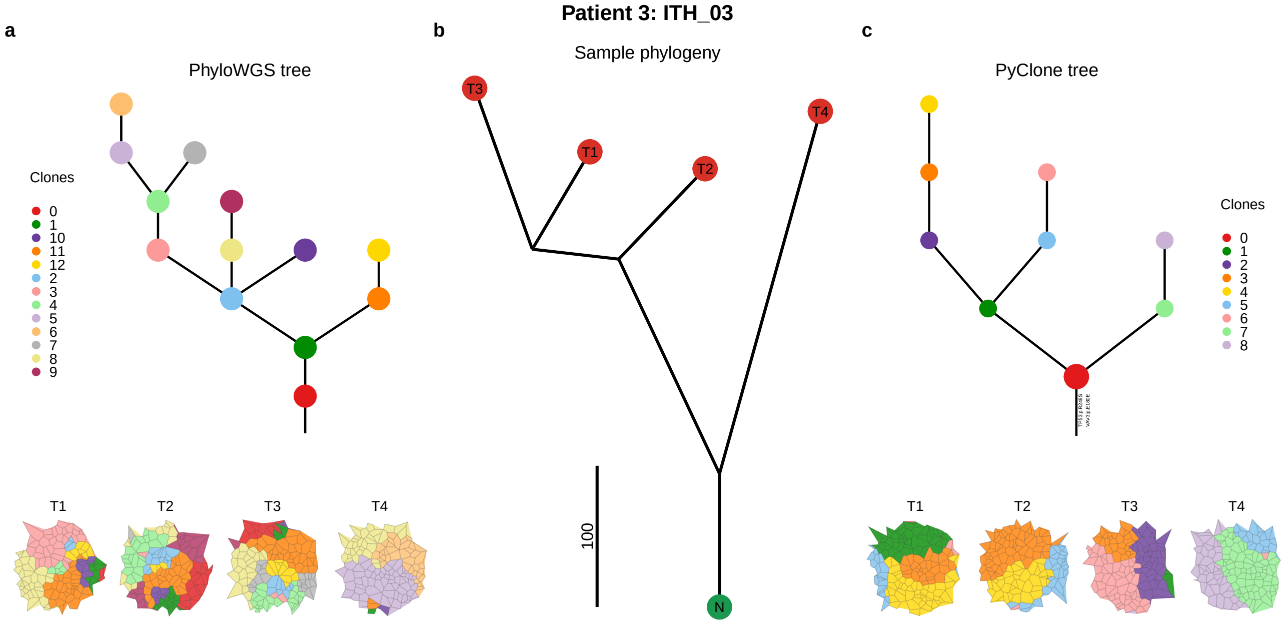

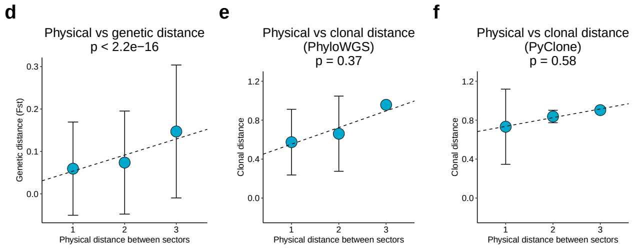

g

Clinical phenotypes

PID	Age	Sex	Race	Stage	TumorSize	Recurrence	AFP	MVI	HBV	HCV
ITH_03	62	Female	Indonesian Chinese	I	5.2	No	2544	No	Pos	Neg

Patient 4: ITH_04

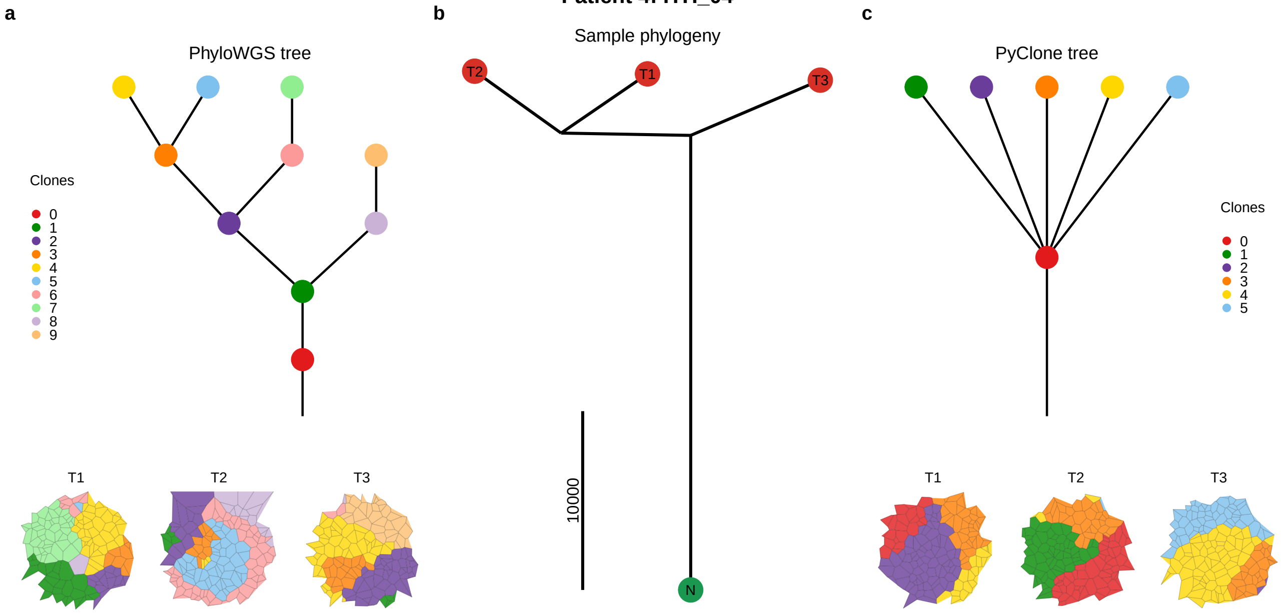

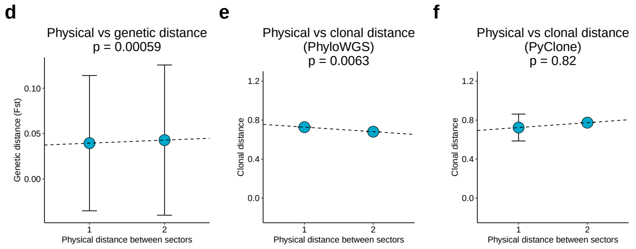

g

Clinical phenotypes

PID	Age	Sex	Race	Stage	TumorSize	Recurrence	AFP	MVI	HBV	HCV
ITH_04	64	Female	Burmese	II	6	NA	20.1	Yes	Neg	Pos

Patient 5: ITH_05

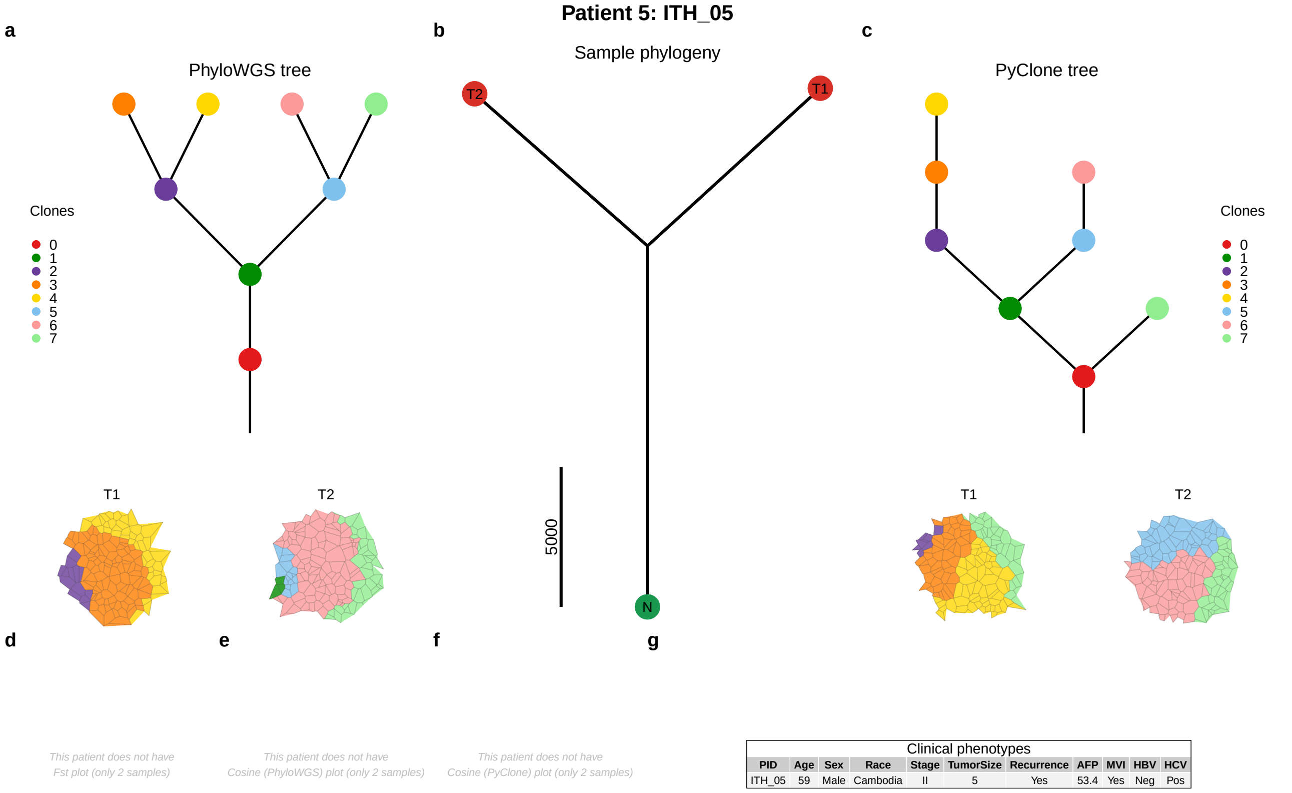

Clinical phenotypes

PID	Age	Sex	Race	Stage	TumorSize	Recurrence	AFP	MVI	HBV	HCV
ITH_05	59	Male	Cambodia	II	5	Yes	53.4	Yes	Neg	Pos

Patient 6: ITH_06

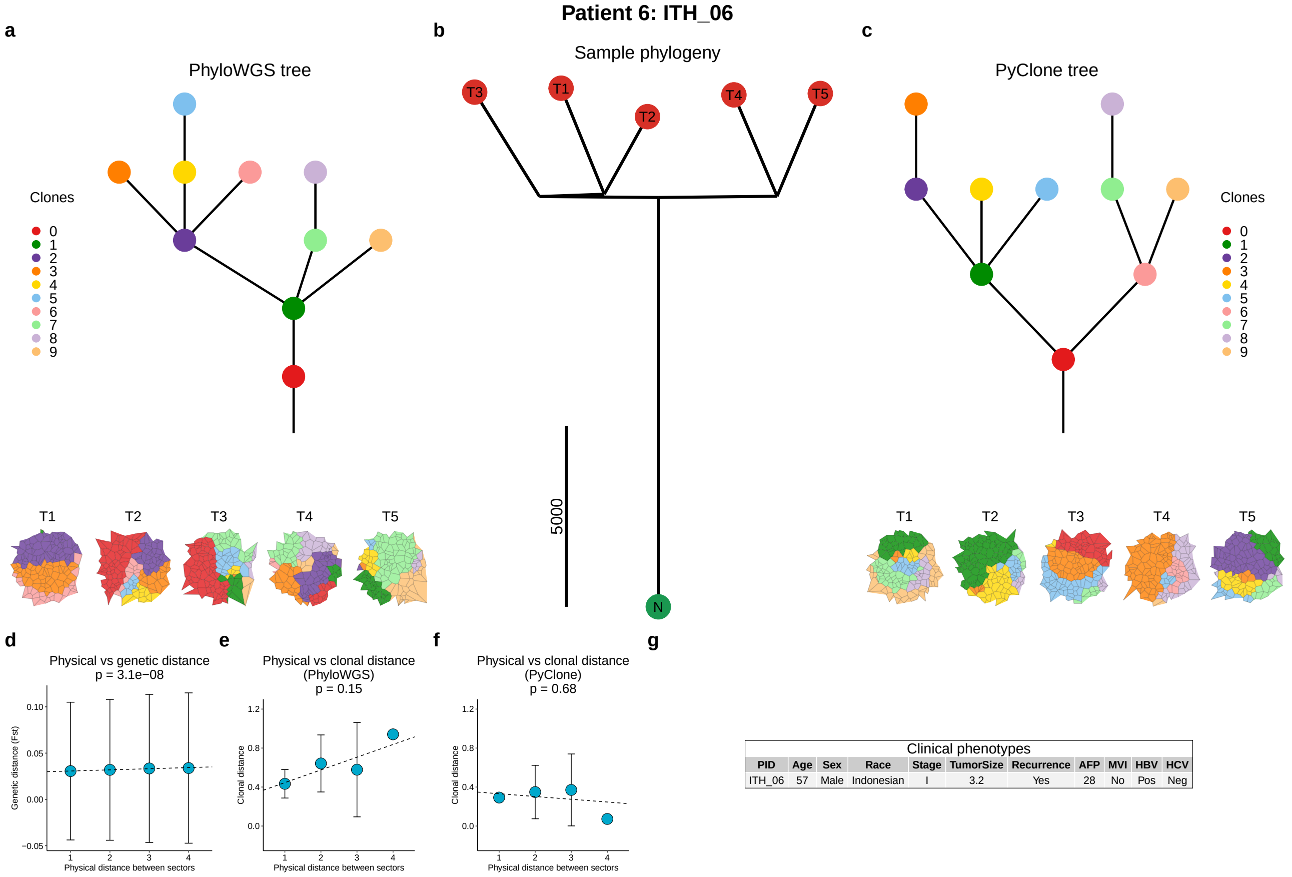

Clinical phenotypes

PID	Age	Sex	Race	Stage	TumorSize	Recurrence	AFP	MVI	HBV	HCV
ITH_06	57	Male	Indonesian	I	3.2	Yes	28	No	Pos	Neg

Patient 7: ITH_07

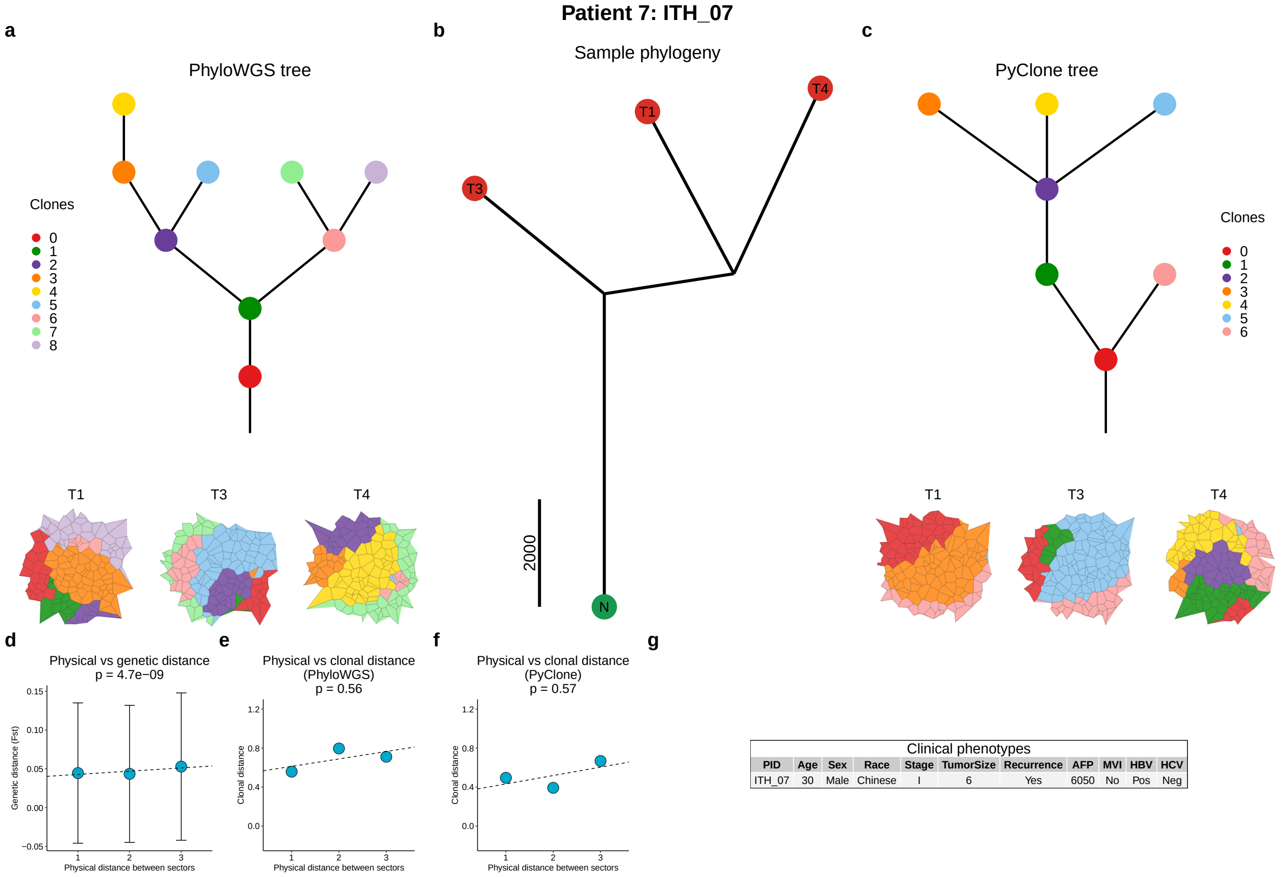

Clinical phenotypes

PID	Age	Sex	Race	Stage	TumorSize	Recurrence	AFP	MVI	HBV	HCV
ITH_07	30	Male	Chinese	I	6	Yes	6050	No	Pos	Neg

Patient 8: ITH_08

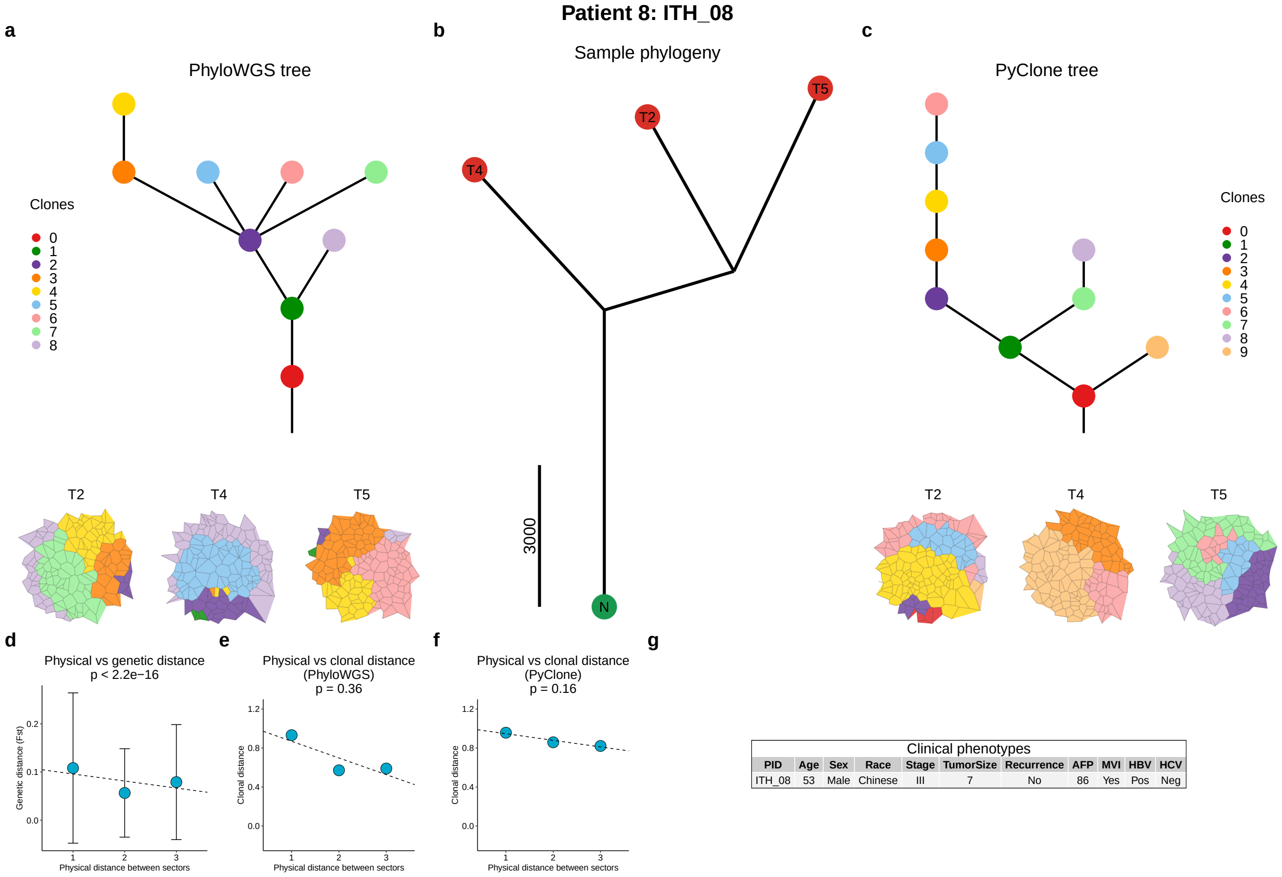

Clinical phenotypes

PID	Age	Sex	Race	Stage	TumorSize	Recurrence	AFP	MVI	HBV	HCV
ITH_08	53	Male	Chinese	III	7	No	86	Yes	Pos	Neg

Patient 9: ITH_09

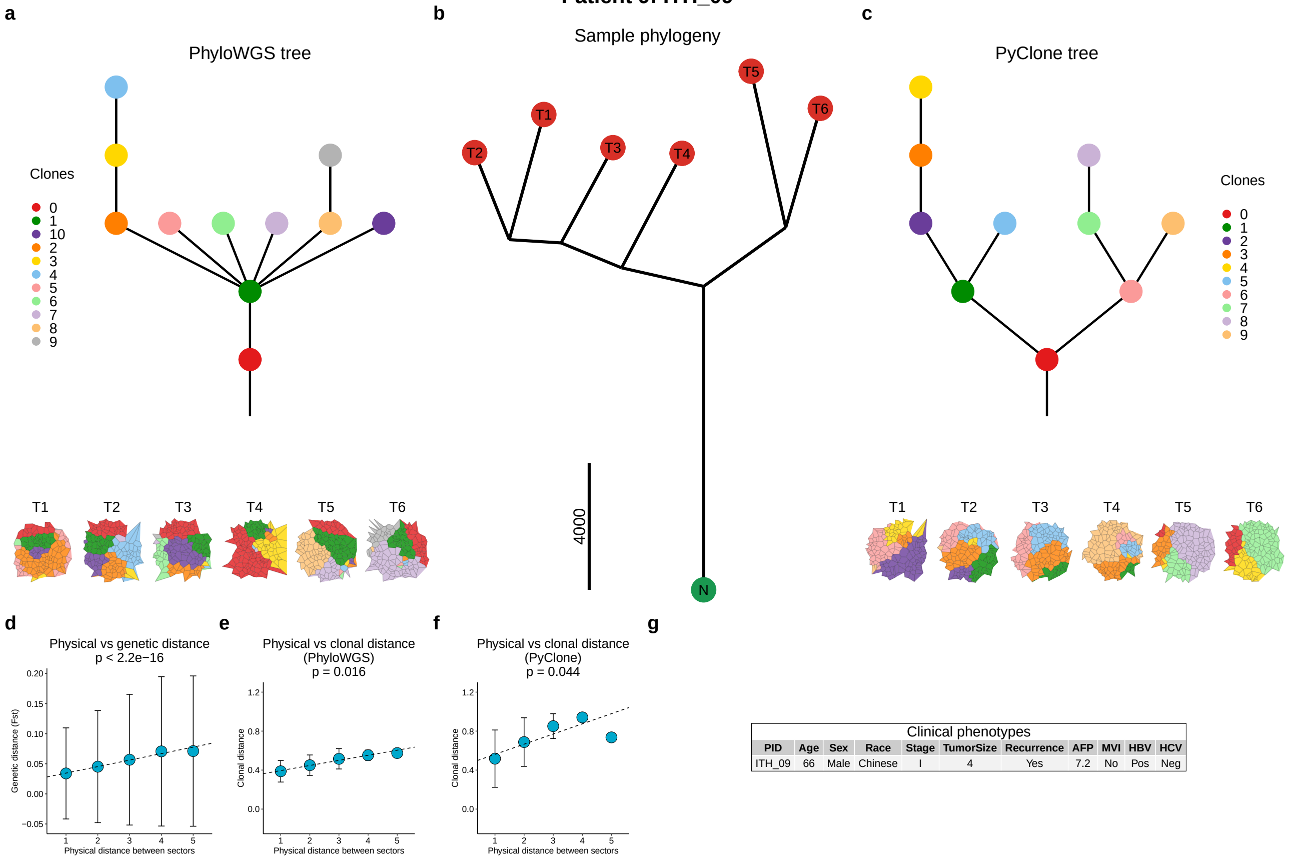

Clinical phenotypes

PID	Age	Sex	Race	Stage	TumorSize	Recurrence	AFP	MVI	HBV	HCV
ITH_09	66	Male	Chinese	I	4	Yes	7.2	No	Pos	Neg

Patient 10: ITH_10

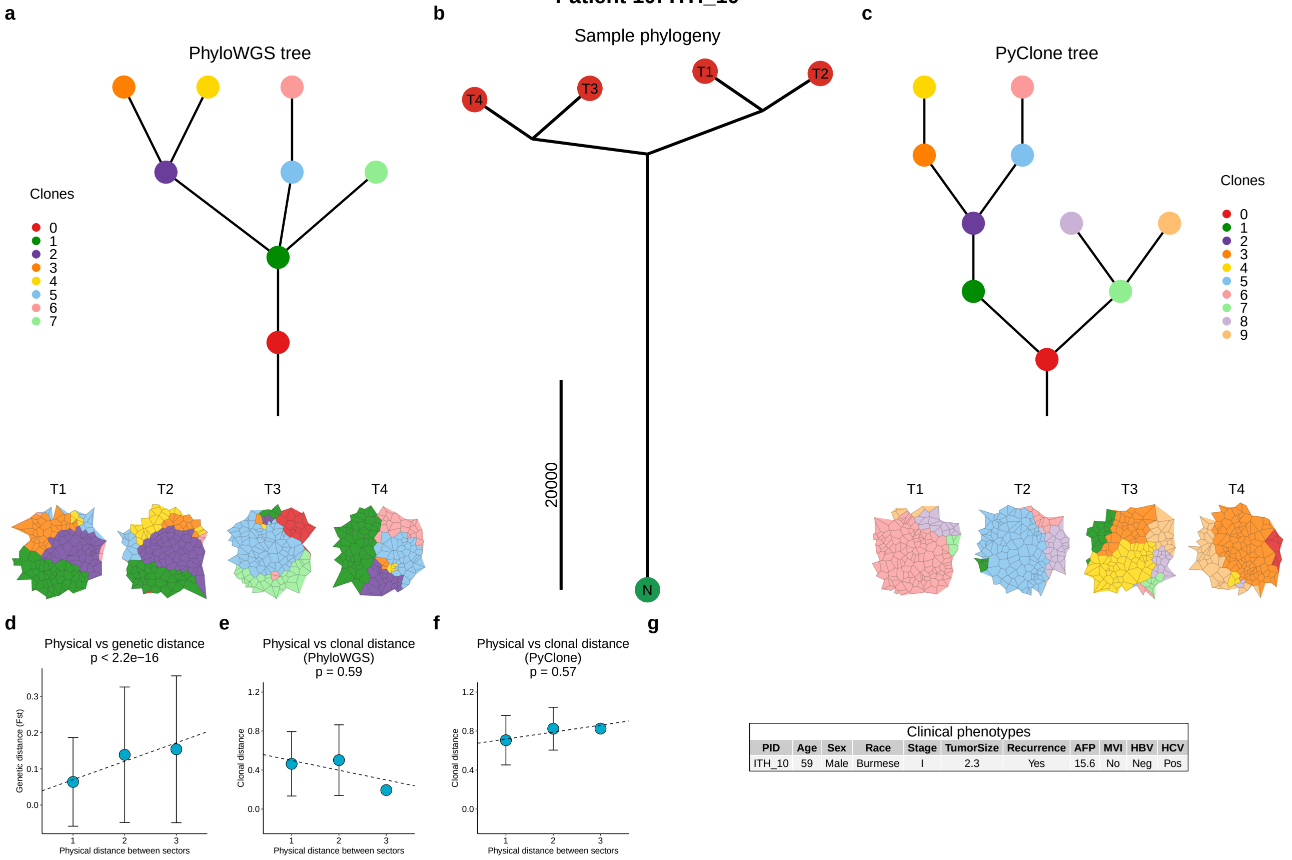

Clinical phenotypes

PID	Age	Sex	Race	Stage	TumorSize	Recurrence	AFP	MVI	HBV	HCV
ITH_10	59	Male	Burmese	I	2.3	Yes	15.6	No	Neg	Pos

Patient 11: ITH_11

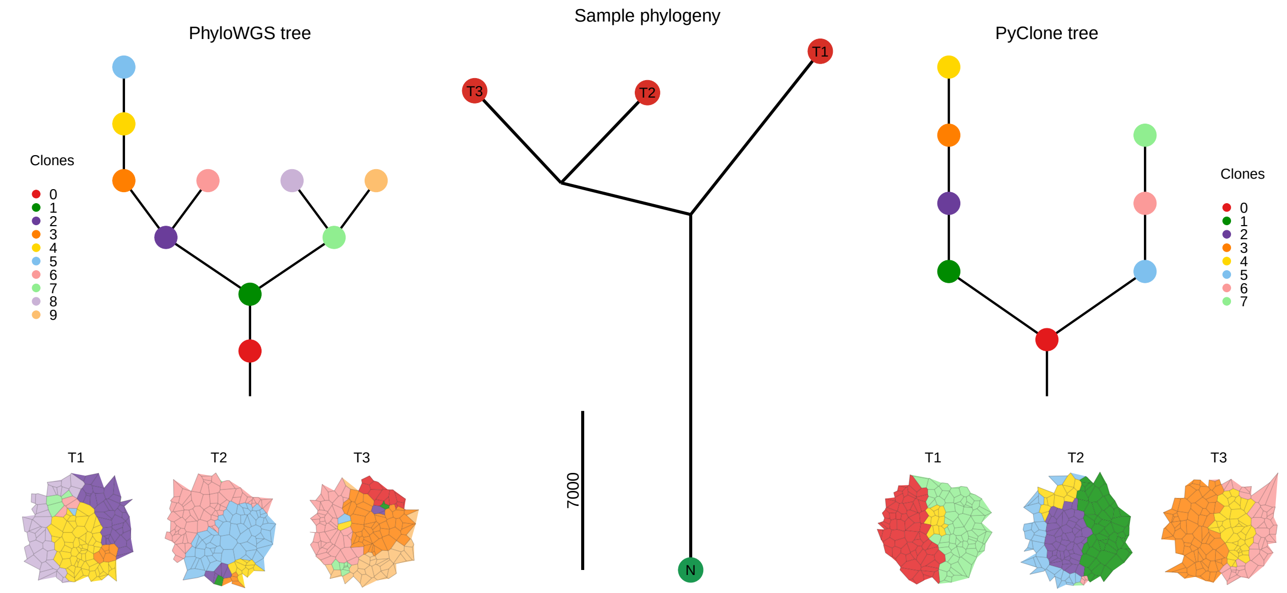

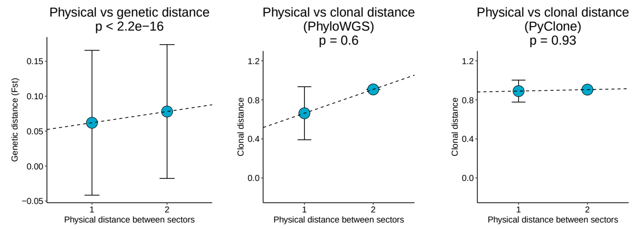

g

Clinical phenotypes

PID	Age	Sex	Race	Stage	TumorSize	Recurrence	AFP	MVI	HBV	HCV
ITH_11	64	Male	Chinese	III	18.5	Yes	4072	Yes	Neg	Neg

Patient 12: ITH_12

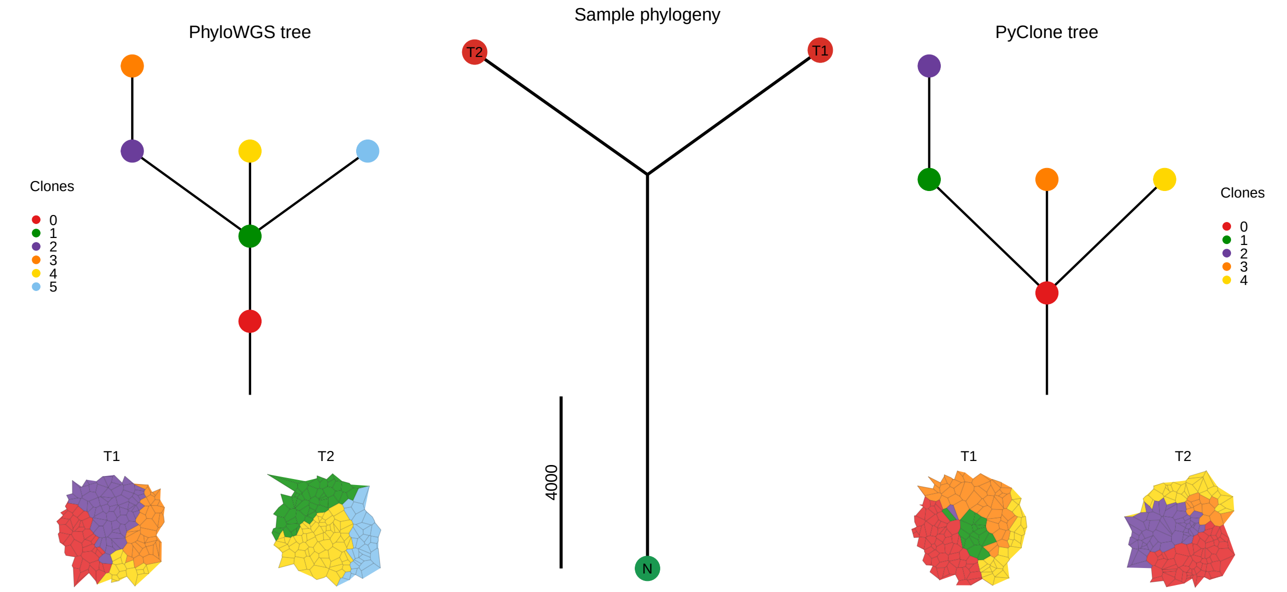

d

This patient does not have
Fst plot (only 2 samples)

e

This patient does not have
Cosine (PhyloWGS) plot (only 2 samples)

f

This patient does not have
Cosine (PyClone) plot (only 2 samples)

g

Clinical phenotypes

PID	Age	Sex	Race	Stage	TumorSize	Recurrence	AFP	MVI	HBV	HCV
ITH_12	59	Male	Chinese	I	1.5	Yes	604	No	Pos	Neg

Patient 13: ITH_13

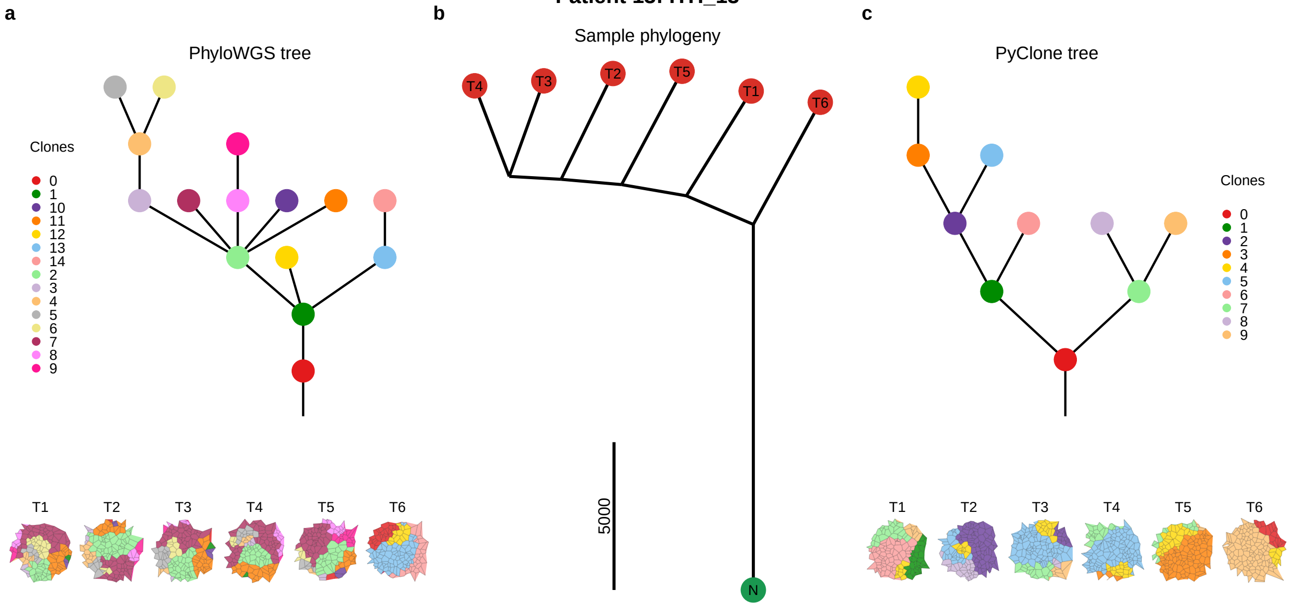

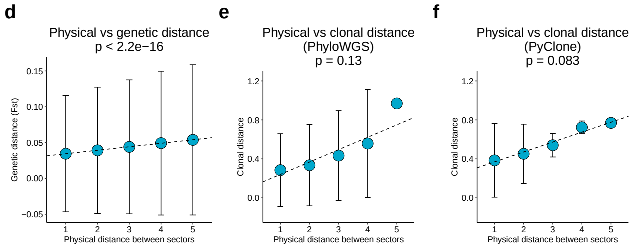

g

Clinical phenotypes

PID	Age	Sex	Race	Stage	TumorSize	Recurrence	AFP	MVI	HBV	HCV
ITH_13	80	Female	Chinese	III	7.9	Yes	2.6	Yes	Neg	Neg

Patient 14: ITH_14

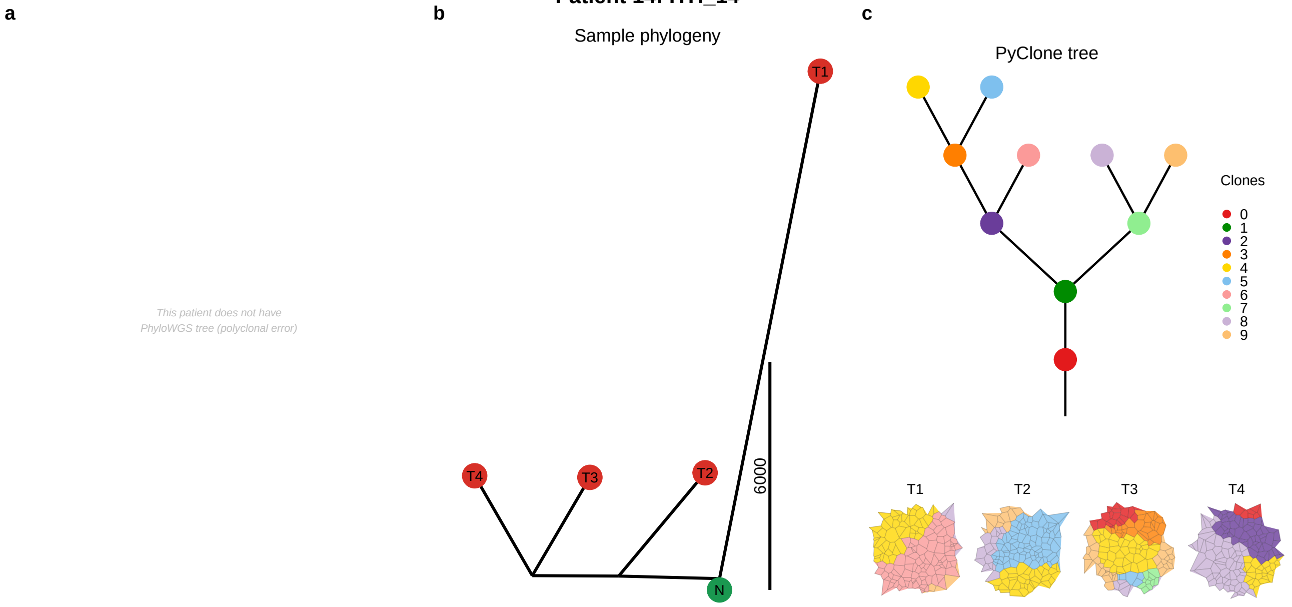

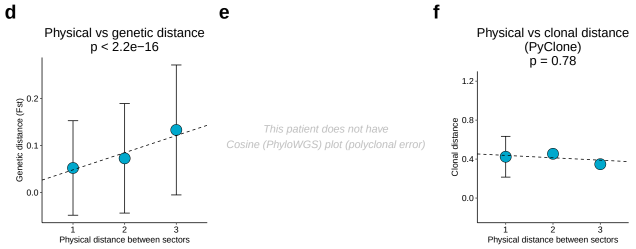

g

Clinical phenotypes

PID	Age	Sex	Race	Stage	TumorSize	Recurrence	AFP	MVI	HBV	HCV
ITH_14	70	Male	Chinese	I	2	No	31.2	No	Pos	Neg

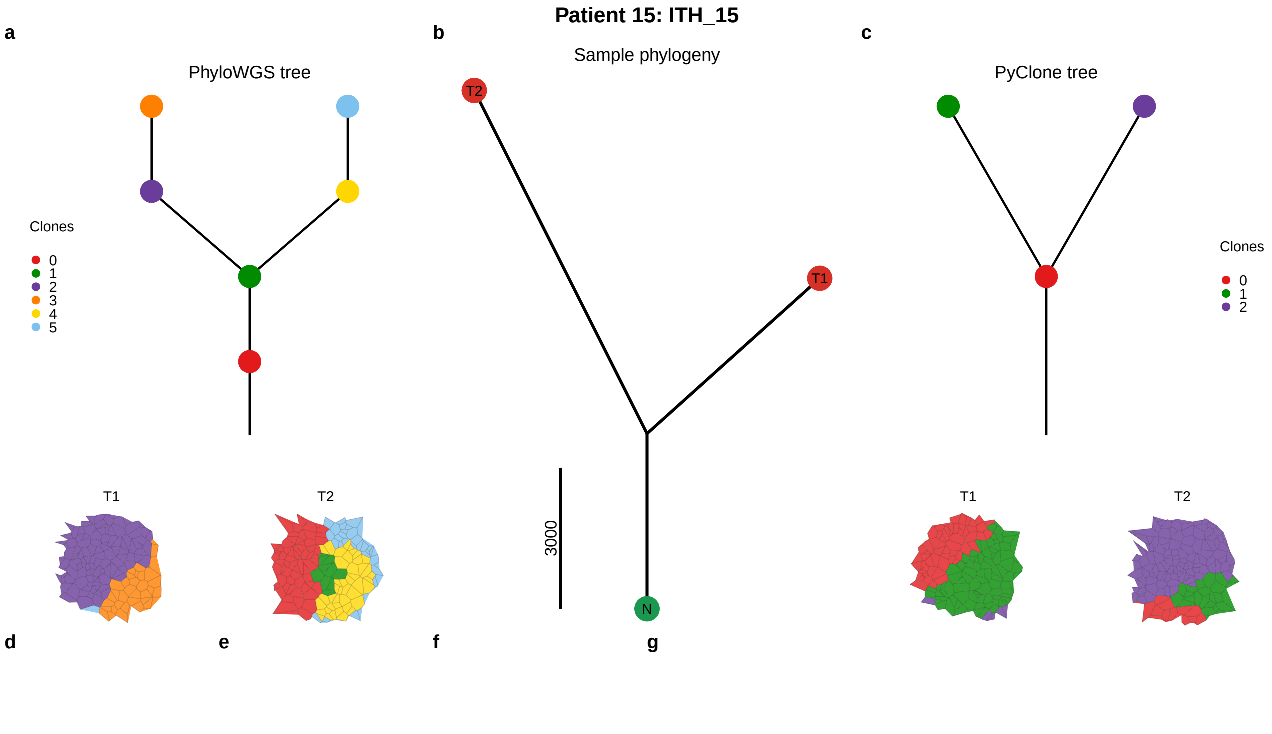

Clinical phenotypes

PID	Age	Sex	Race	Stage	TumorSize	Recurrence	AFP	MVI	HBV	HCV
ITH_15	66	Male	Chinese	I	2	Yes	6.7	No	Neg	Neg

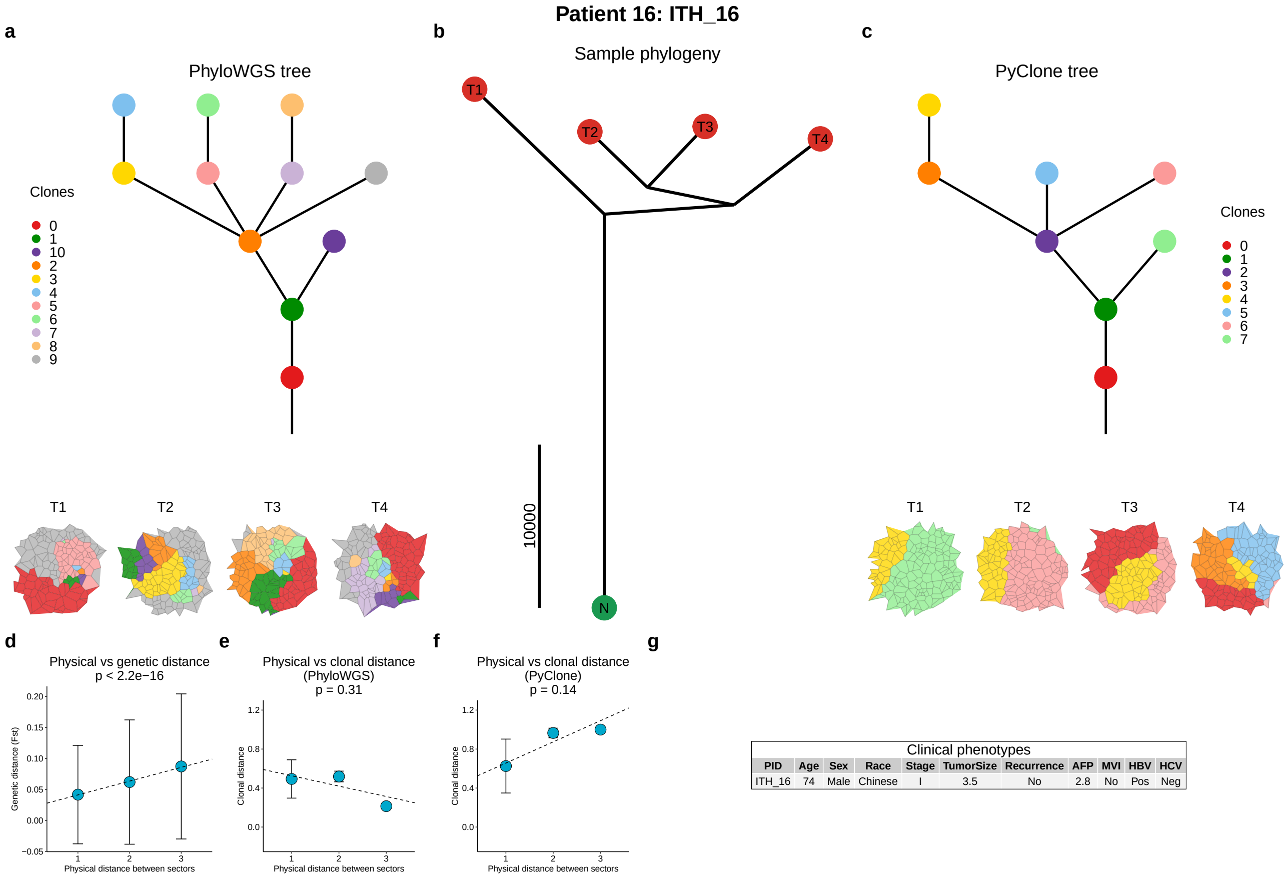

Clinical phenotypes

PID	Age	Sex	Race	Stage	TumorSize	Recurrence	AFP	MVI	HBV	HCV
ITH_16	74	Male	Chinese	I	3.5	No	2.8	No	Pos	Neg

Patient 17: ITH_17

a

b

c

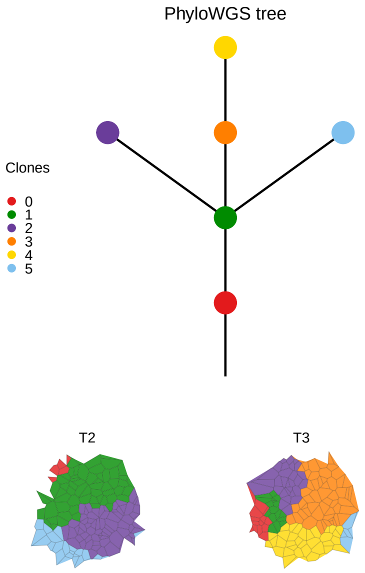

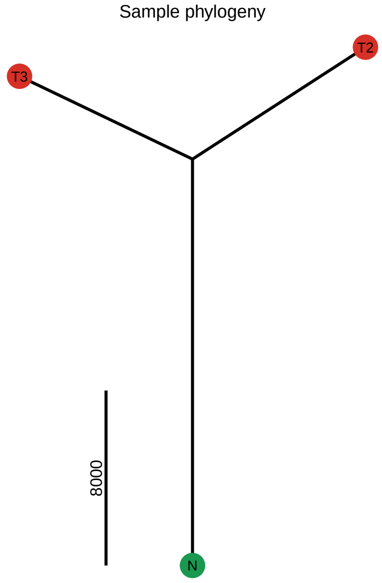

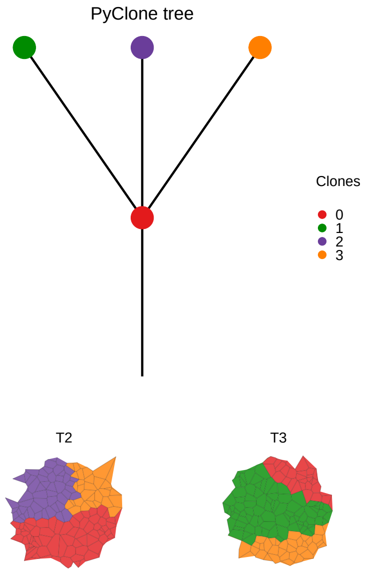

d

This patient does not have Fst plot (only 2 samples)

e

This patient does not have Cosine (PhyloWGS) plot (only 2 samples)

f

This patient does not have Cosine (PyClone) plot (only 2 samples)

g

Clinical phenotypes

PID	Age	Sex	Race	Stage	TumorSize	Recurrence	AFP	MVI	HBV	HCV
ITH_17	77	Male	Chinese	II	3	Yes	38.0	Yes	Pos	Neg

Patient 18: ITH_18

a

b

c

This patient is excluded from DNA analysis due to low purity

d

e

f

g

Clinical phenotypes

PID	Age	Sex	Race	Stage	TumorSize	Recurrence	AFP	MVI	HBV	HCV
ITH_18	49	Male	Chinese	II	5.7	No	15.3	Yes	Pos	Neg

Patient 19: ITH_19

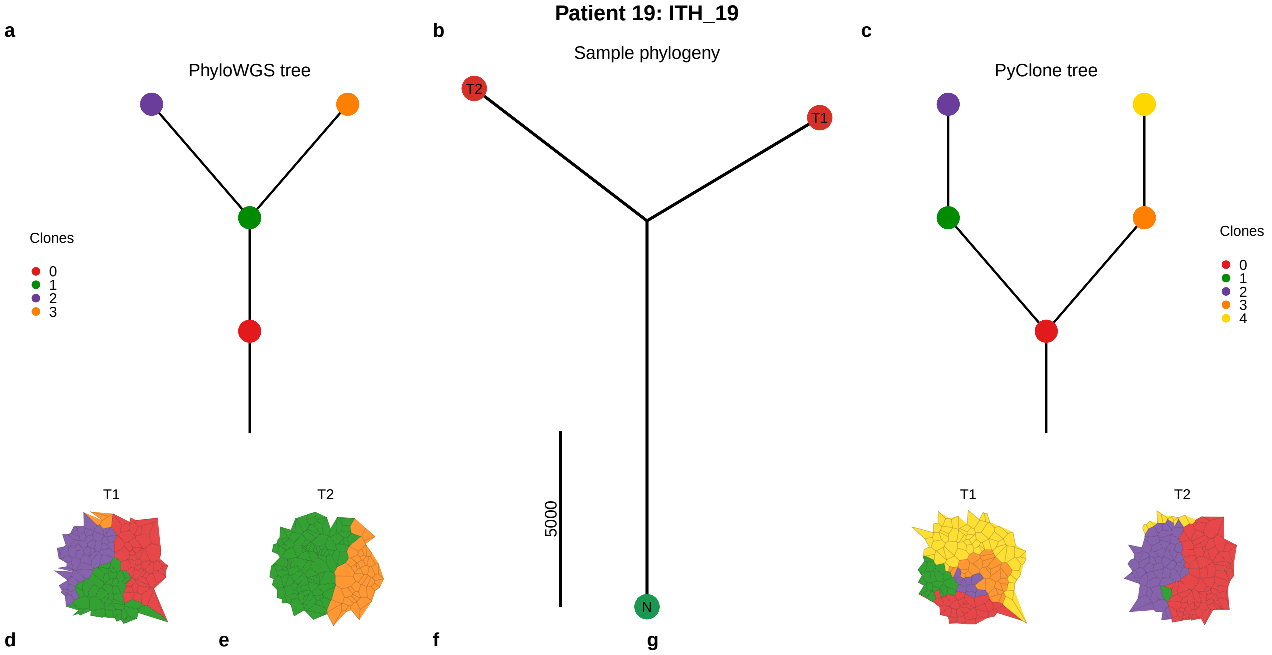

This patient does not have
Fst plot (only 2 samples)

This patient does not have
Cosine (PhyloWGS) plot (only 2 samples)

This patient does not have
Cosine (PyClone) plot (only 2 samples)

Clinical phenotypes

PID	Age	Sex	Race	Stage	TumorSize	Recurrence	AFP	MVI	HBV	HCV
ITH_19	59	Male	Malay	II	2.3	Yes	56.5	Yes	Pos	Neg

Patient 20: ITH_20

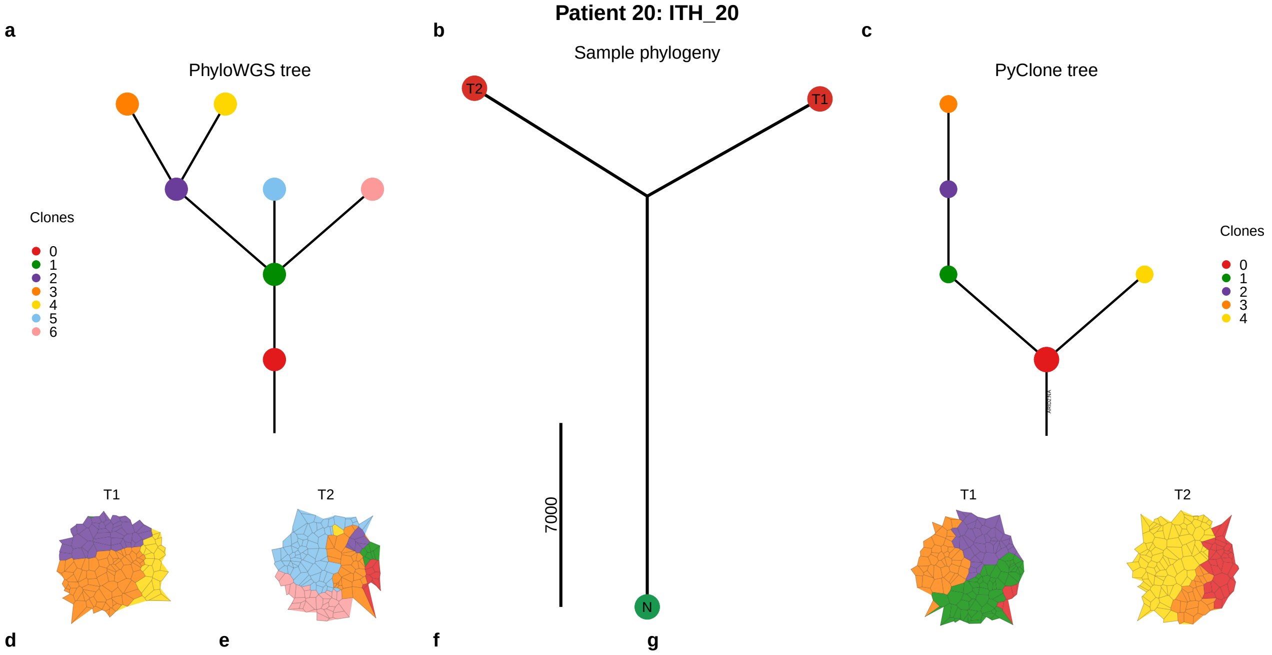

This patient does not have
Fst plot (only 2 samples)

This patient does not have
Cosine (PhyloWGS) plot (only 2 samples)

This patient does not have
Cosine (PyClone) plot (only 2 samples)

Clinical phenotypes

PID	Age	Sex	Race	Stage	TumorSize	Recurrence	AFP	MVI	HBV	HCV
ITH_20	52	Male	Chinese	I	2.5	No	4.7	No	Pos	Neg

Patient 21: ITH_21

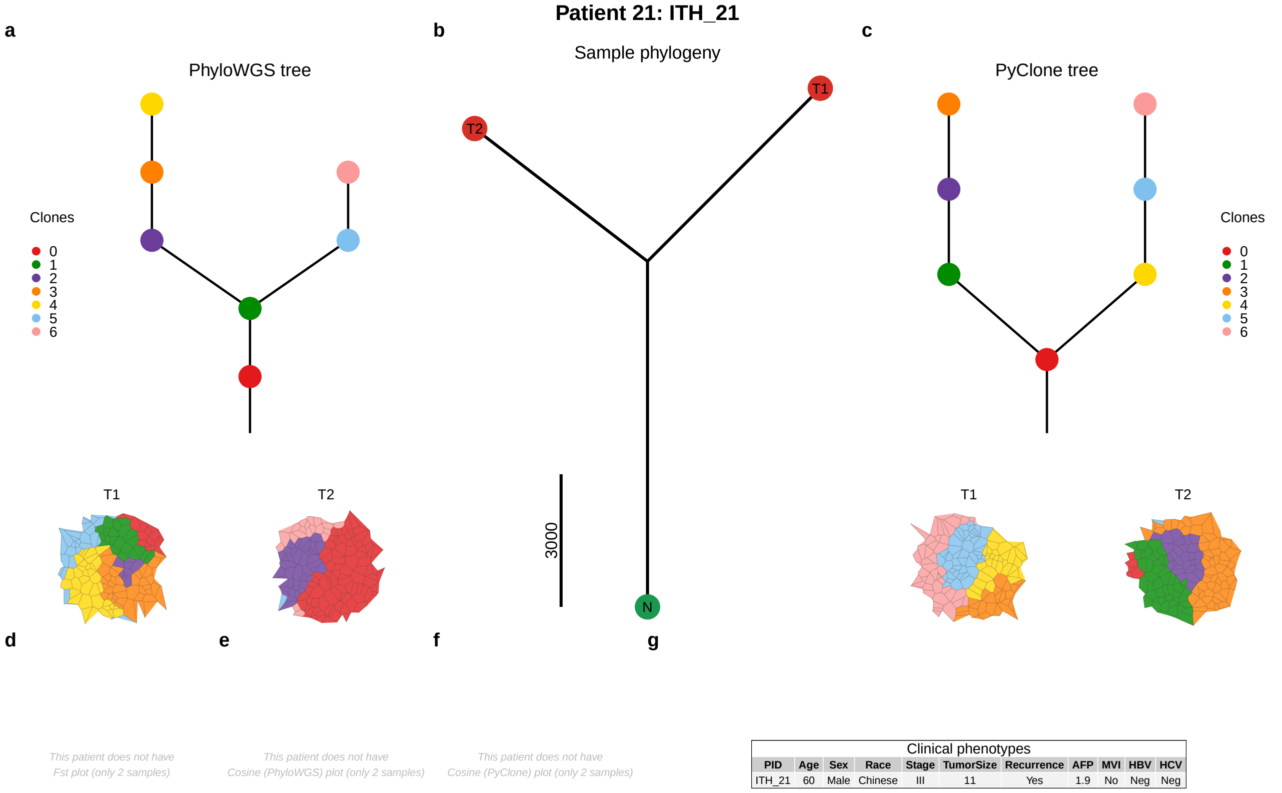

Clinical phenotypes

PID	Age	Sex	Race	Stage	TumorSize	Recurrence	AFP	MVI	HBV	HCV
ITH_21	60	Male	Chinese	III	11	Yes	1.9	No	Neg	Neg

Patient 22: ITH_22

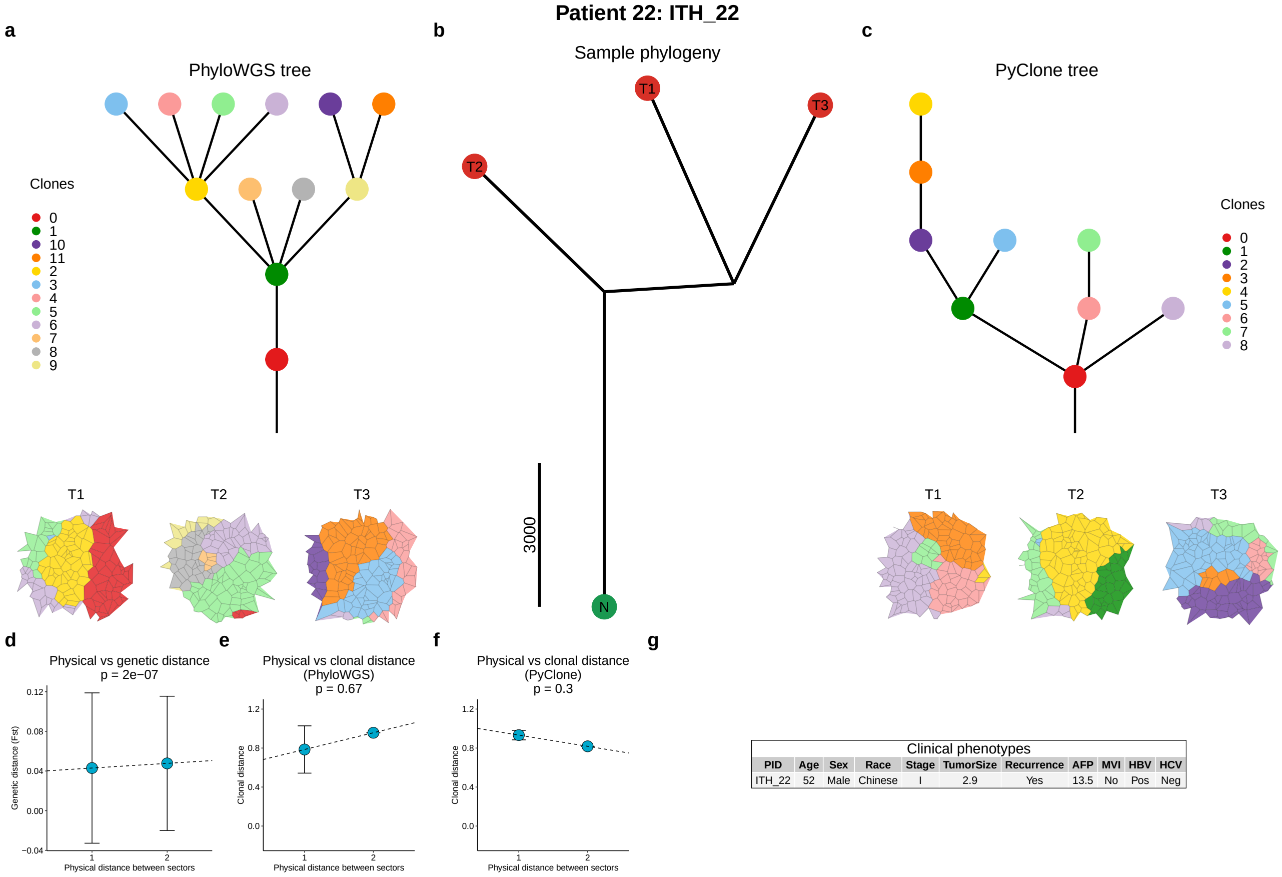

Clinical phenotypes

PID	Age	Sex	Race	Stage	TumorSize	Recurrence	AFP	MVI	HBV	HCV
ITH_22	52	Male	Chinese	I	2.9	Yes	13.5	No	Pos	Neg

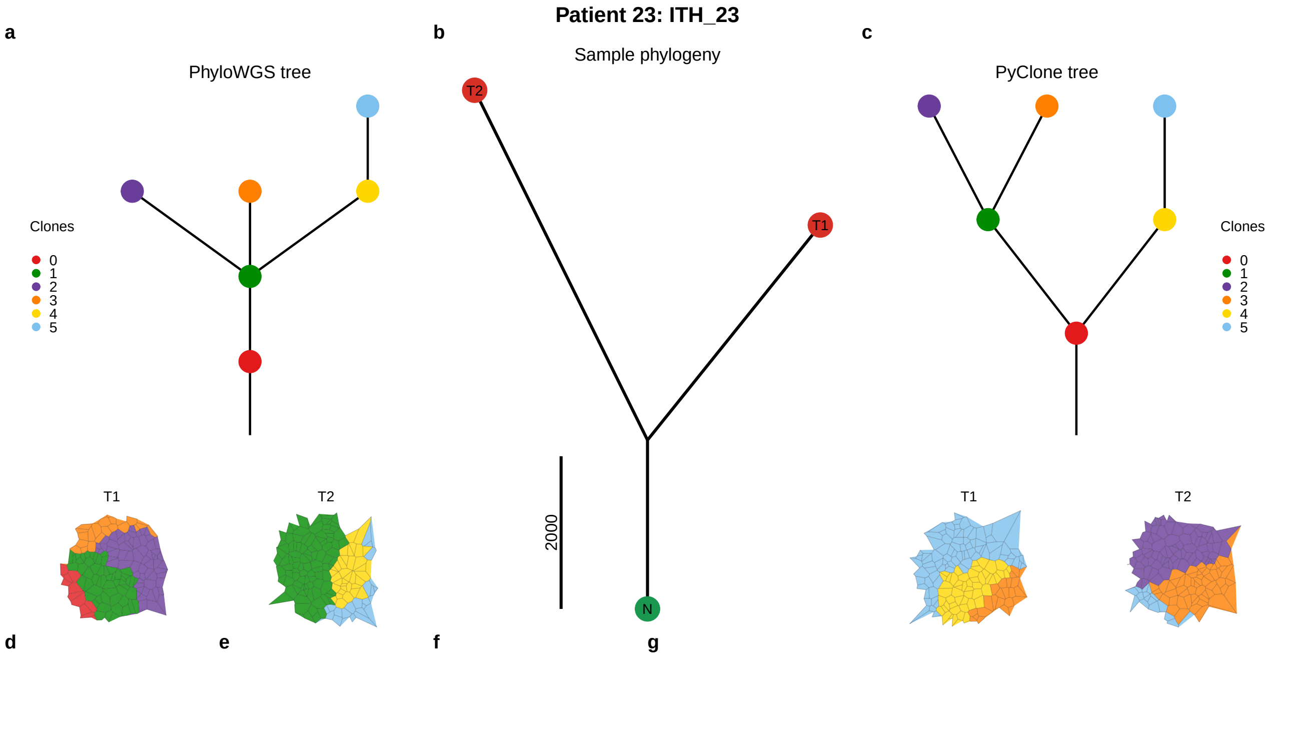

Clinical phenotypes

PID	Age	Sex	Race	Stage	TumorSize	Recurrence	AFP	MVI	HBV	HCV
ITH_23	65	Male	Chinese	I	1.8	No	69.3	No	Pos	Neg

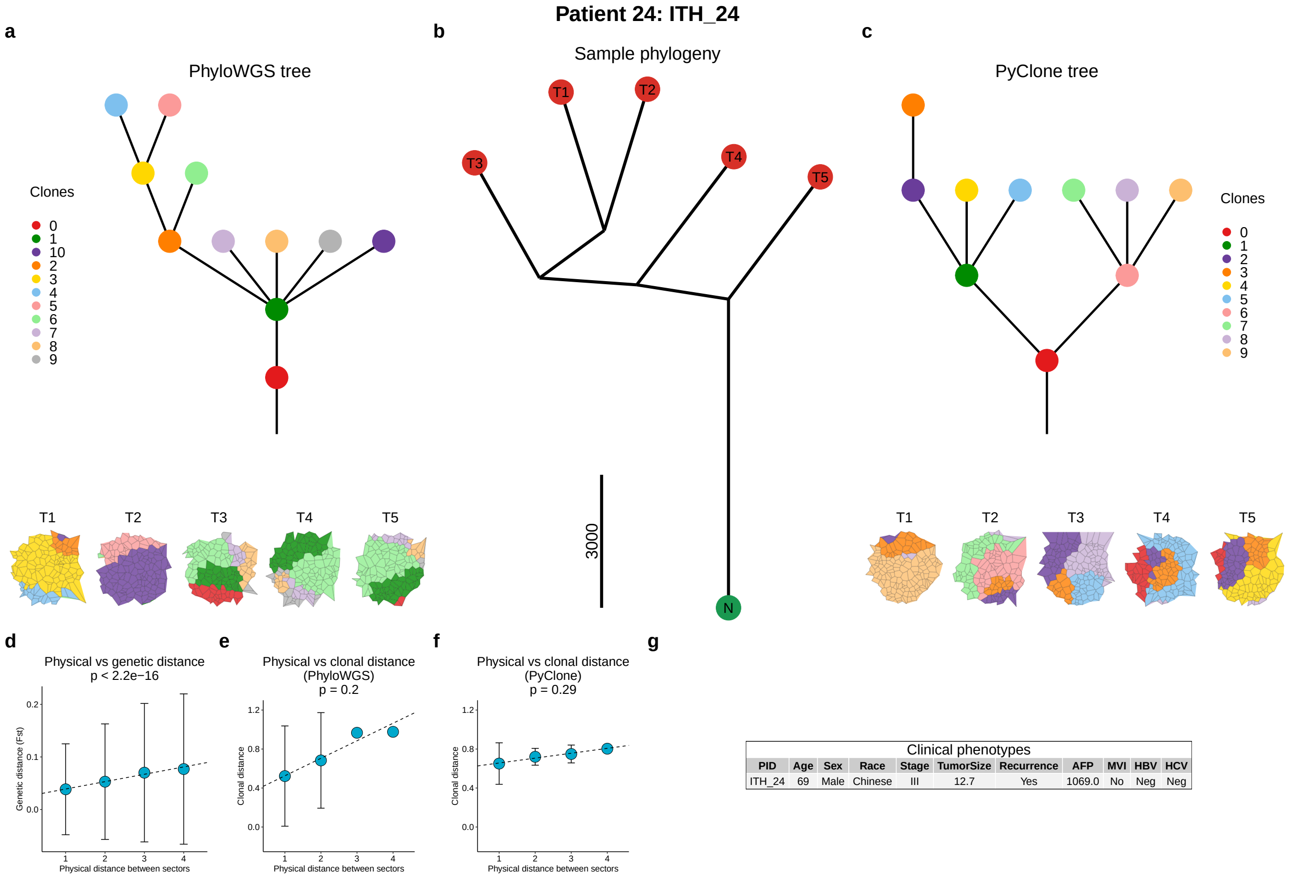

Clinical phenotypes

PID	Age	Sex	Race	Stage	TumorSize	Recurrence	AFP	MVI	HBV	HCV
ITH_24	69	Male	Chinese	III	12.7	Yes	1069.0	No	Neg	Neg

Patient 25: ITH_25

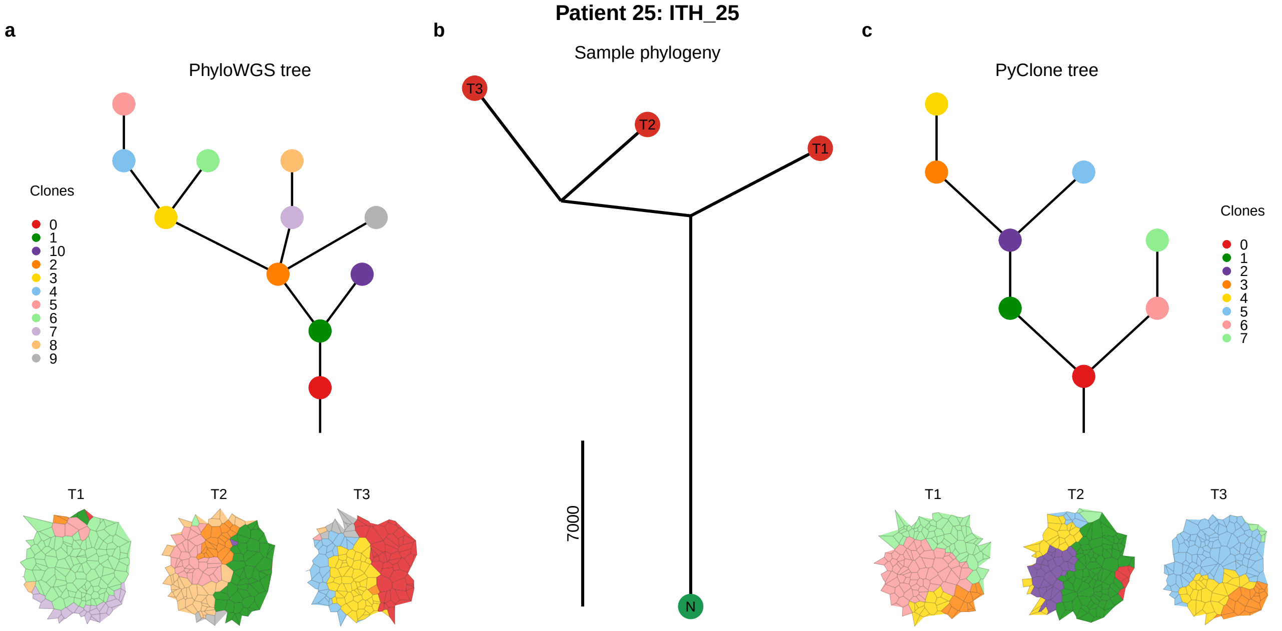

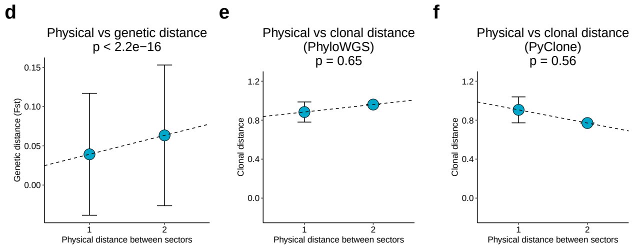

g

Clinical phenotypes

PID	Age	Sex	Race	Stage	TumorSize	Recurrence	AFP	MVI	HBV	HCV
ITH_25	82	Female	Chinese	II	3.5	Yes	27.3	Yes	Neg	Neg

Patient 26: ITH_26

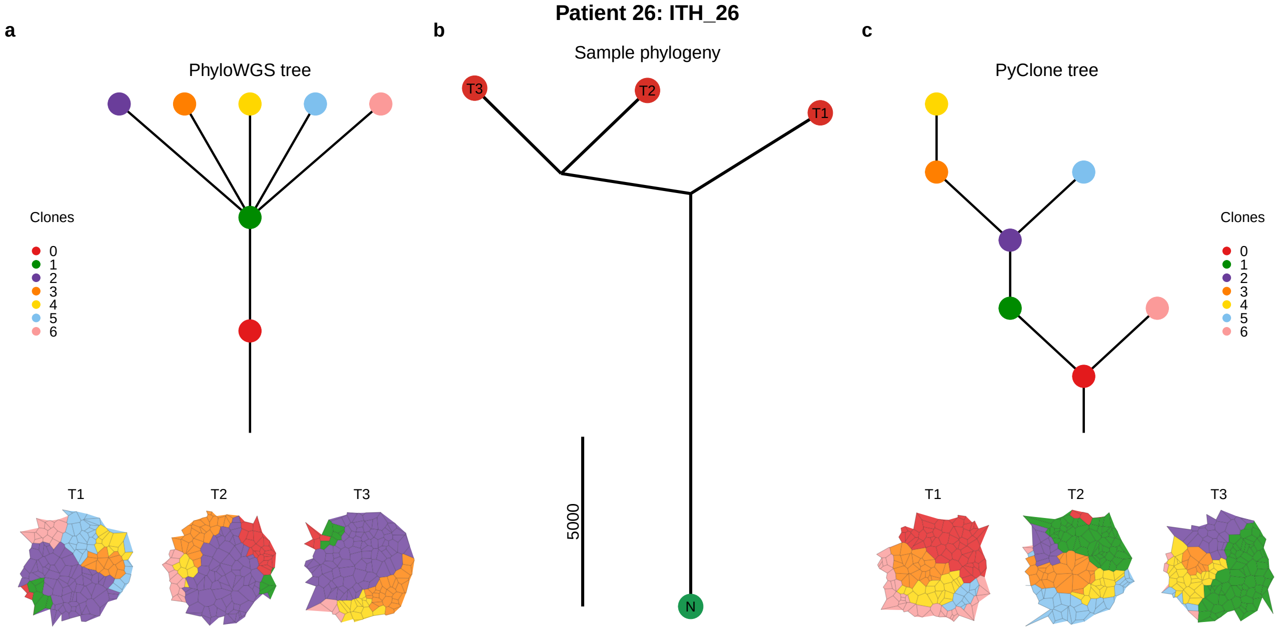

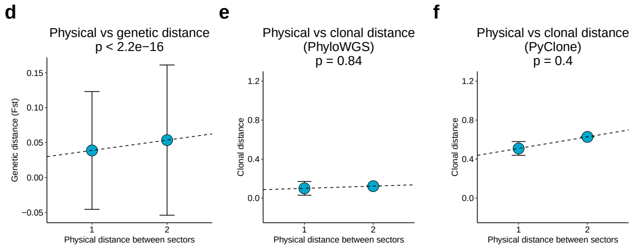

g

Clinical phenotypes

PID	Age	Sex	Race	Stage	TumorSize	Recurrence	AFP	MVI	HBV	HCV
ITH_26	76	Male	Chinese	I	5.5	Yes	3.2	No	Neg	Neg

Patient 27: ITH_27

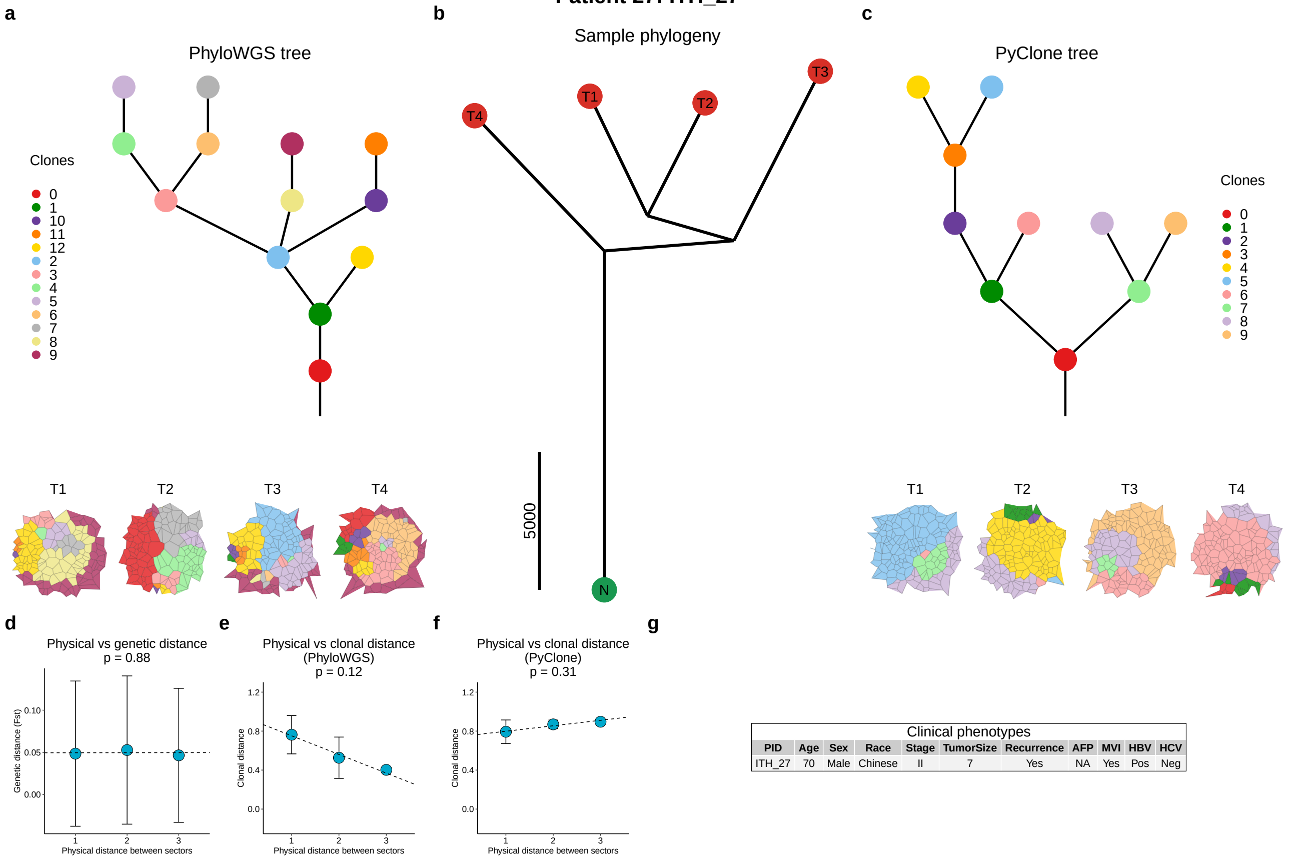

Clinical phenotypes

PID	Age	Sex	Race	Stage	TumorSize	Recurrence	AFP	MVI	HBV	HCV
ITH_27	70	Male	Chinese	II	7	Yes	NA	Yes	Pos	Neg

Patient 28: ITH_28

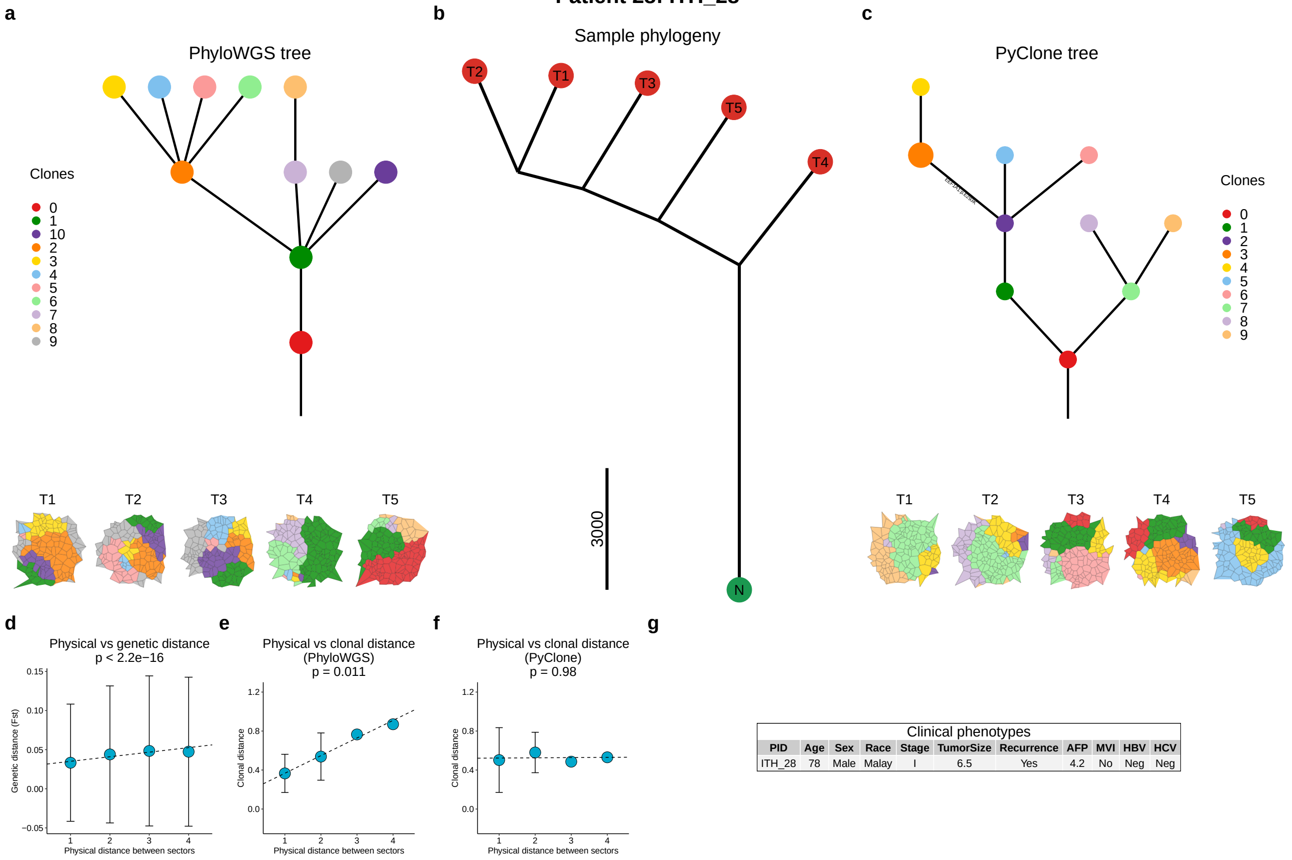

Clinical phenotypes

PID	Age	Sex	Race	Stage	TumorSize	Recurrence	AFP	MVI	HBV	HCV
ITH_28	78	Male	Malay	I	6.5	Yes	4.2	No	Neg	Neg

Patient 29: ITH_29

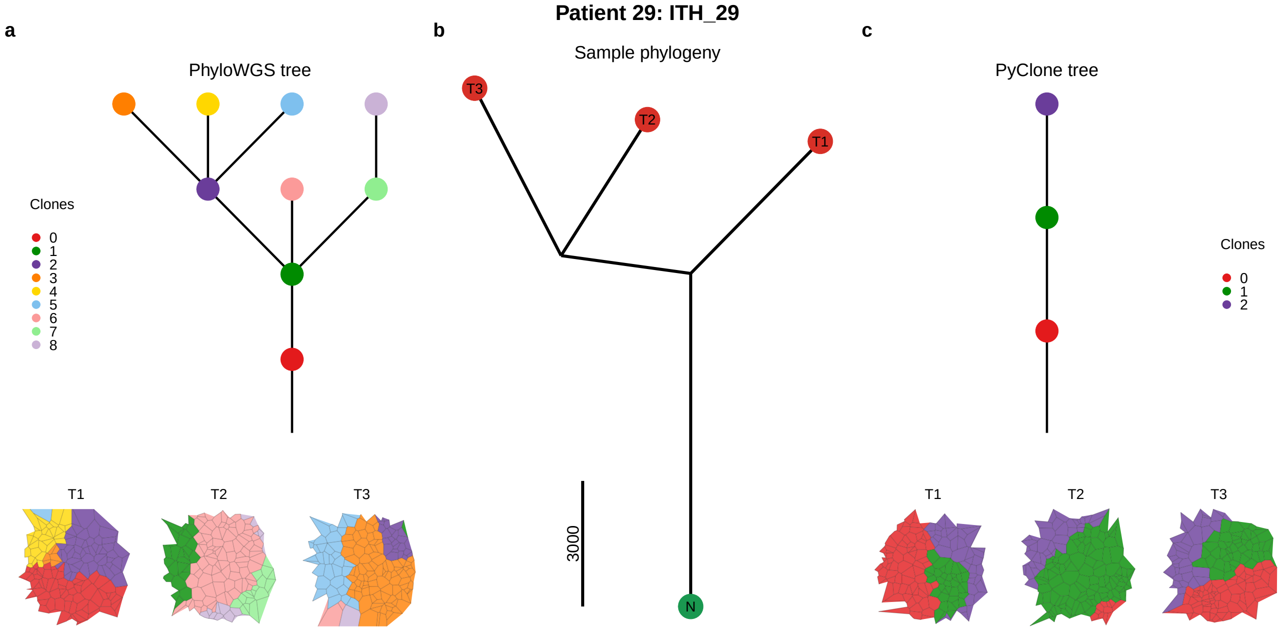

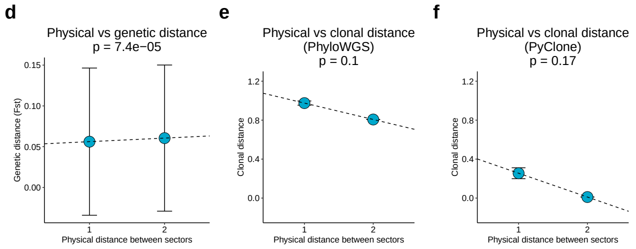

g

Clinical phenotypes

PID	Age	Sex	Race	Stage	TumorSize	Recurrence	AFP	MVI	HBV	HCV
ITH_29	63	Male	Chinese	I	3.5	No	NA	No	Pos	Neg

Patient 30: ITH_30

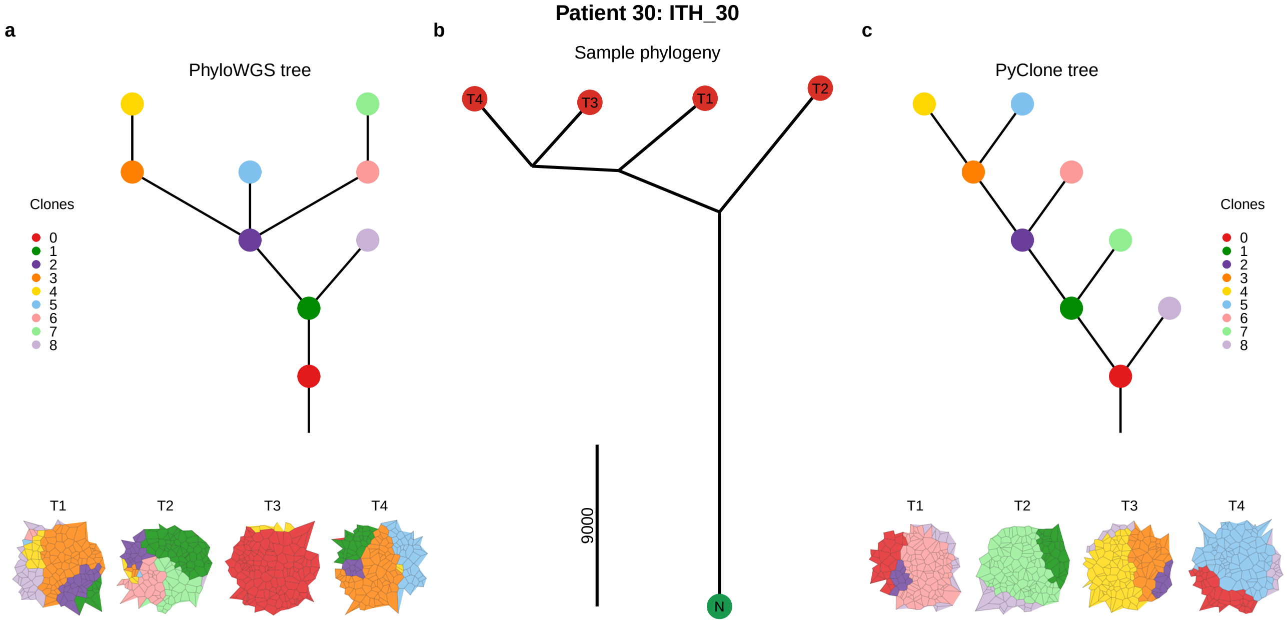

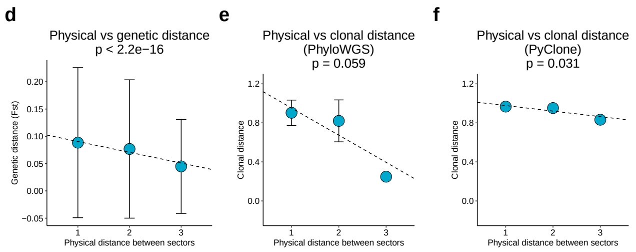

g

Clinical phenotypes

PID	Age	Sex	Race	Stage	TumorSize	Recurrence	AFP	MVI	HBV	HCV
ITH_30	71	Female	Chinese	II	5	Yes	>10000	Yes	Pos	Neg

Patient 31: ITH_31

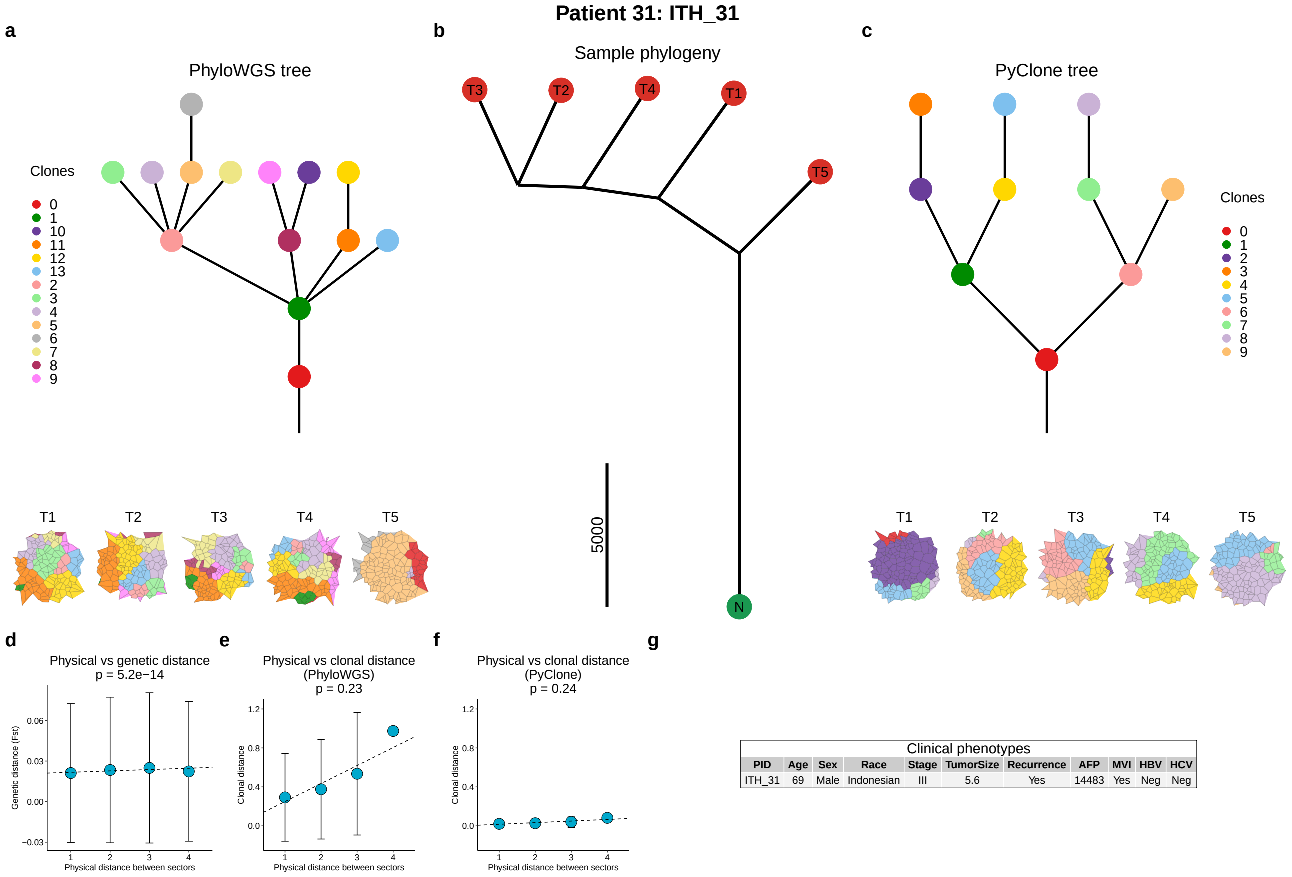

Clinical phenotypes

PID	Age	Sex	Race	Stage	TumorSize	Recurrence	AFP	MVI	HBV	HCV
ITH_31	69	Male	Indonesian	III	5.6	Yes	14483	Yes	Neg	Neg

Patient 32: ITH_32

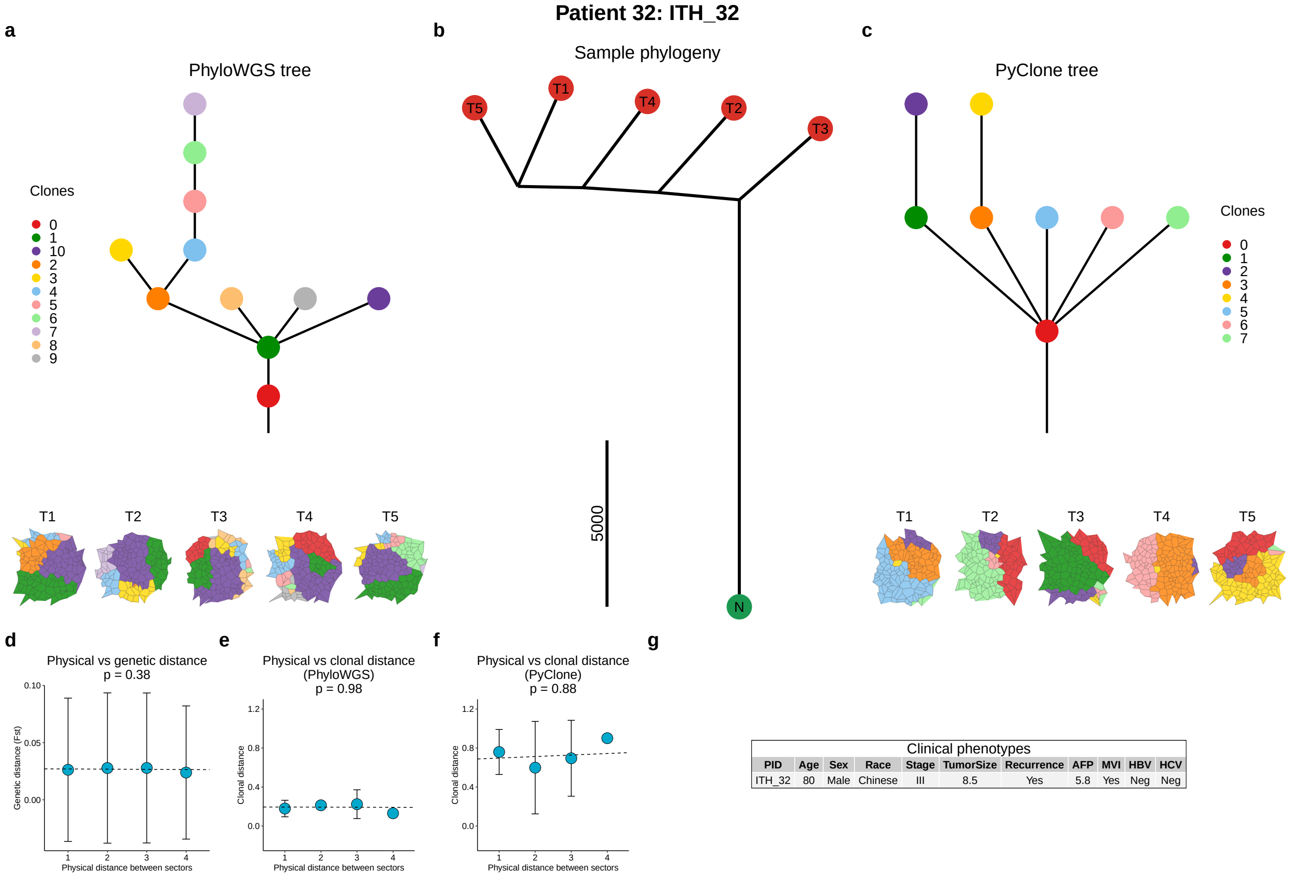

Clinical phenotypes

PID	Age	Sex	Race	Stage	TumorSize	Recurrence	AFP	MVI	HBV	HCV
ITH_32	80	Male	Chinese	III	8.5	Yes	5.8	Yes	Neg	Neg

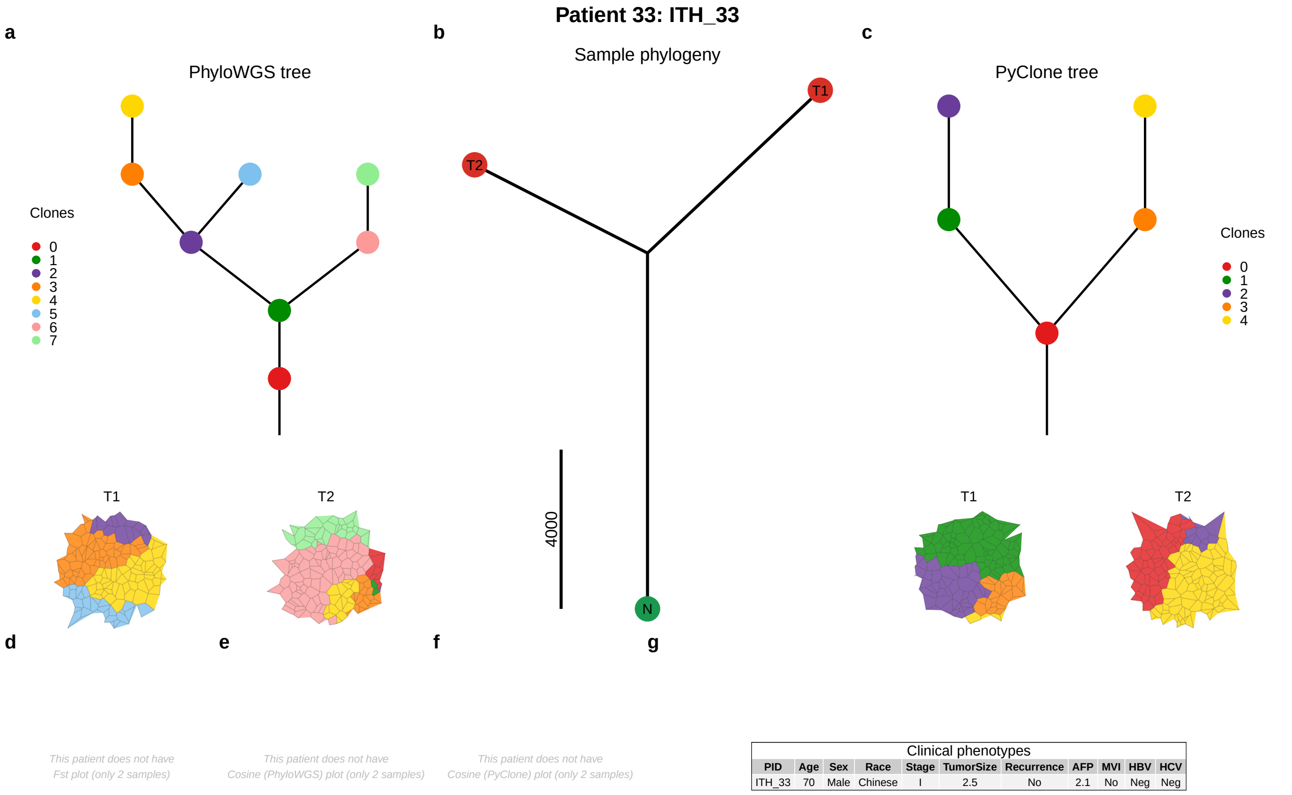

PID	Age	Sex	Race	Stage	TumorSize	Recurrence	AFP	MVI	HBV	HCV
ITH_33	70	Male	Chinese	I	2.5	No	2.1	No	Neg	Neg

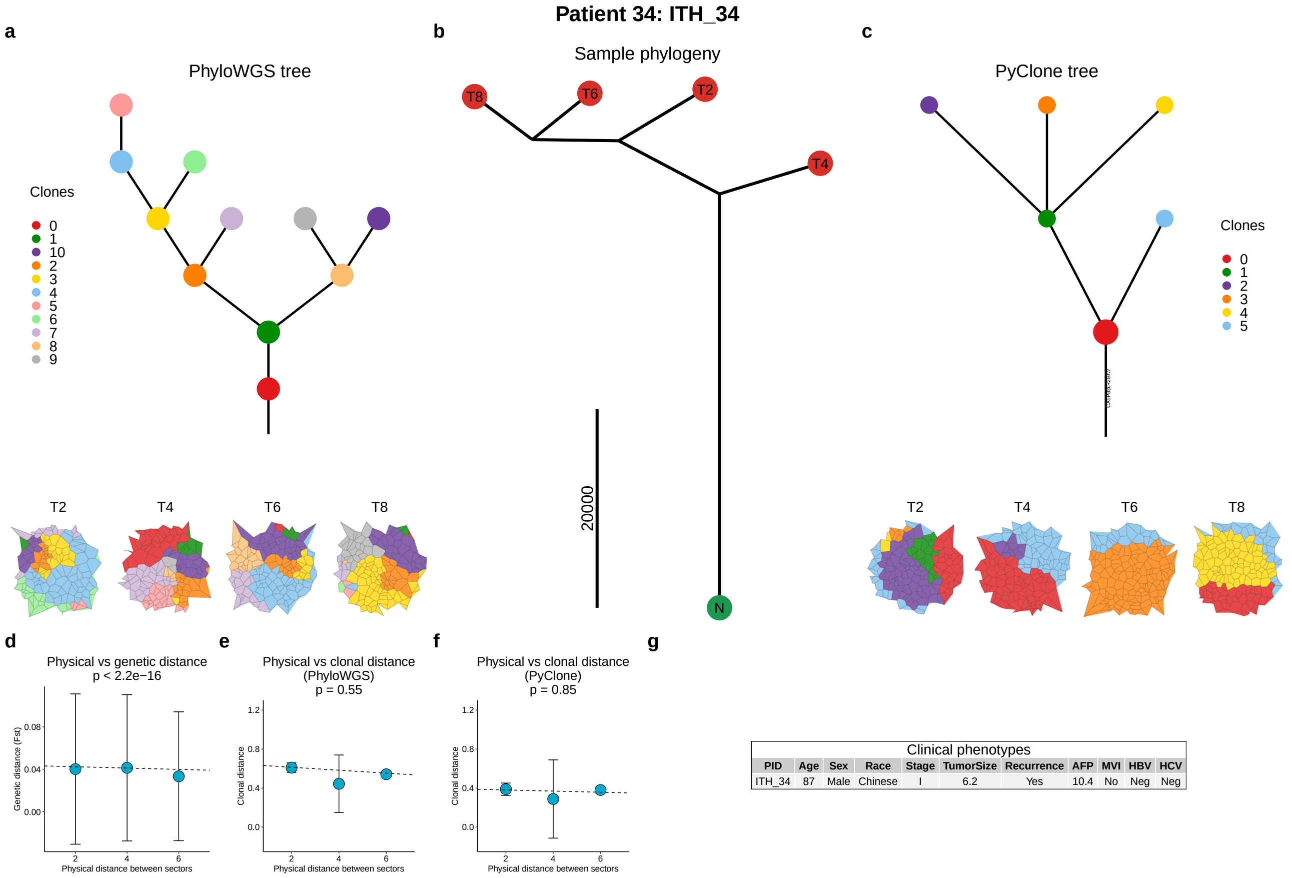

PID	Age	Sex	Race	Stage	TumorSize	Recurrence	AFP	MVI	HBV	HCV
ITH_34	87	Male	Chinese	I	6.2	Yes	10.4	No	Neg	Neg

Patient 35: ITH_35

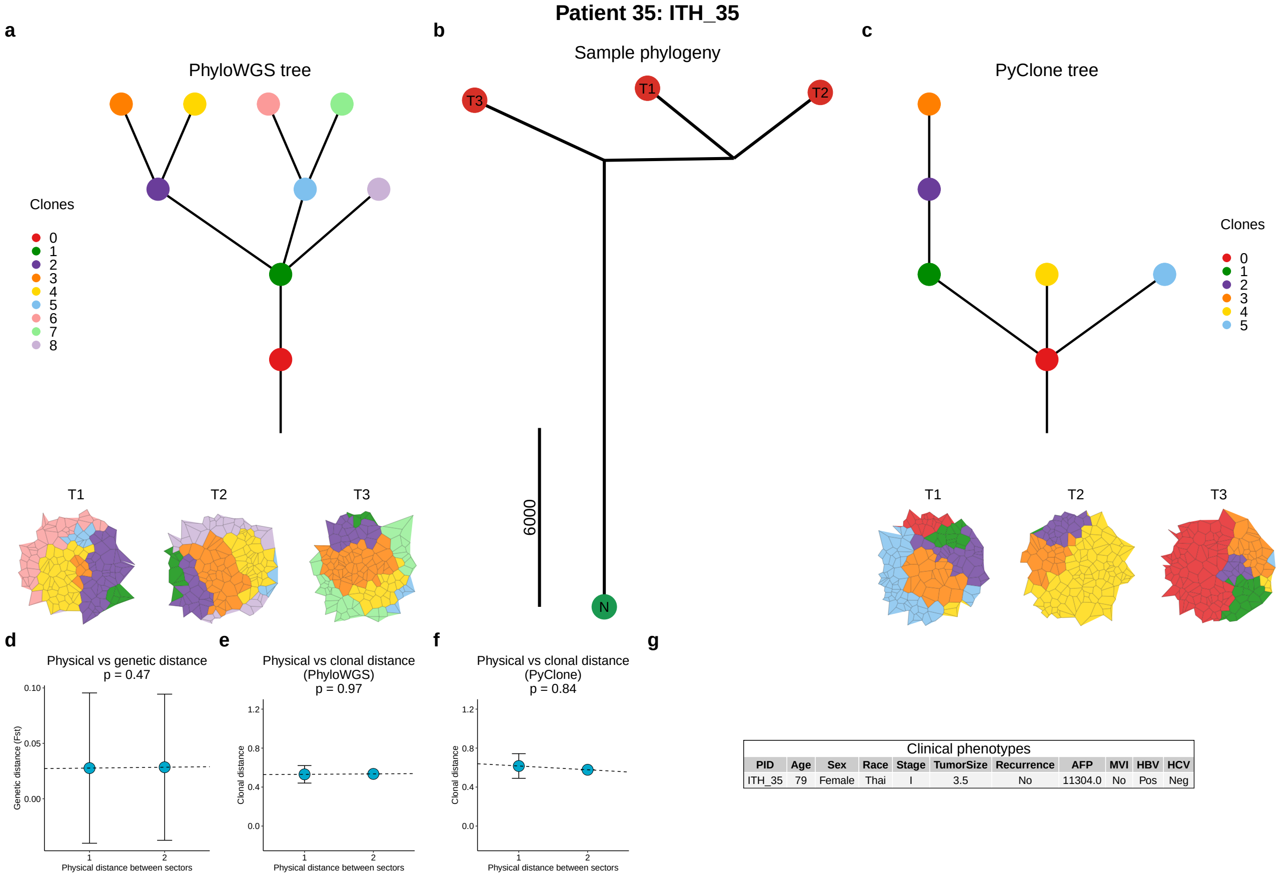

Clinical phenotypes

PID	Age	Sex	Race	Stage	TumorSize	Recurrence	AFP	MVI	HBV	HCV
ITH_35	79	Female	Thai	I	3.5	No	11304.0	No	Pos	Neg

Patient 36: ITH_36

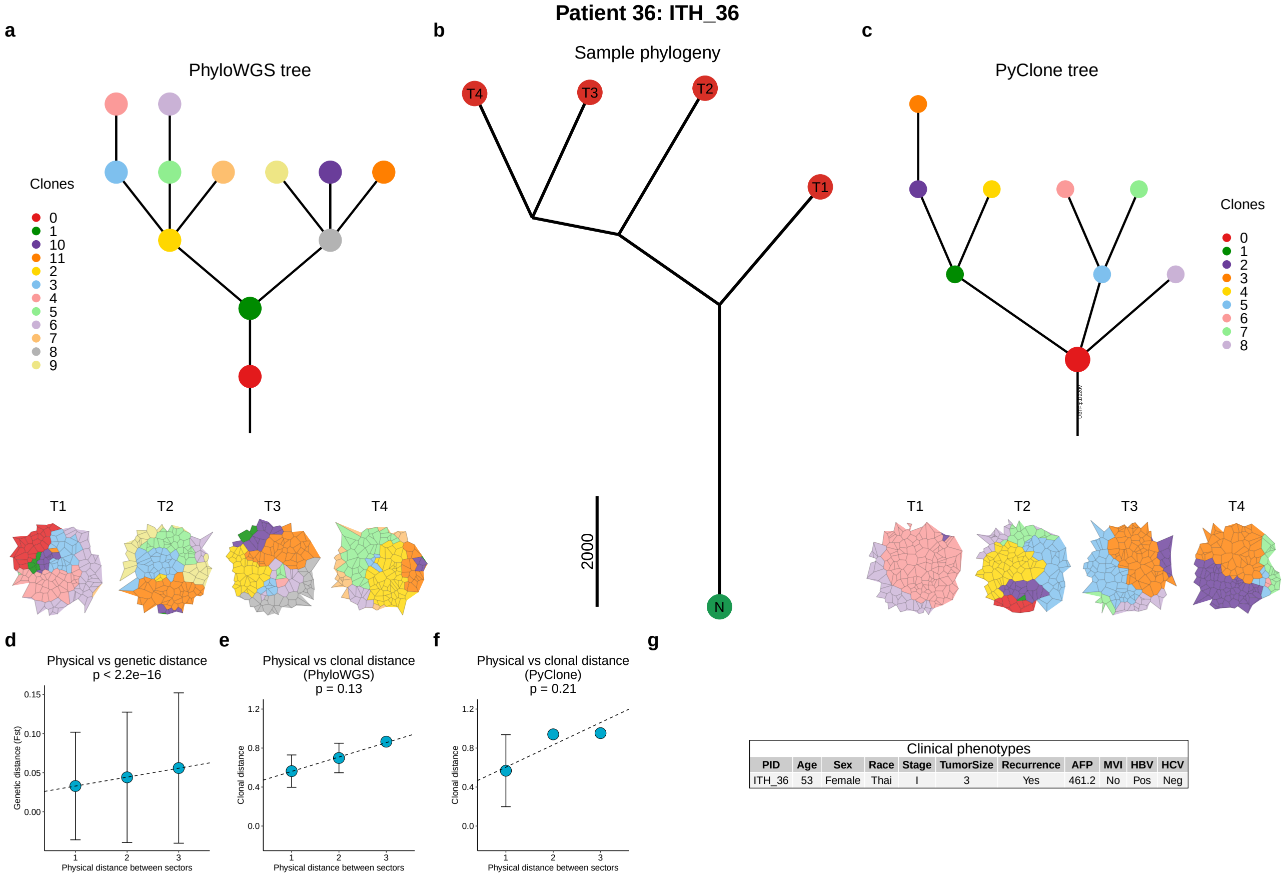

Clinical phenotypes

PID	Age	Sex	Race	Stage	TumorSize	Recurrence	AFP	MVI	HBV	HCV
ITH_36	53	Female	Thai	I	3	Yes	461.2	No	Pos	Neg

Patient 37: ITH_37

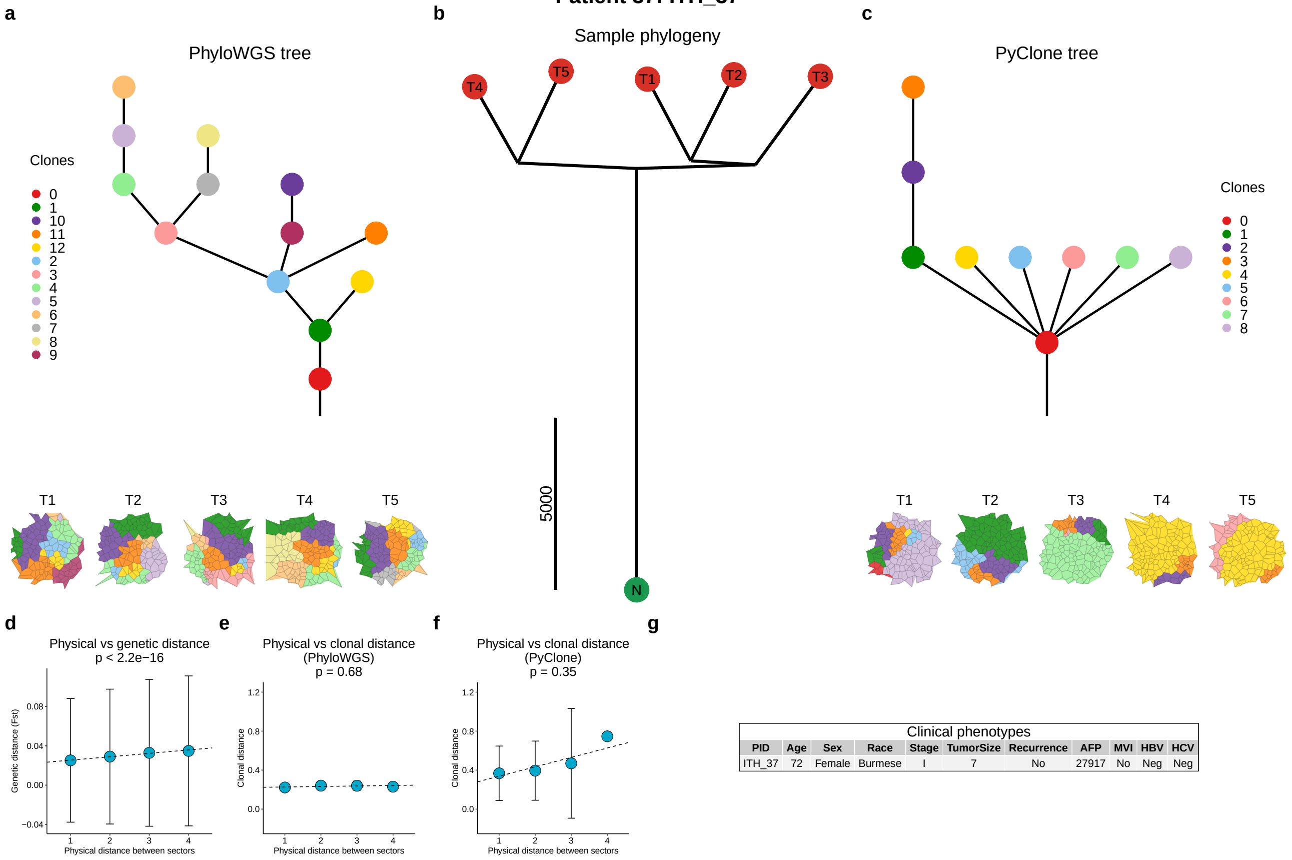

Clinical phenotypes

PID	Age	Sex	Race	Stage	TumorSize	Recurrence	AFP	MVI	HBV	HCV
ITH_37	72	Female	Burmese	I	7	No	27917	No	Neg	Neg

Patient 38: ITH_38

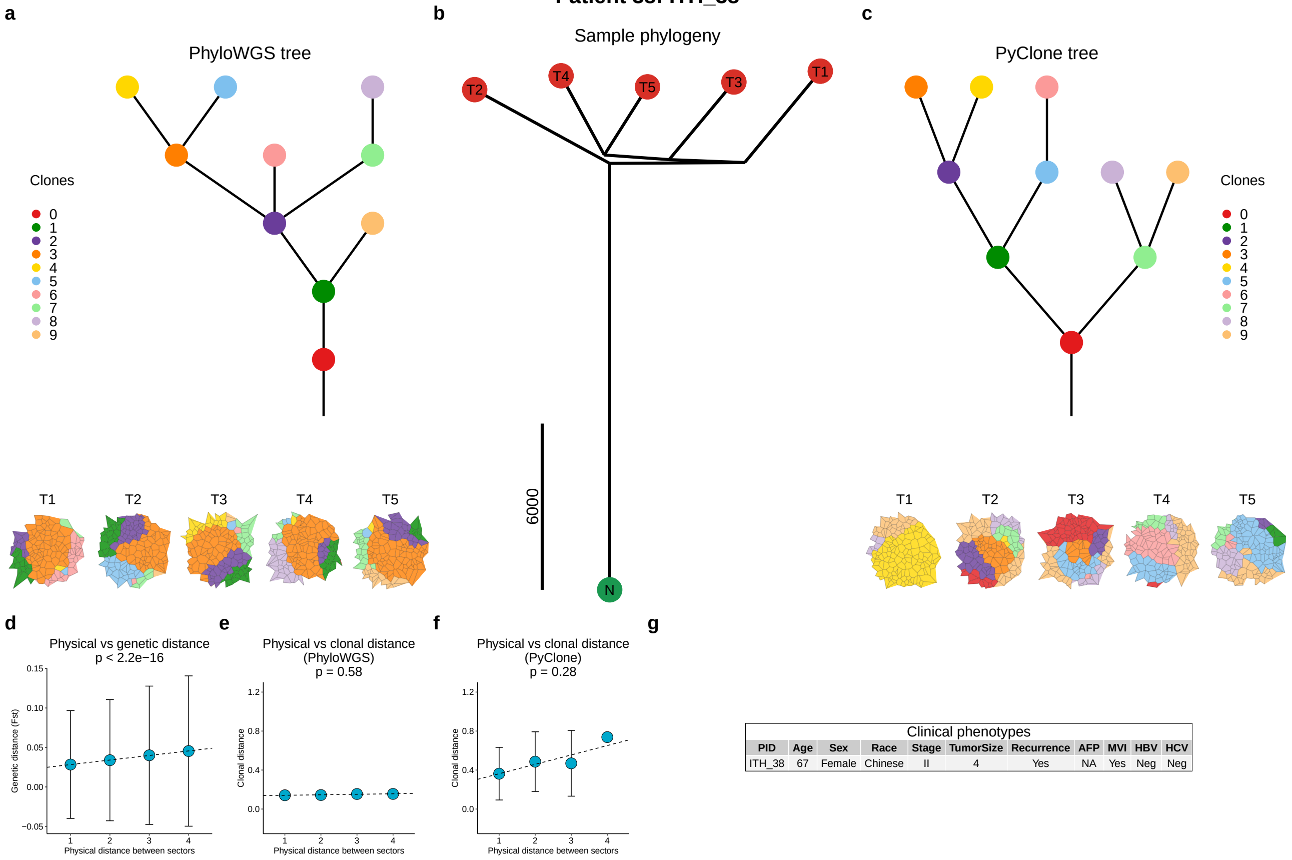

Clinical phenotypes

PID	Age	Sex	Race	Stage	TumorSize	Recurrence	AFP	MVI	HBV	HCV
ITH_38	67	Female	Chinese	II	4	Yes	NA	Yes	Neg	Neg

Patient 39: ITH_39

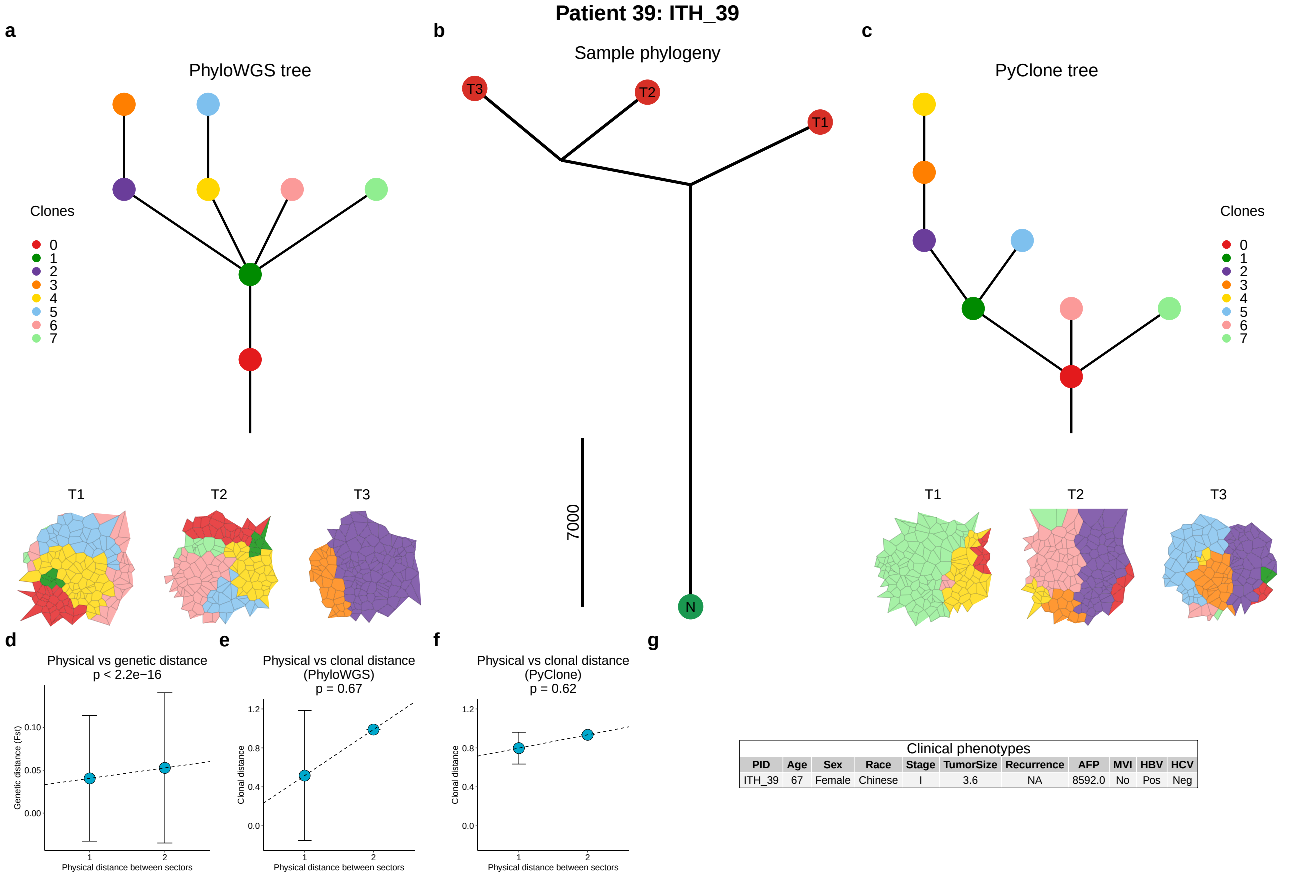

Clinical phenotypes

PID	Age	Sex	Race	Stage	TumorSize	Recurrence	AFP	MVI	HBV	HCV
ITH_39	67	Female	Chinese	I	3.6	NA	8592.0	No	Pos	Neg

Patient 40: ITH_40

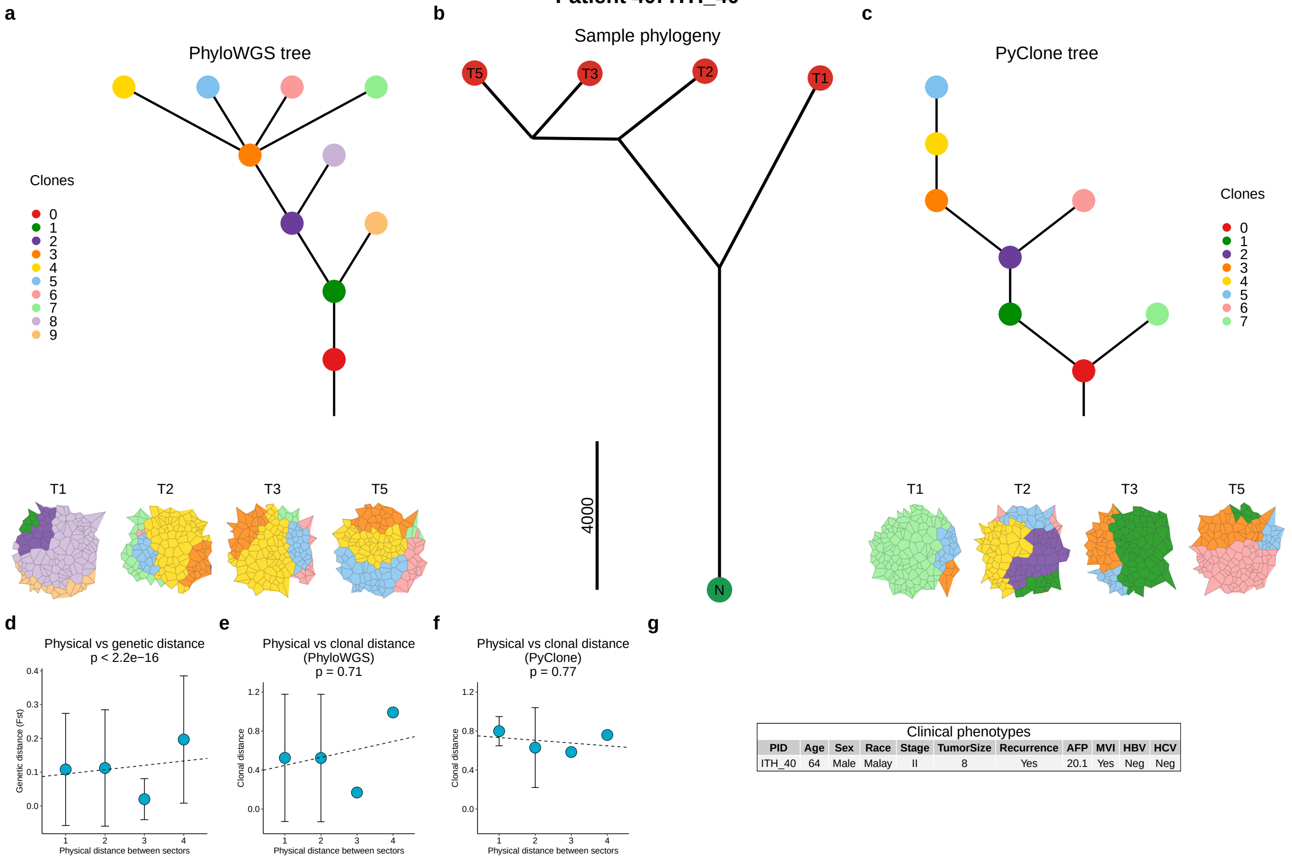

Clinical phenotypes

PID	Age	Sex	Race	Stage	TumorSize	Recurrence	AFP	MVI	HBV	HCV
ITH_40	64	Male	Malay	II	8	Yes	20.1	Yes	Neg	Neg

Patient 41: ITH_41

a

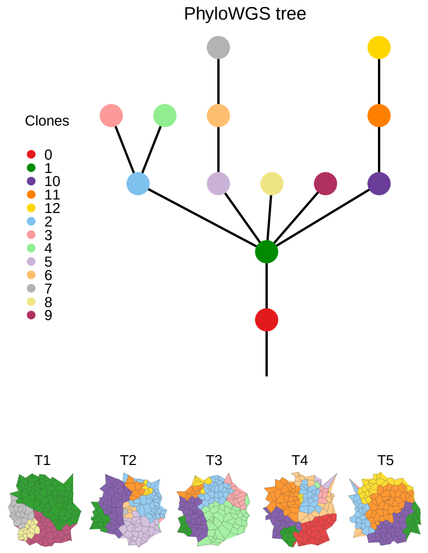

b

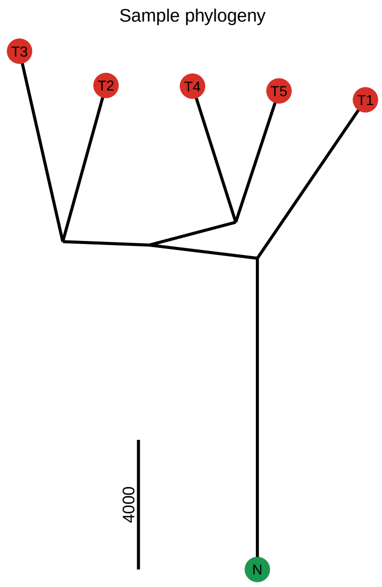

c

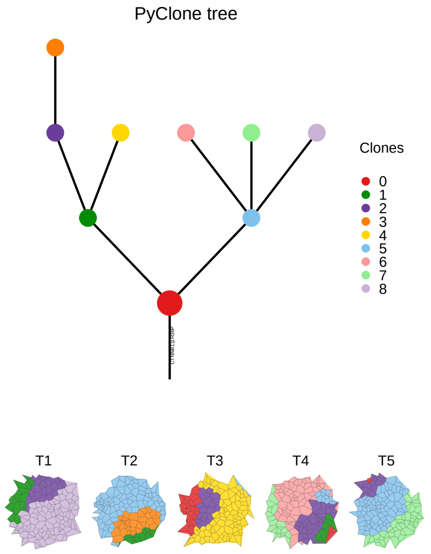

d

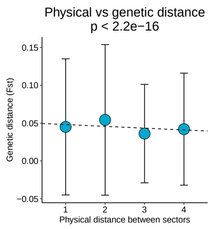

e

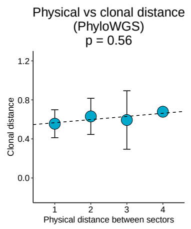

f

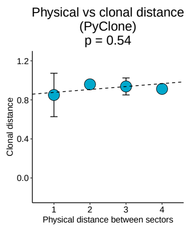

g

Clinical phenotypes

PID	Age	Sex	Race	Stage	TumorSize	Recurrence	AFP	MVI	HBV	HCV
ITH_41	68	Female	Malay	II	10	Yes	422	Yes	Neg	Neg

Patient 42: ITH_42

a

b

c

This patient is excluded
from DNA analysis due to low purity

d

e

f

g

Clinical phenotypes

PID	Age	Sex	Race	Stage	TumorSize	Recurrence	AFP	MVI	HBV	HCV
ITH_42	63	Male	Chinese	I	4.5	Yes	8.6	No	Neg	Neg

Patient 43: ITH_43

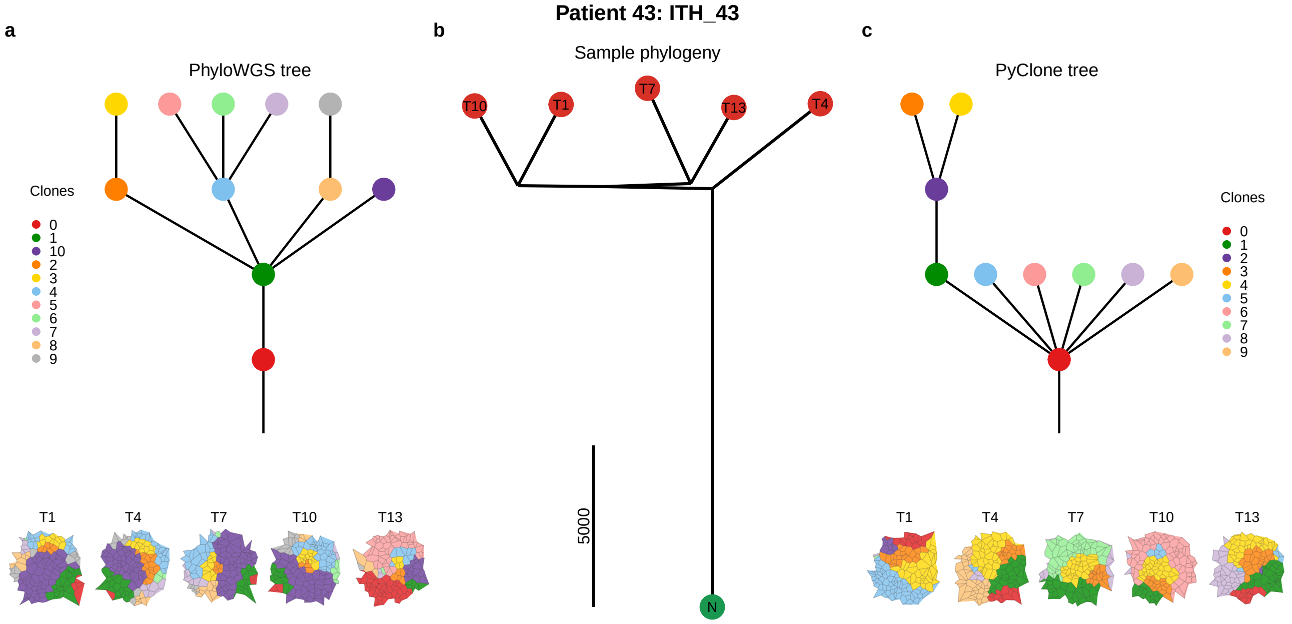

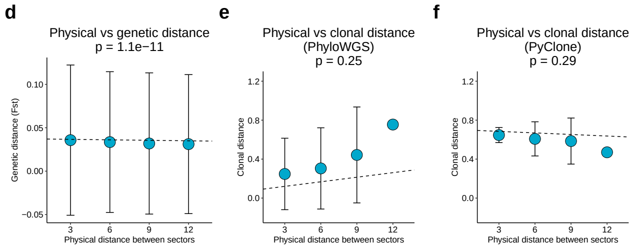

g

Clinical phenotypes

PID	Age	Sex	Race	Stage	TumorSize	Recurrence	AFP	MVI	HBV	HCV
ITH_43	60	Male	Chinese	III	14	Yes	17349	Yes	Neg	Neg

Patient 44: ITH_44

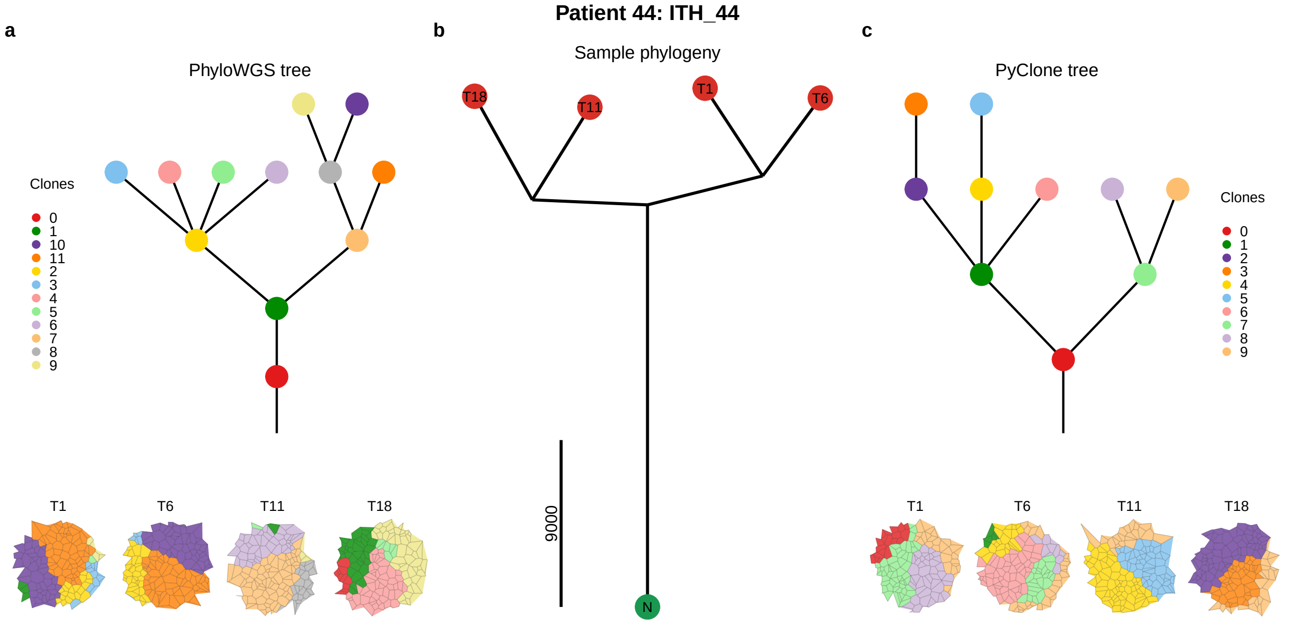

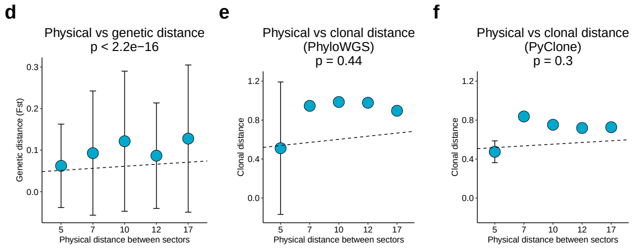

g

Clinical phenotypes

PID	Age	Sex	Race	Stage	TumorSize	Recurrence	AFP	MVI	HBV	HCV
ITH_44	75	Male	Chinese	I	11	Yes	3.9	No	Pos	Neg

Patient 45: ITH_45

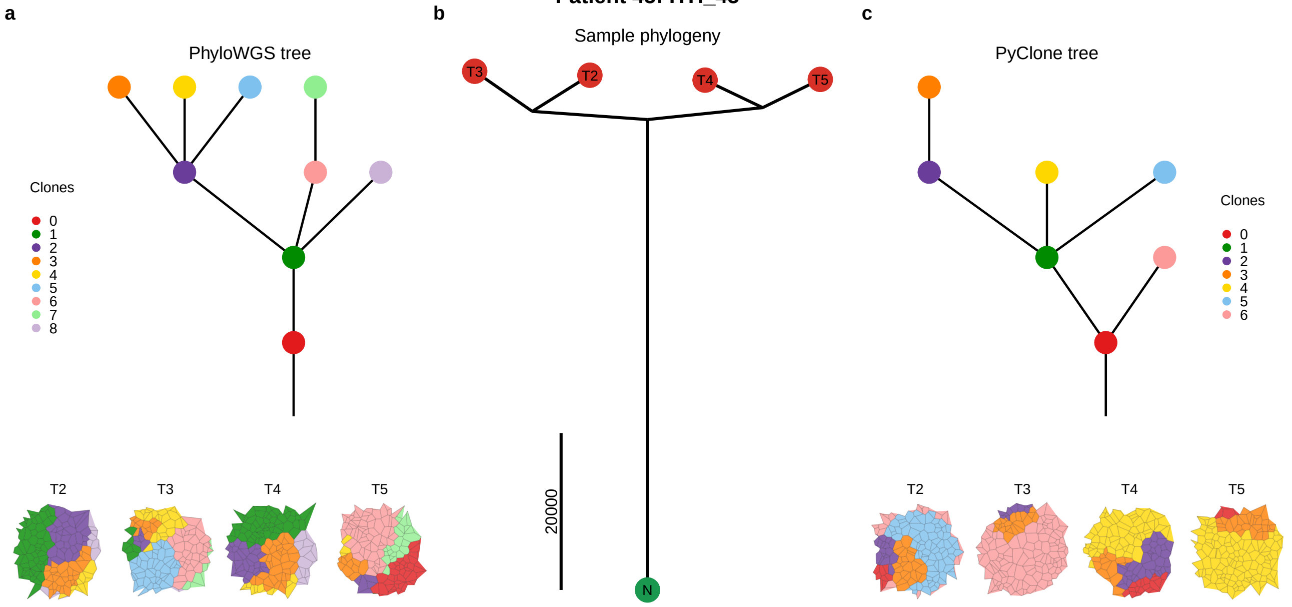

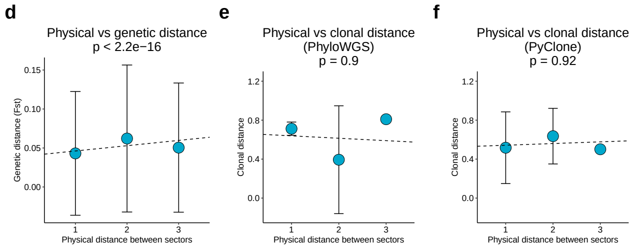

g

Clinical phenotypes

PID	Age	Sex	Race	Stage	TumorSize	Recurrence	AFP	MVI	HBV	HCV
ITH_45	54	Female	Chinese	I	4.8	No	4.6	No	Pos	Neg

Patient 46: ITH_46

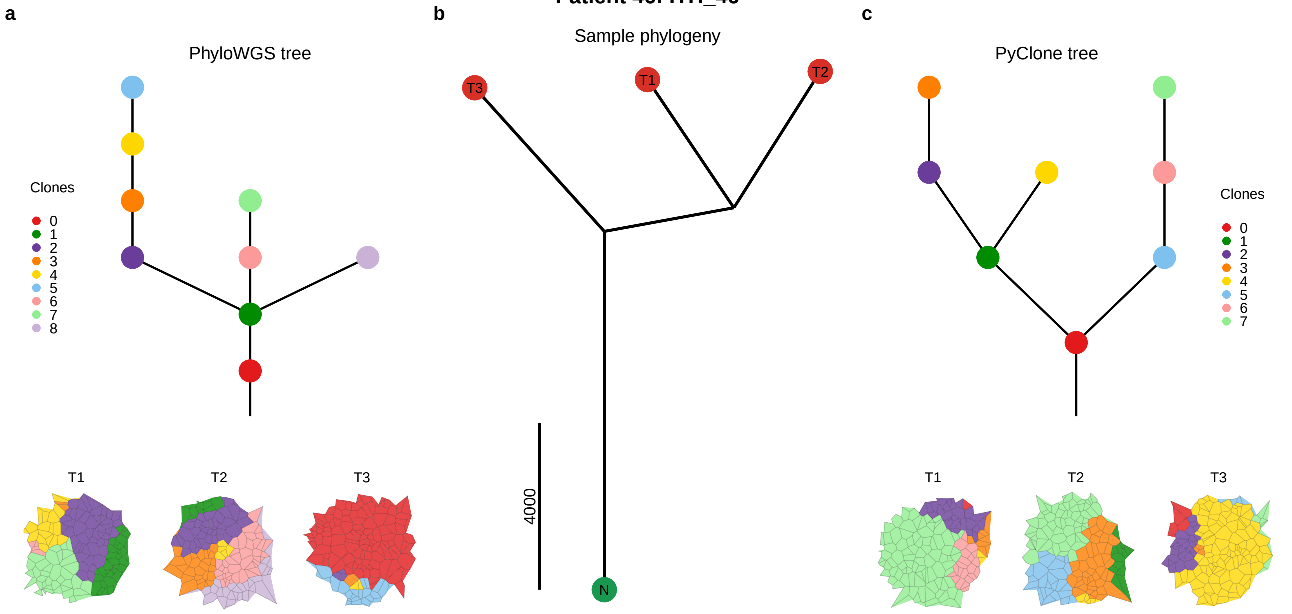

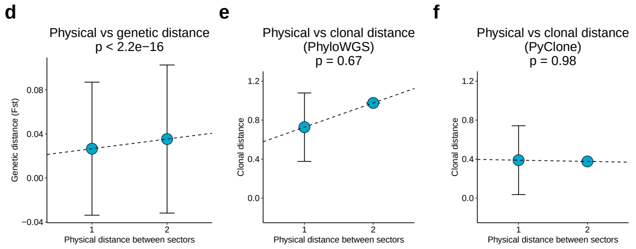

g

Clinical phenotypes

PID	Age	Sex	Race	Stage	TumorSize	Recurrence	AFP	MVI	HBV	HCV
ITH_46	55	Male	Chinese	II	3.2	Yes	828.0	Yes	Pos	Neg

Patient 47: ITH_47

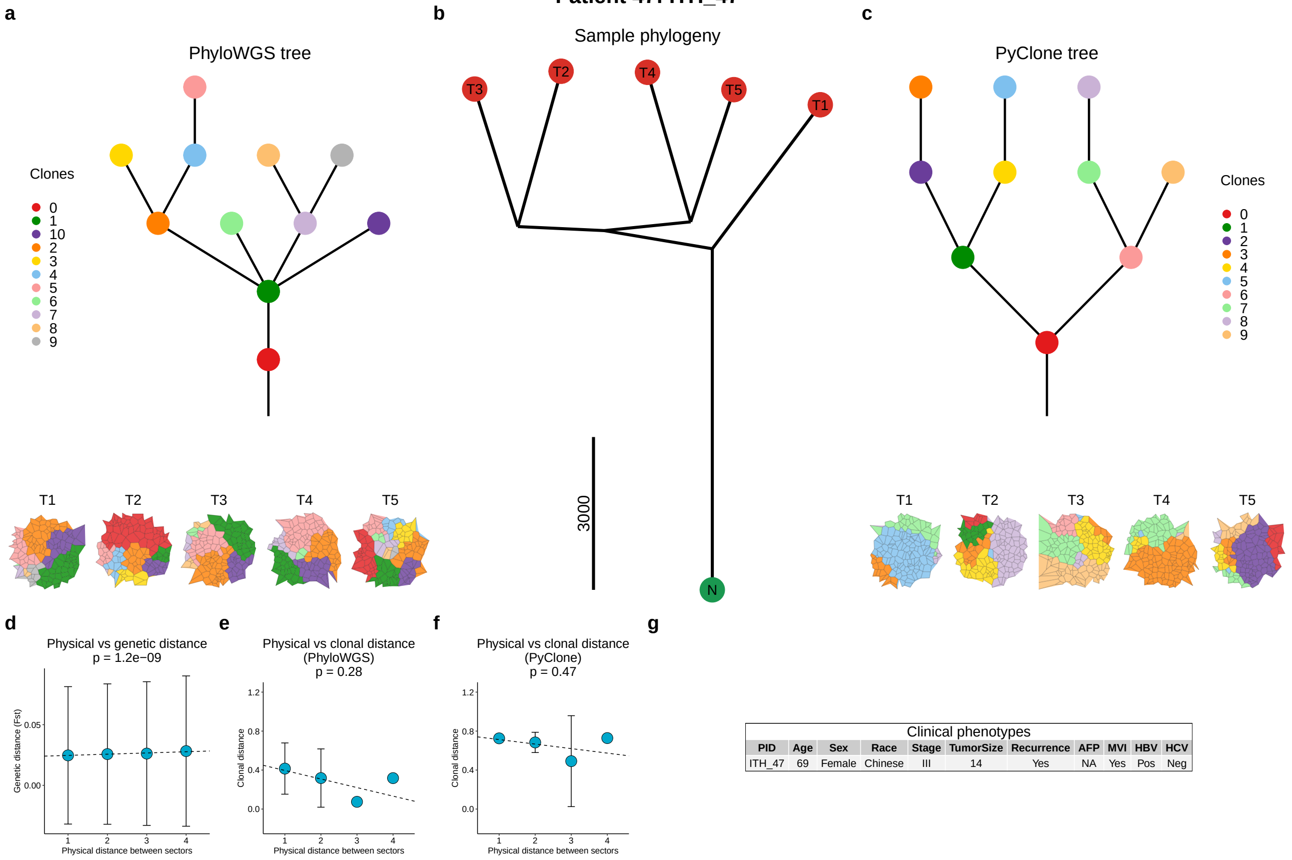

g

Clinical phenotypes

PID	Age	Sex	Race	Stage	TumorSize	Recurrence	AFP	MVI	HBV	HCV
ITH_47	69	Female	Chinese	III	14	Yes	NA	Yes	Pos	Neg

Patient 48: ITH_48

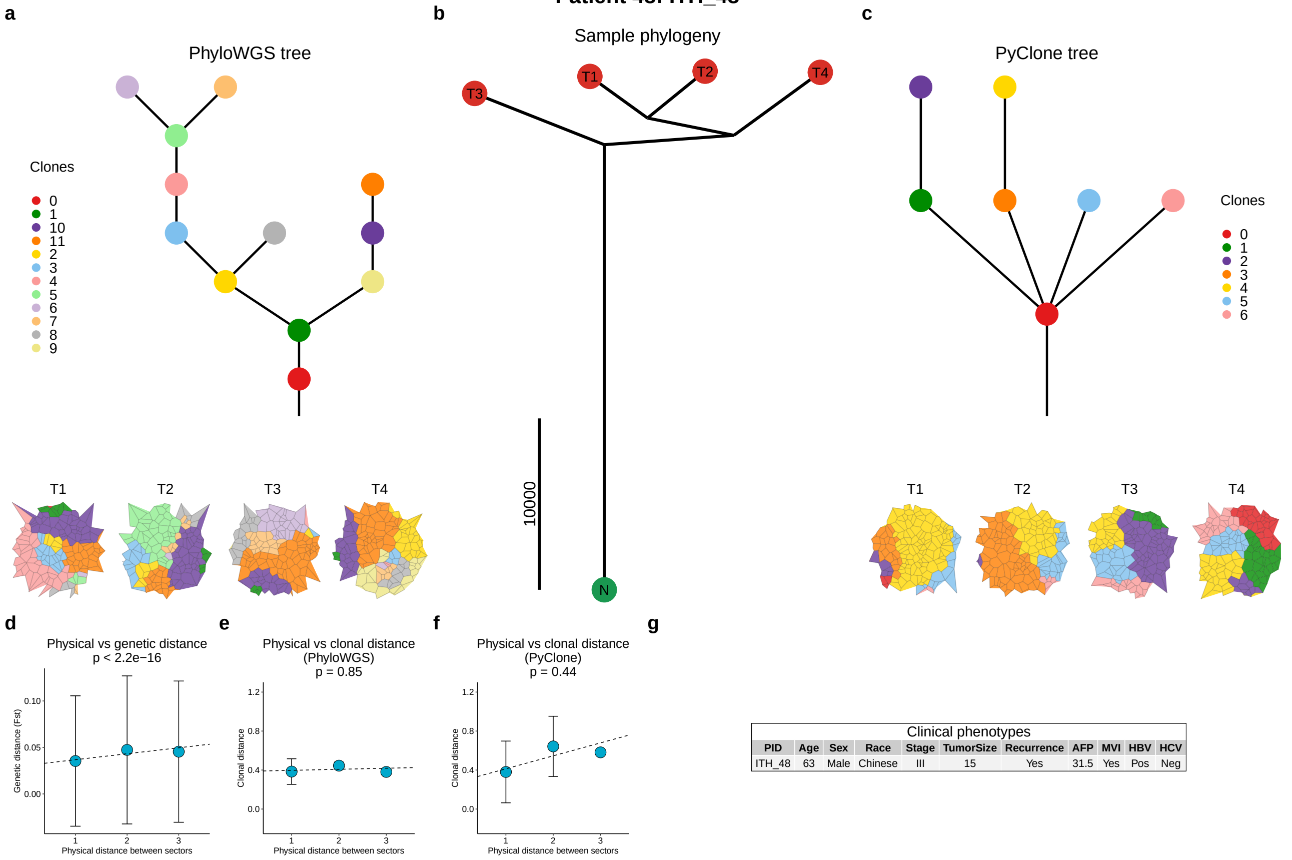

g

Clinical phenotypes

PID	Age	Sex	Race	Stage	TumorSize	Recurrence	AFP	MVI	HBV	HCV
ITH_48	63	Male	Chinese	III	15	Yes	31.5	Yes	Pos	Neg

Patient 49: ITH_49

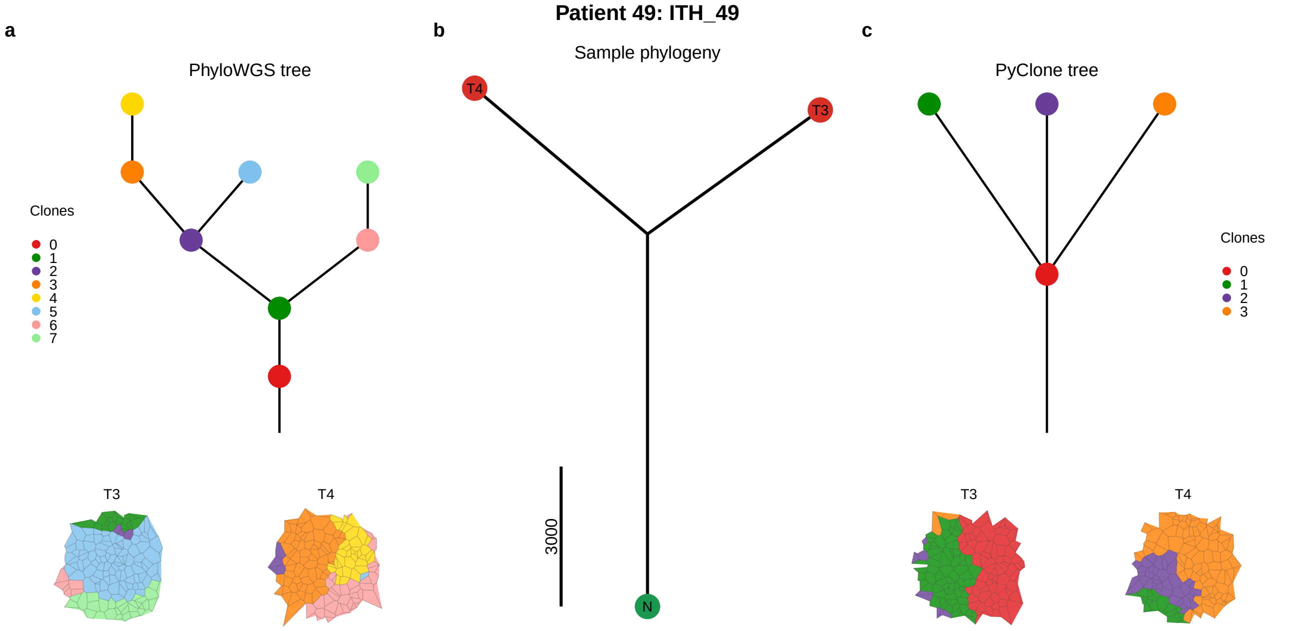

d

This patient does not have Fst plot (only 2 samples)

e

This patient does not have Cosine (PhyloWGS) plot (only 2 samples)

f

This patient does not have Cosine (PyClone) plot (only 2 samples)

g

Clinical phenotypes

PID	Age	Sex	Race	Stage	TumorSize	Recurrence	AFP	MVI	HBV	HCV
ITH_49	50	Female	Chinese	I	7	No	NA	No	Pos	Neg

Patient 50: ITH_50

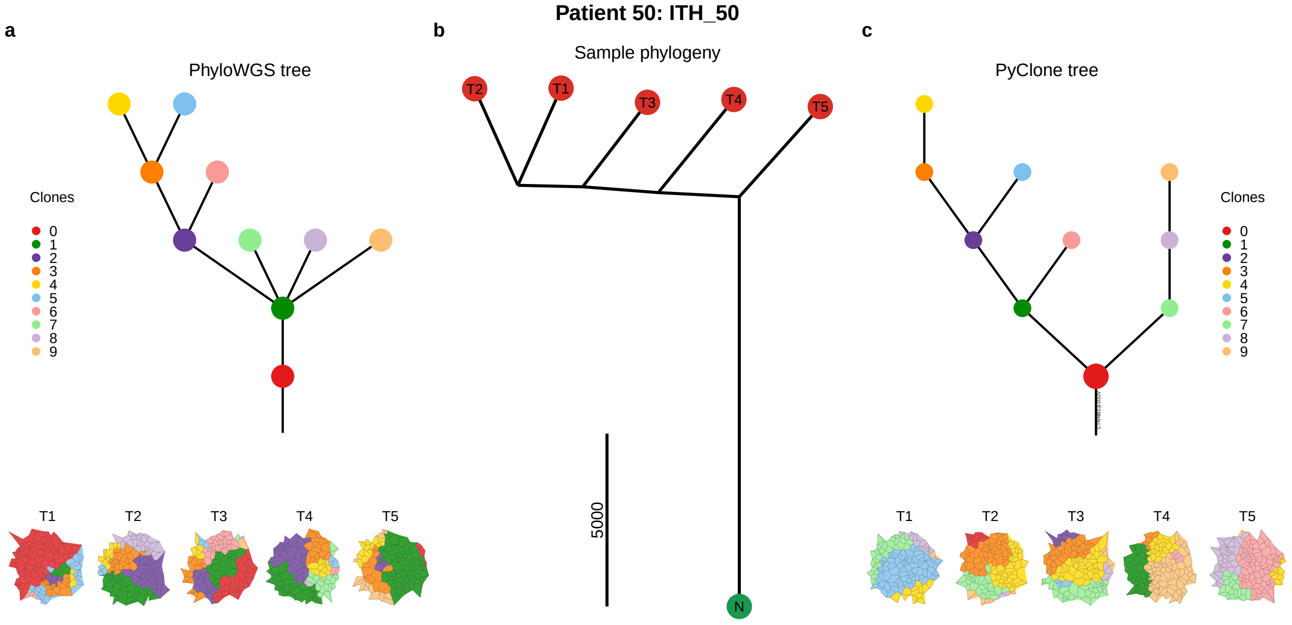

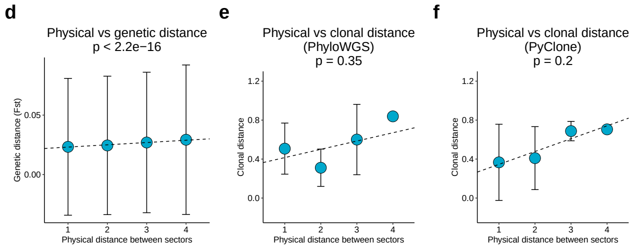

g

Clinical phenotypes

PID	Age	Sex	Race	Stage	TumorSize	Recurrence	AFP	MVI	HBV	HCV
ITH_50	78	Male	Chinese	III	7	Yes	36.6	Yes	Pos	Neg

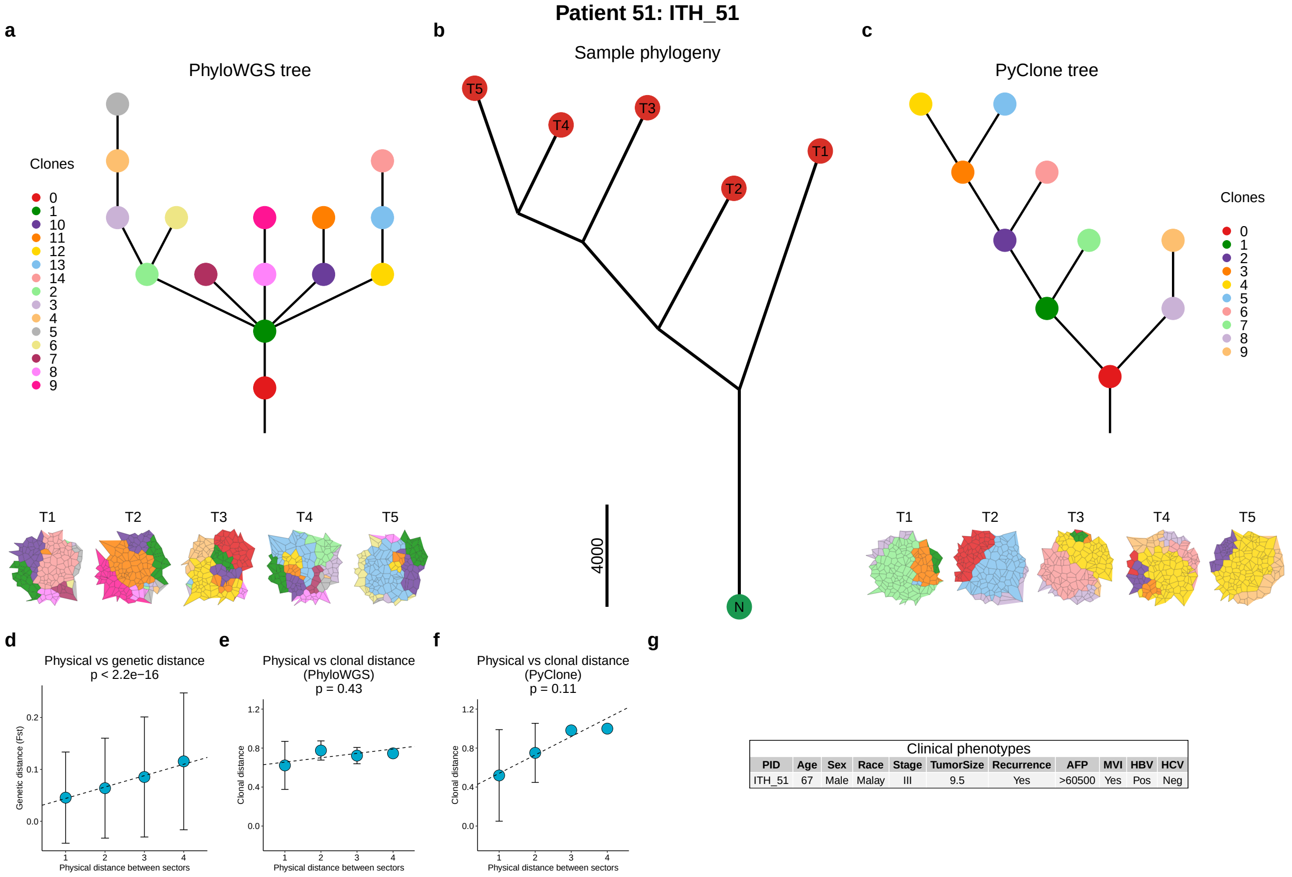

Clinical phenotypes

PID	Age	Sex	Race	Stage	TumorSize	Recurrence	AFP	MVI	HBV	HCV
ITH_51	67	Male	Malay	III	9.5	Yes	>60500	Yes	Pos	Neg

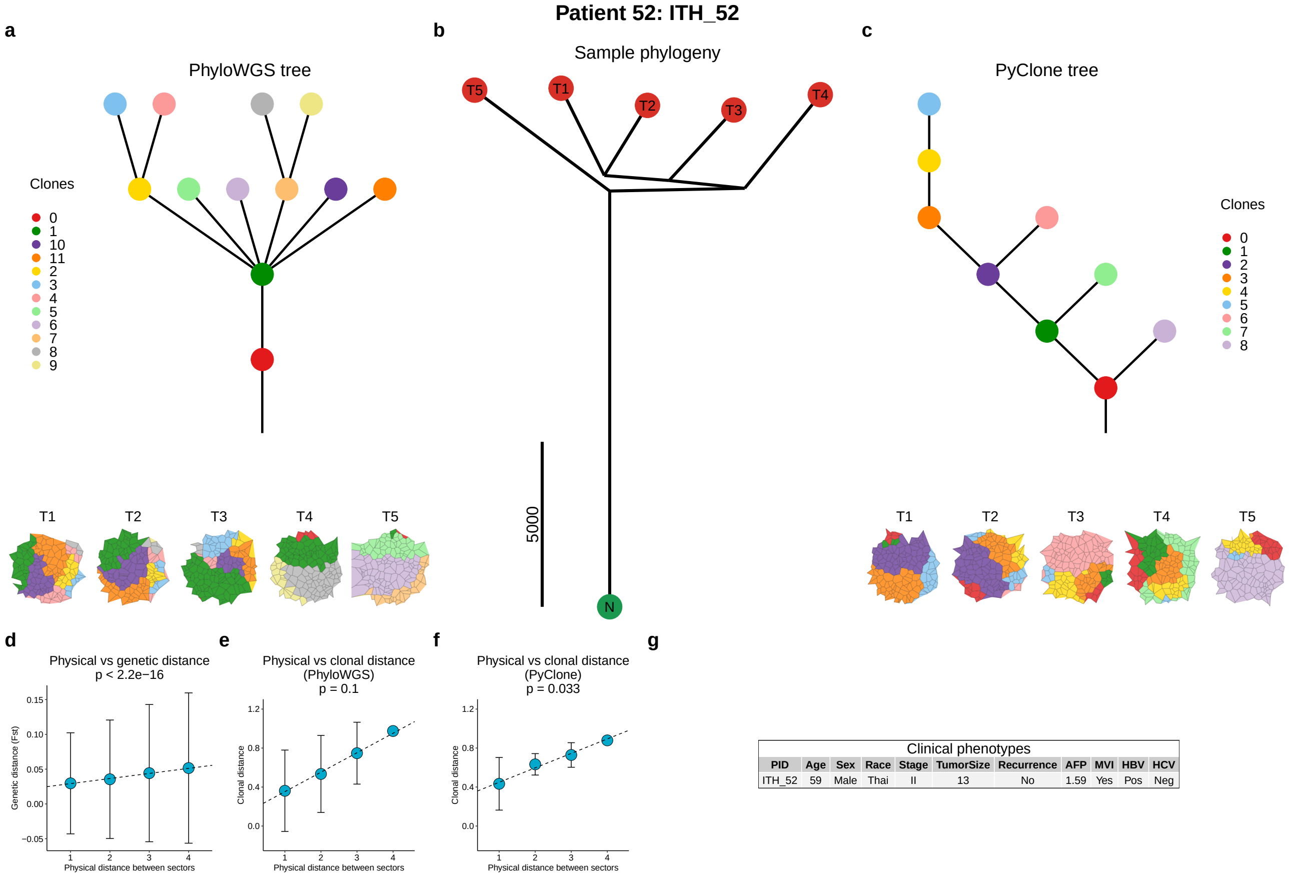

Clinical phenotypes

PID	Age	Sex	Race	Stage	TumorSize	Recurrence	AFP	MVI	HBV	HCV
ITH_52	59	Male	Thai	II	13	No	1.59	Yes	Pos	Neg

Patient 53: ITH_53

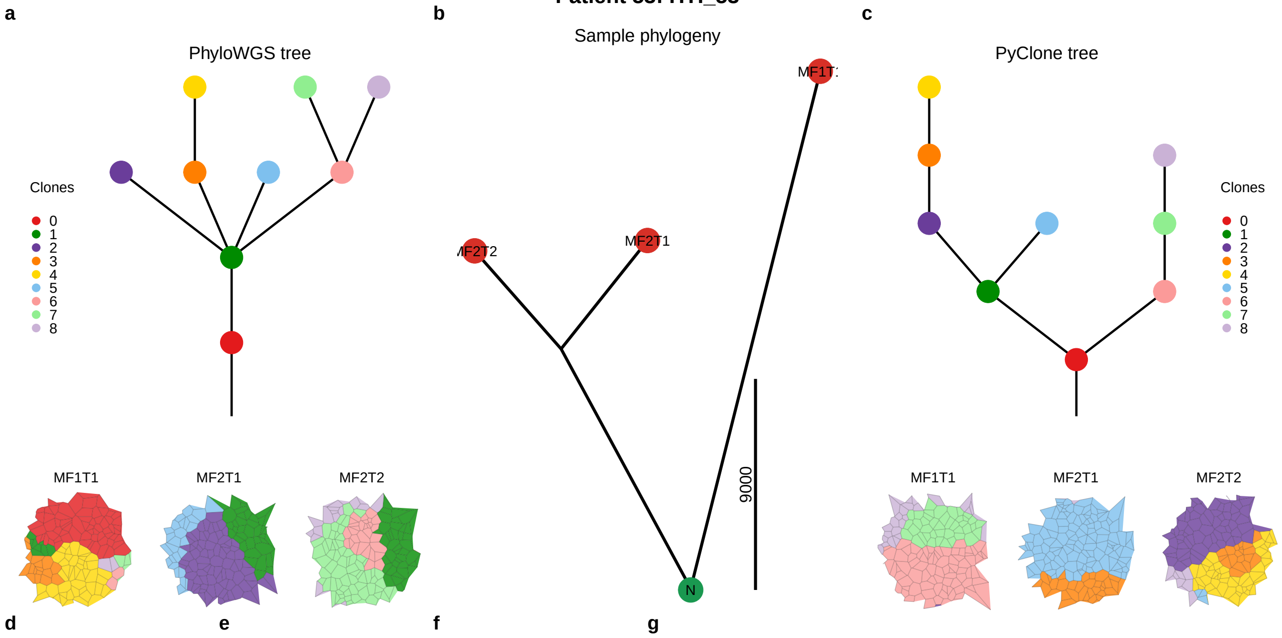

This patient does not have Fst plot (multifocal)

This patient does not have Fst plot (multifocal)

This patient does not have Fst plot (multifocal)

Clinical phenotypes

PID	Age	Sex	Race	Stage	TumorSize	Recurrence	AFP	MVI	HBV	HCV
ITH_53	67	Male	Thai	II	1.5	Yes	831.7	No	Pos	Neg

Patient 54: ITH_54

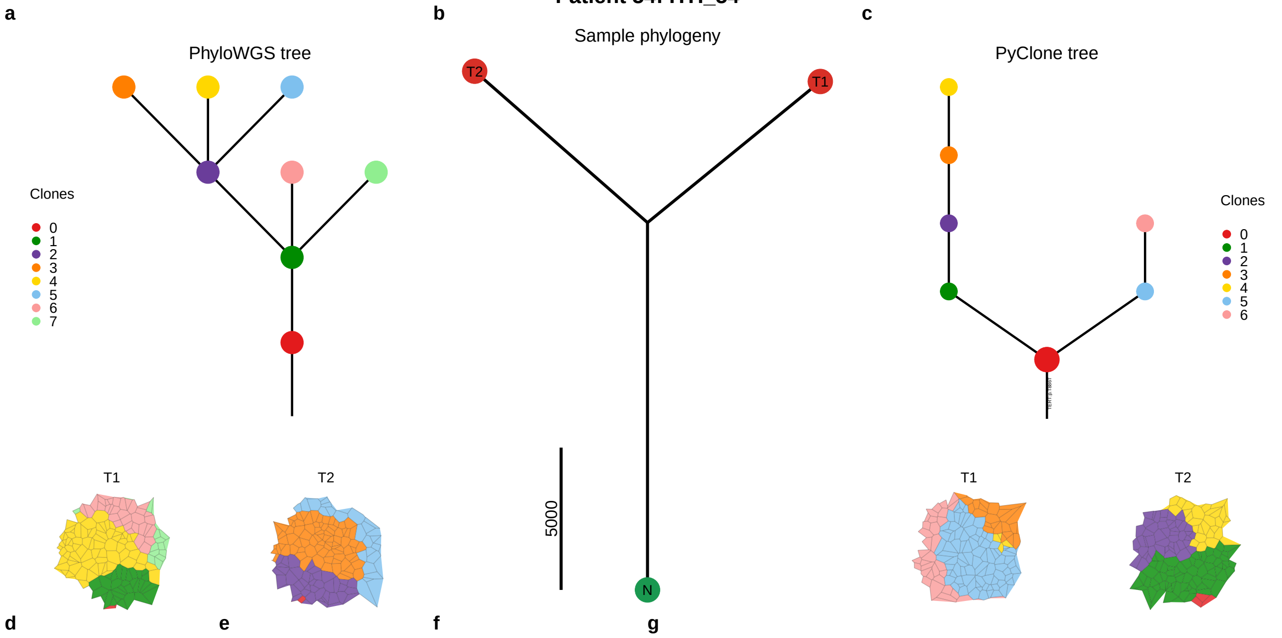

This patient does not have Fst plot (only 2 samples)

This patient does not have Cosine (PhyloWGS) plot (only 2 samples)

This patient does not have Cosine (PyClone) plot (only 2 samples)

Clinical phenotypes

PID	Age	Sex	Race	Stage	TumorSize	Recurrence	AFP	MVI	HBV	HCV
ITH_54	73	Female	Chinese	I	2.5	No	5.4	No	Neg	Neg

Patient 55: ITH_55

a

This patient does not have
PhyloWGS tree (polyclonal error)

b

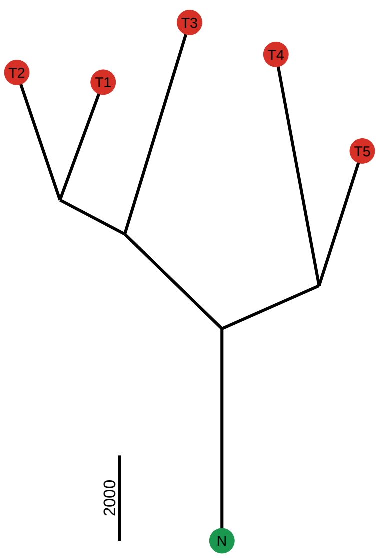

c

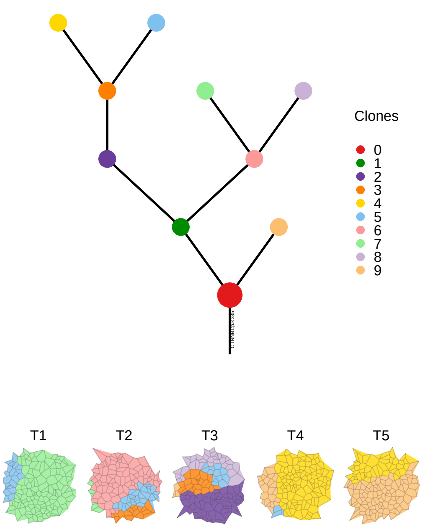

d

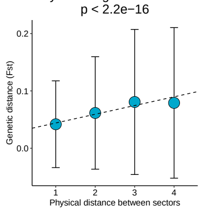

e

This patient does not have
Cosine (PhyloWGS) plot (polyclonal error)

f

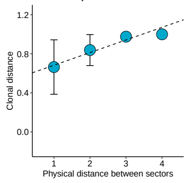

g

Clinical phenotypes

PID	Age	Sex	Race	Stage	TumorSize	Recurrence	AFP	MVI	HBV	HCV
ITH_55	70	Male	Chinese	I	5.5	No	1.0	No	Pos	Neg

Patient 56: ITH_56

a

b

c

This patient is excluded
from DNA analysis due to low purity

d

e

f

g

Clinical phenotypes

PID	Age	Sex	Race	Stage	TumorSize	Recurrence	AFP	MVI	HBV	HCV
ITH_56	61	Female	Chinese	I	2	No	3.5	No	Pos	Neg

Patient 57: ITH_57

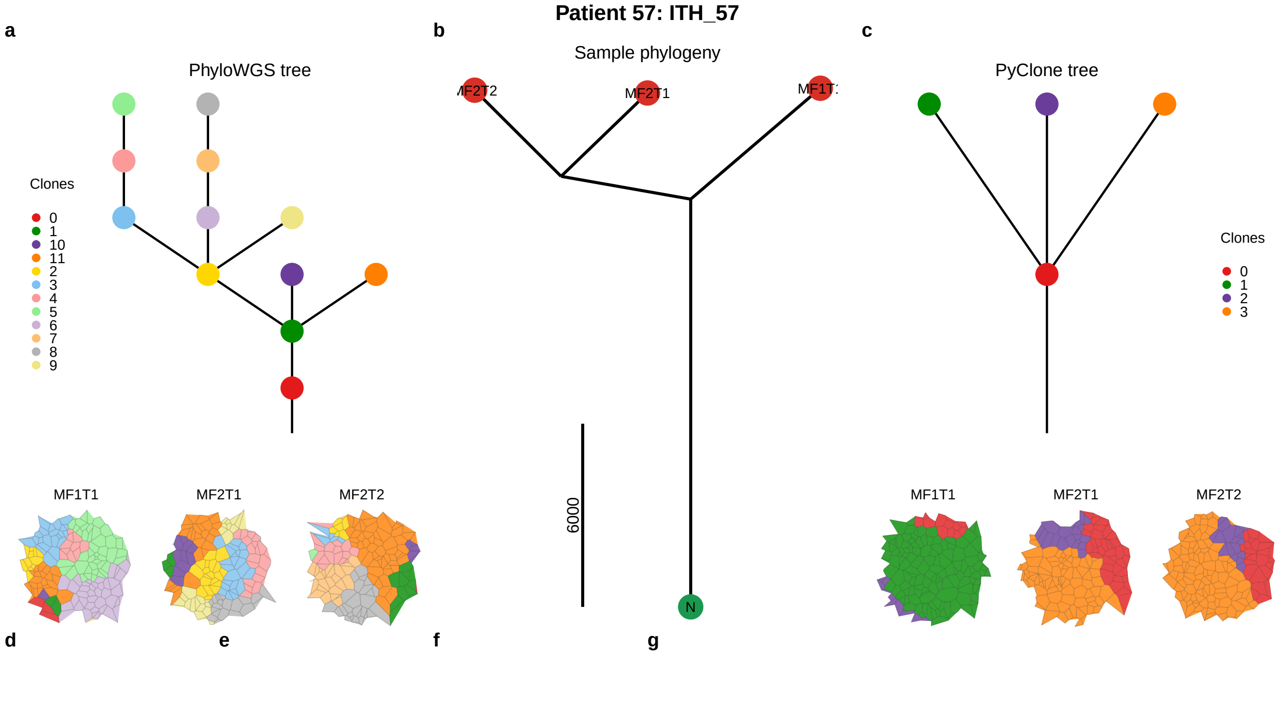

This patient does not have Fst plot (multifocal)

This patient does not have Fst plot (multifocal)

This patient does not have Fst plot (multifocal)

Clinical phenotypes

PID	Age	Sex	Race	Stage	TumorSize	Recurrence	AFP	MVI	HBV	HCV
ITH_57	72	M	Chinese	II	3.5	No	3	No	Pos	Neg

Patient 58: ITH_58

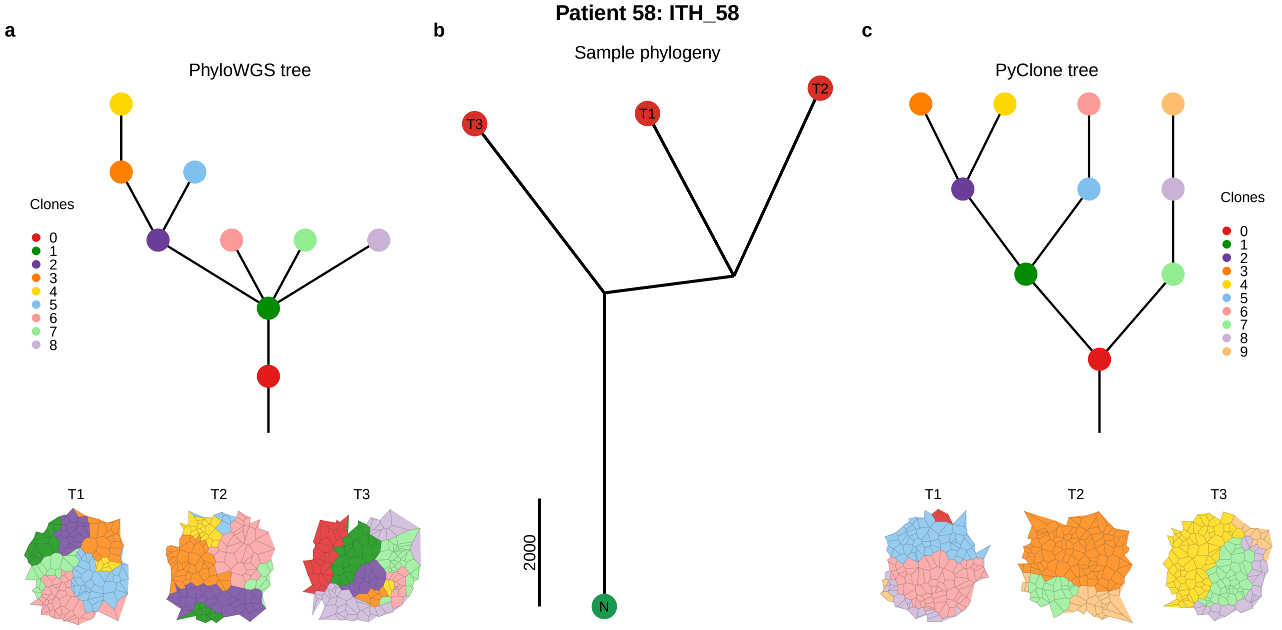

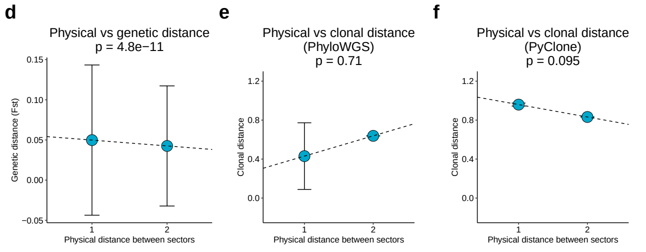

Clinical phenotypes

PID	Age	Sex	Race	Stage	TumorSize	Recurrence	AFP	MVI	HBV	HCV
ITH_58	69	F	Chinese	I	8.5	Yes	1021	No	Pos	Neg

Patient 59: ITH_59

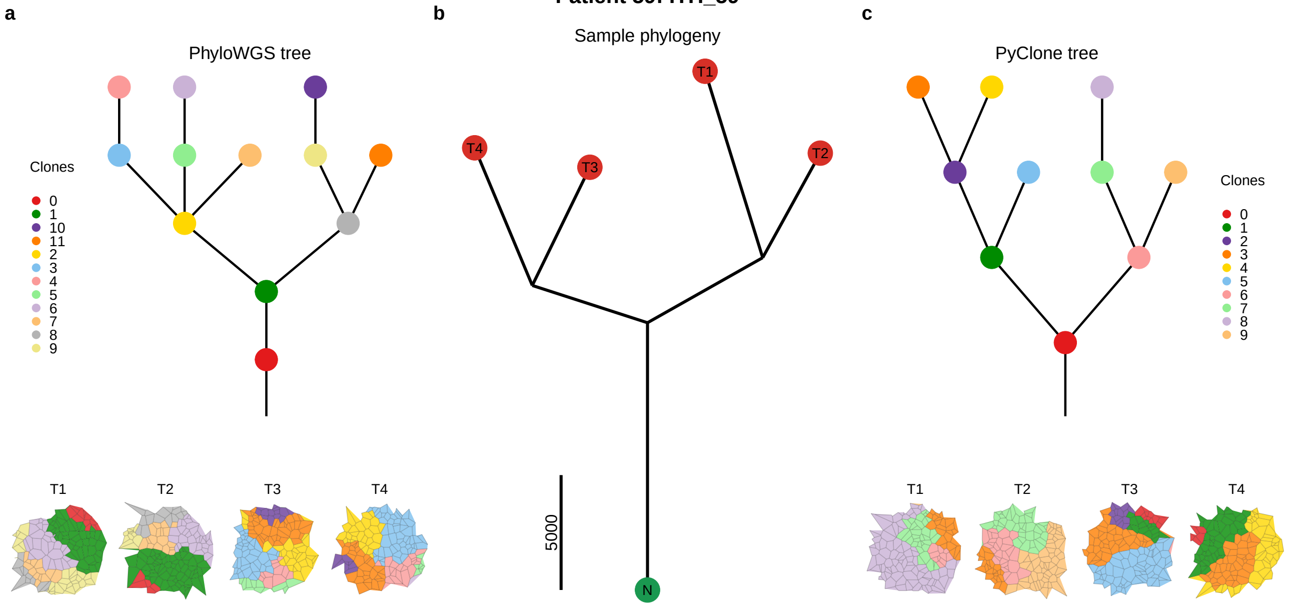

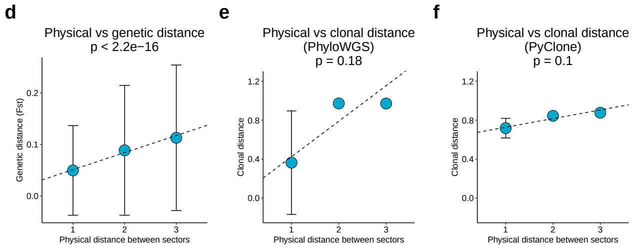

Clinical phenotypes

PID	Age	Sex	Race	Stage	TumorSize	Recurrence	AFP	MVI	HBV	HCV
ITH_59	73	M	Chinese	II	14	No	1.5	Yes	Pos	Neg

Patient 60: ITH_60

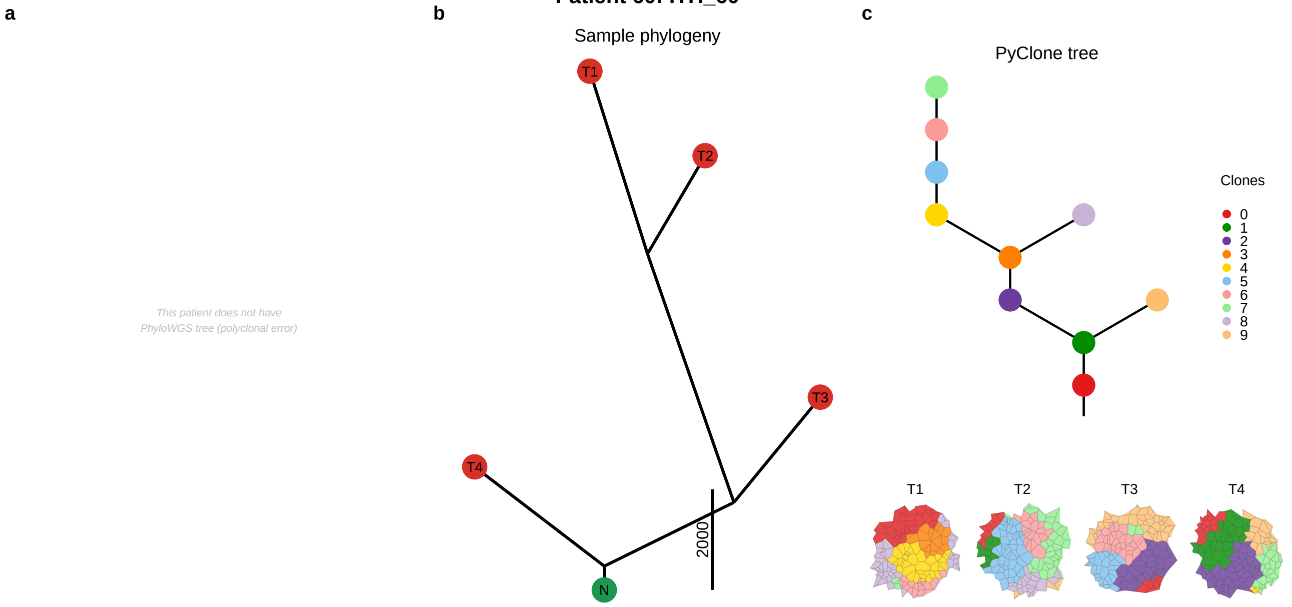

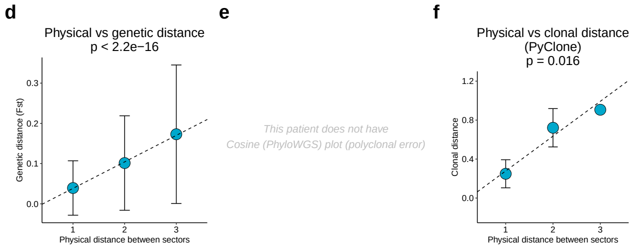

Clinical phenotypes

PID	Age	Sex	Race	Stage	TumorSize	Recurrence	AFP	MVI	HBV	HCV
ITH_60	61	M	Indonesian Chinese	III	8	Yes	144	Yes	Neg	Neg

Patient 61: ITH_61

a

b

c

This patient is excluded
from DNA analysis due to low purity

d

e

f

g

Clinical phenotypes

PID	Age	Sex	Race	Stage	TumorSize	Recurrence	AFP	MVI	HBV	HCV
ITH_61	70	F	Malay	I	3.8	No	248	No	Neg	Neg

Patient 62: ITH_62

a

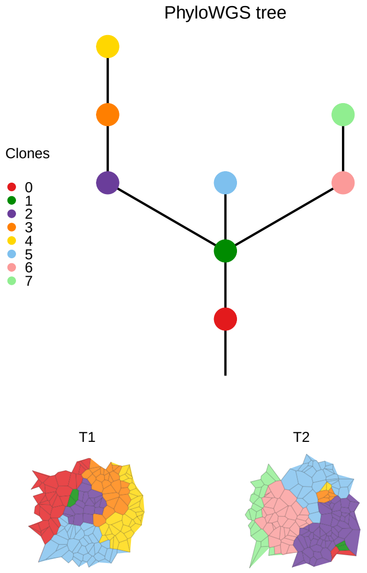

b

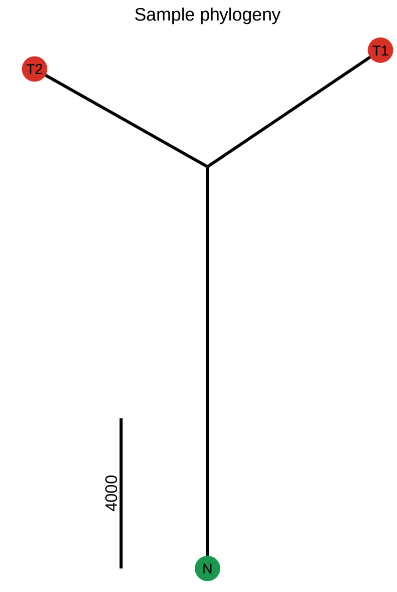

c

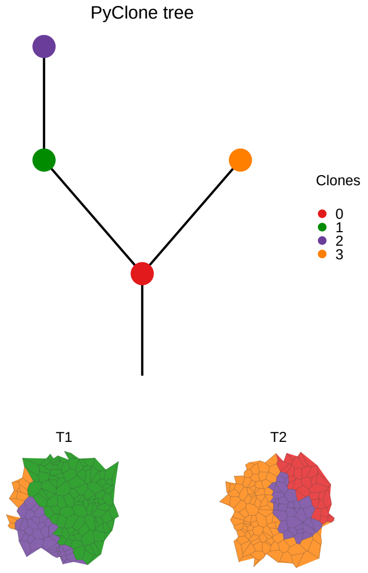

d

This patient does not have
Fst plot (only 2 samples)

e

This patient does not have
Cosine (PhyloWGS) plot (only 2 samples)

f

This patient does not have
Cosine (PyClone) plot (only 2 samples)

g

Clinical phenotypes

PID	Age	Sex	Race	Stage	TumorSize	Recurrence	AFP	MVI	HBV	HCV
ITH_62	63	M	Chinese	I	2.2	No	6.1	No	Pos	Neg

Patient 63: ITH_63

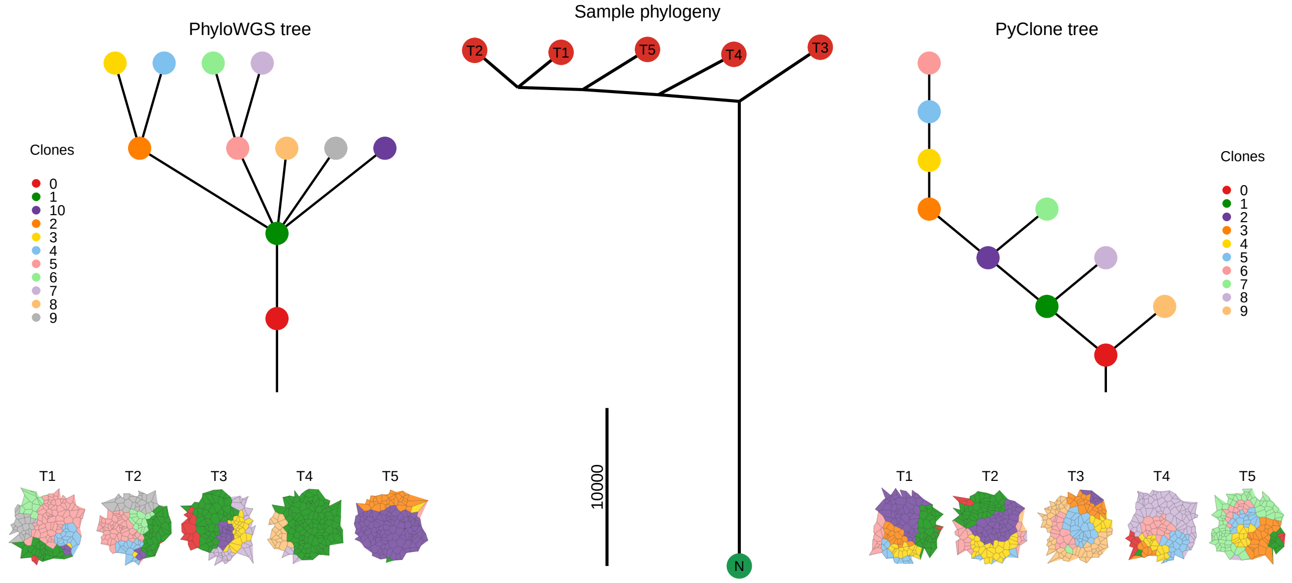

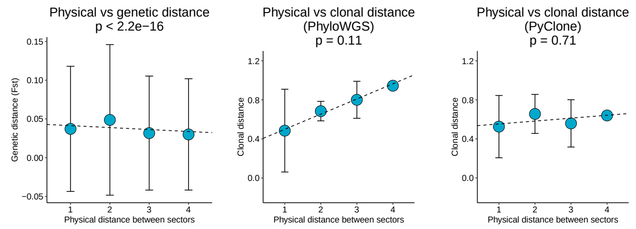

g

Clinical phenotypes

PID	Age	Sex	Race	Stage	TumorSize	Recurrence	AFP	MVI	HBV	HCV
ITH_63	47	F	Chinese	II	7.5	No	2203	Yes	Neg	Neg

Patient 64: ITH_64

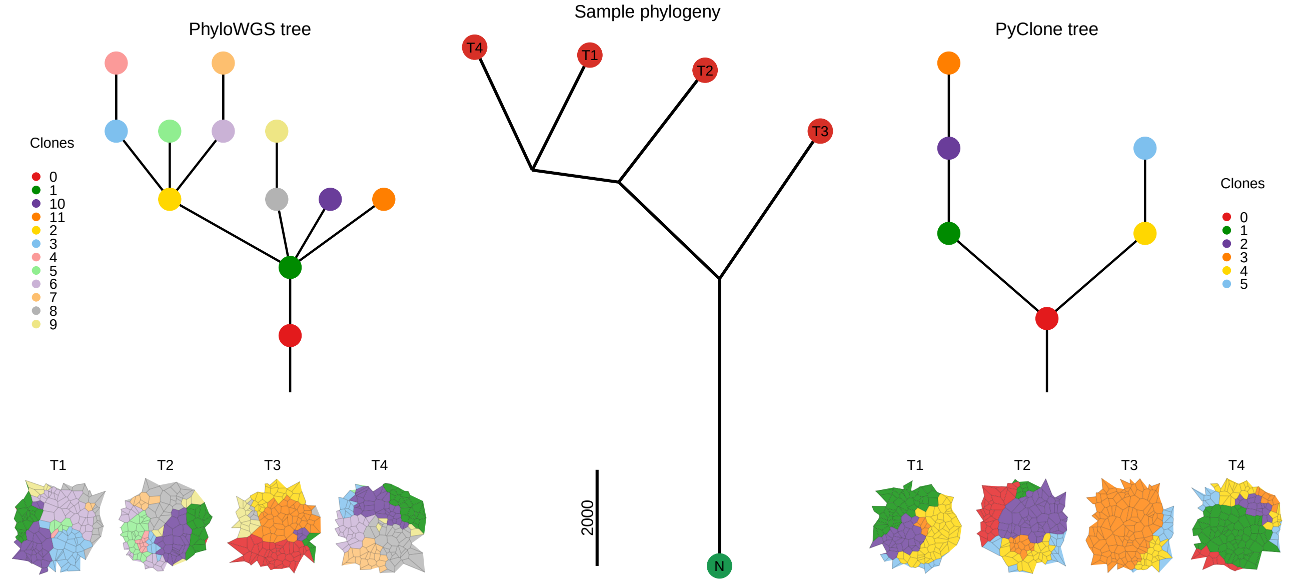

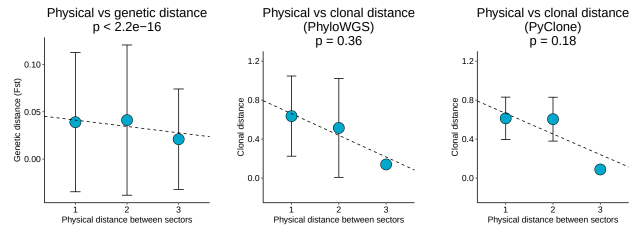

g

Clinical phenotypes

PID	Age	Sex	Race	Stage	TumorSize	Recurrence	AFP	MVI	HBV	HCV
ITH_64	71	M	Chinese	I	4.7	No	8920	No	Pos	Neg

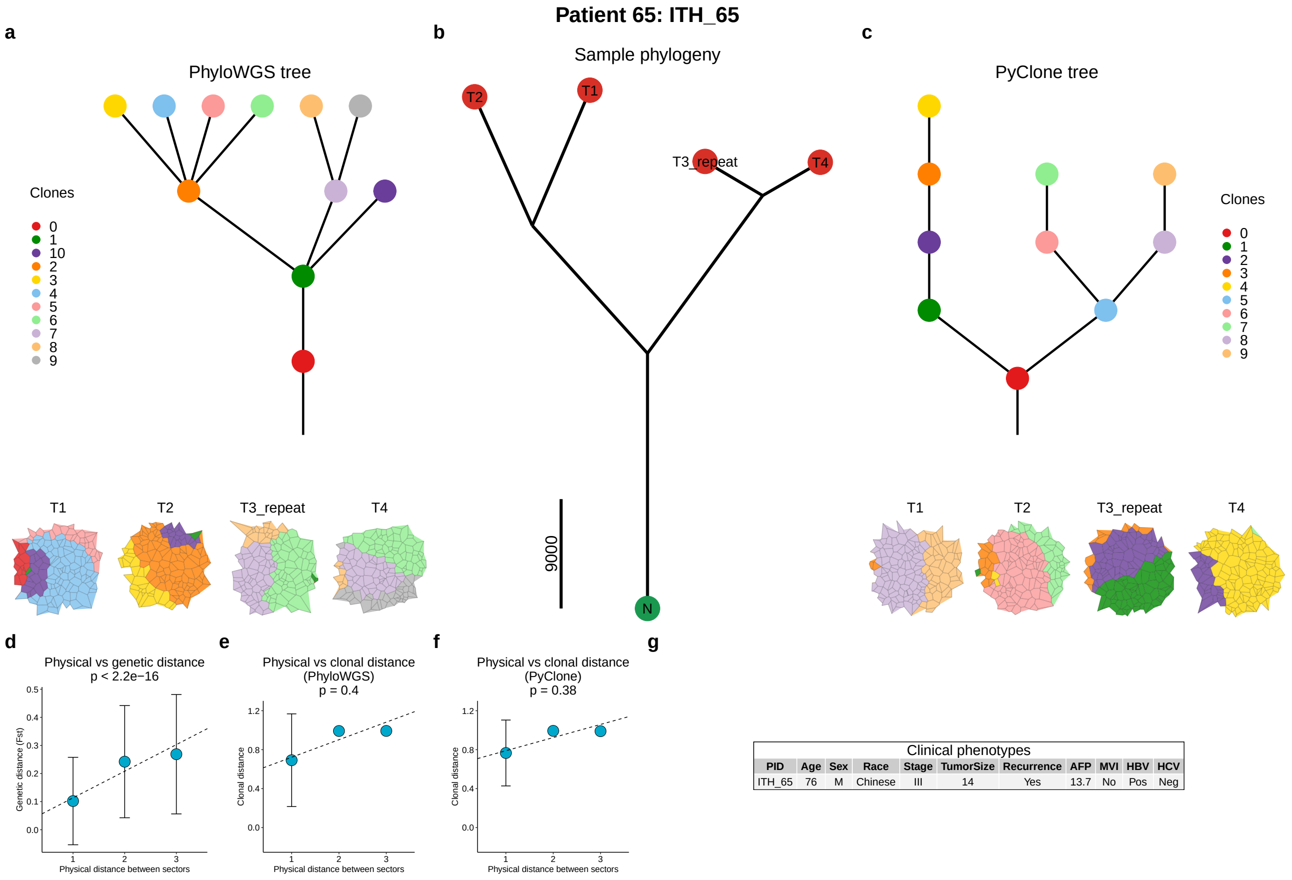

Clinical phenotypes

PID	Age	Sex	Race	Stage	TumorSize	Recurrence	AFP	MVI	HBV	HCV
ITH_65	76	M	Chinese	III	14	Yes	13.7	No	Pos	Neg

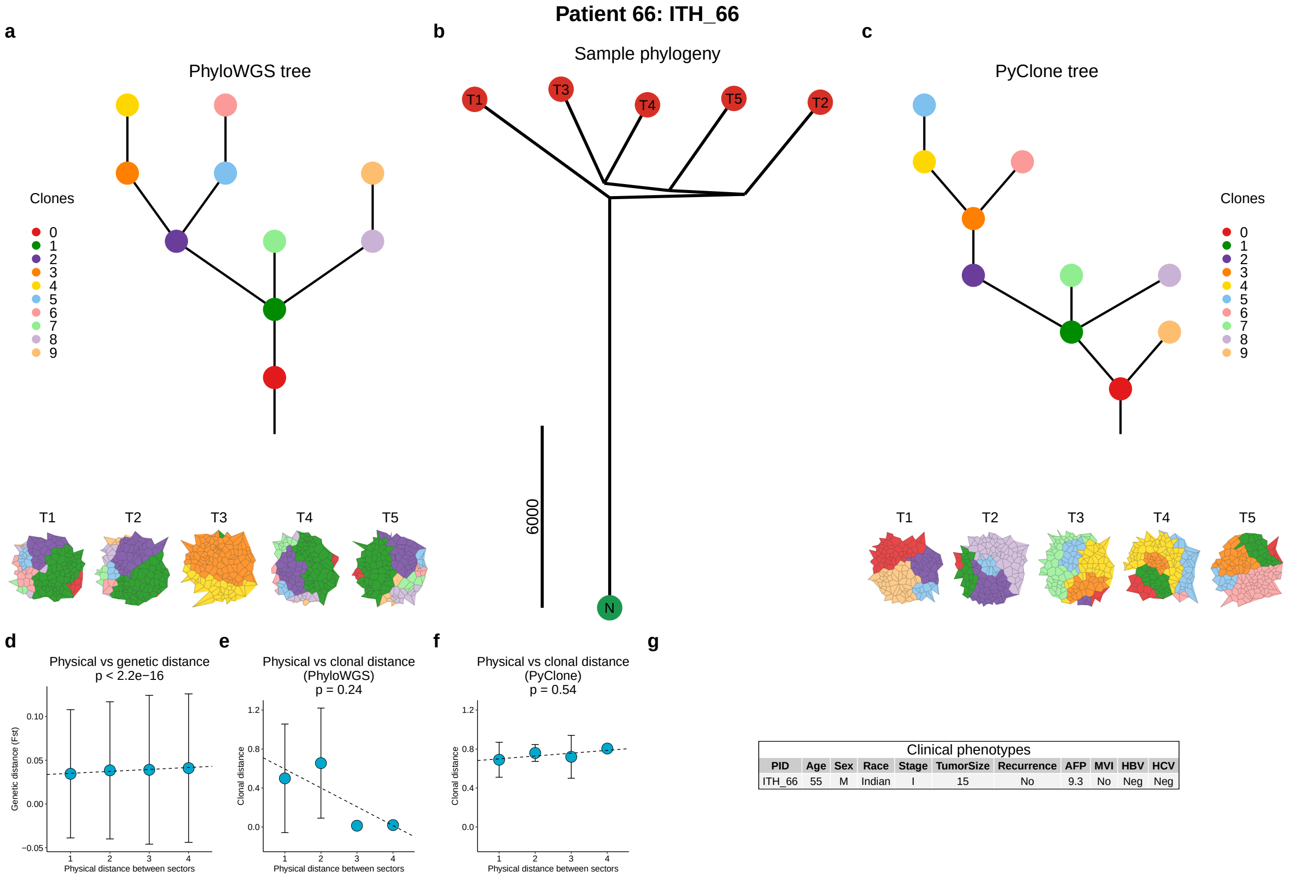

Clinical phenotypes

PID	Age	Sex	Race	Stage	TumorSize	Recurrence	AFP	MVI	HBV	HCV
ITH_66	55	M	Indian	I	15	No	9.3	No	Neg	Neg

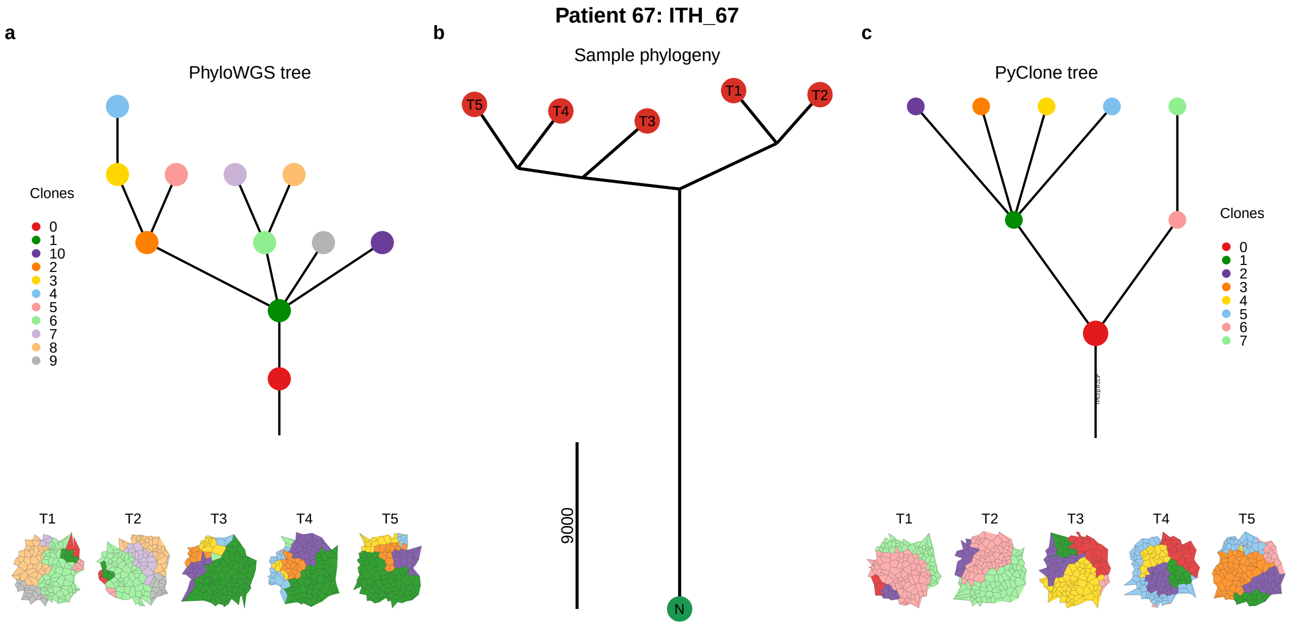

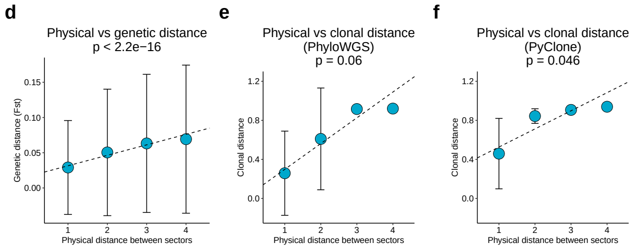

g

Clinical phenotypes

PID	Age	Sex	Race	Stage	TumorSize	Recurrence	AFP	MVI	HBV	HCV
ITH_67	75	M	Chinese	II	6.2	Yes	504	Yes	Pos	Neg

Supplementary Figure 7: **Clonal deconvolution of the patient cohort.** (a). Optimal clonal phylogeny and clonal deconvolution from PhyloWGS. (b) Sample phylogeny. (c). Optimal clonal phylogeny and clonal deconvolution using PyClone and CITUP. (d) IBD pattern, the x-axis is the physical distances between the patient's sectors, and the y-axis is the genetic distance (Fst) between the sectors. For each of physical distances, Fst values of all pairwise sectors with the same physical distance were used to draw a boxplot. The regression line, slope and p-value were derived from linear regression model (Methods). (e). The relationship between physical distance and the cosine distance of the clonal composition (using PhyloWGS, see Methods). (f). The relationship between physical distance and the cosine distance of the clonal composition (using Pyclone, see Methods,). (g). Clinical phenotypes of the patient. All 67 patients are listed in this figure. Entries with missing values (e.g. patients with number of sectors <=2 or those with failed analysis during Pyclone or PhyloWGS analysis) will be denoted in the figure.

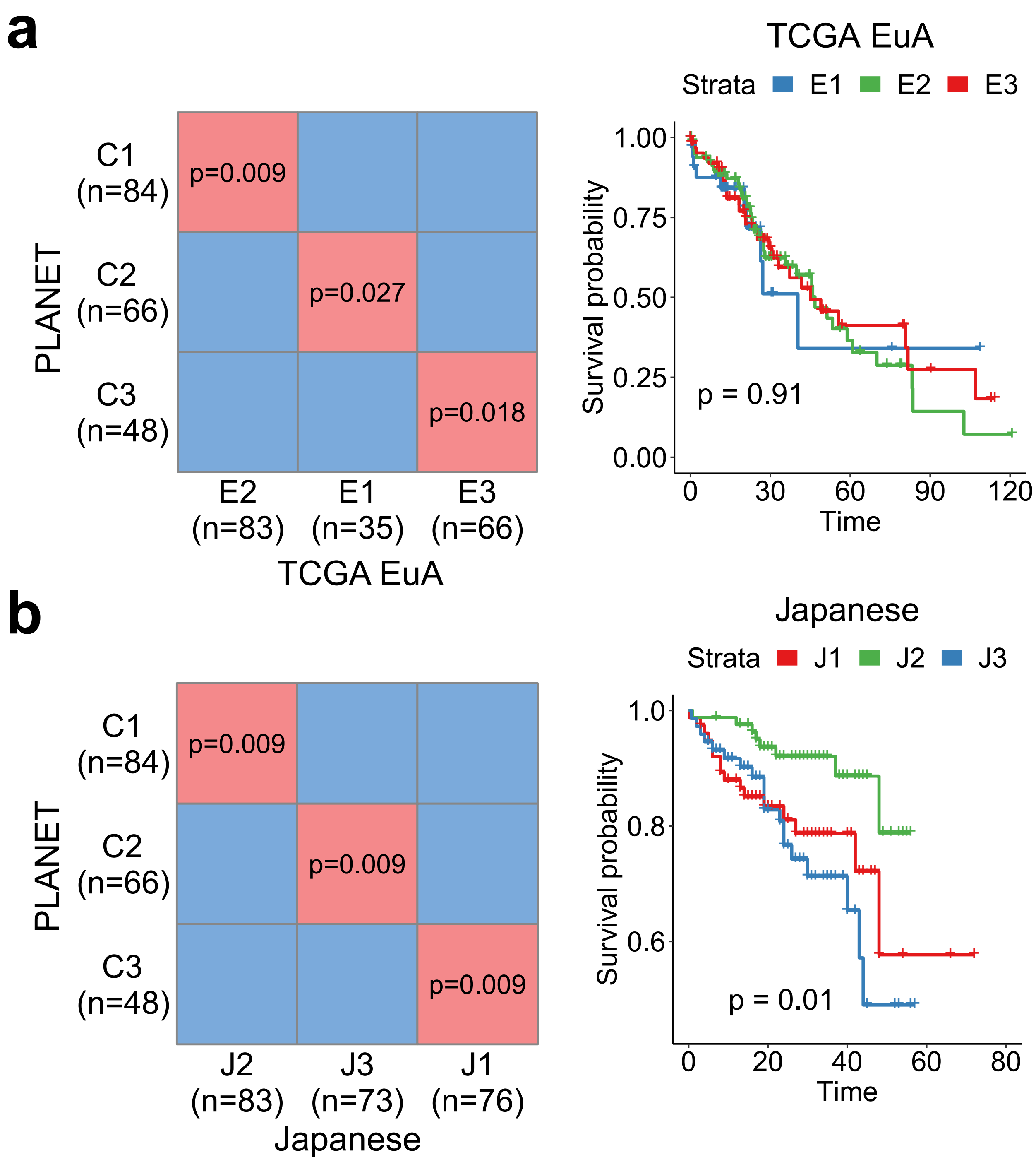

Supplementary Figure 8: **RNA subtypes and public cohorts.** (a) Submap match between the PLANET and the TCGA European cohort (left) and survival plot of the three subtypes in the TCGA European cohort. (b) Submap match between the PLANET and the Japanese cohort (left) and survival plot of the three subtypes in the Japanese cohort.

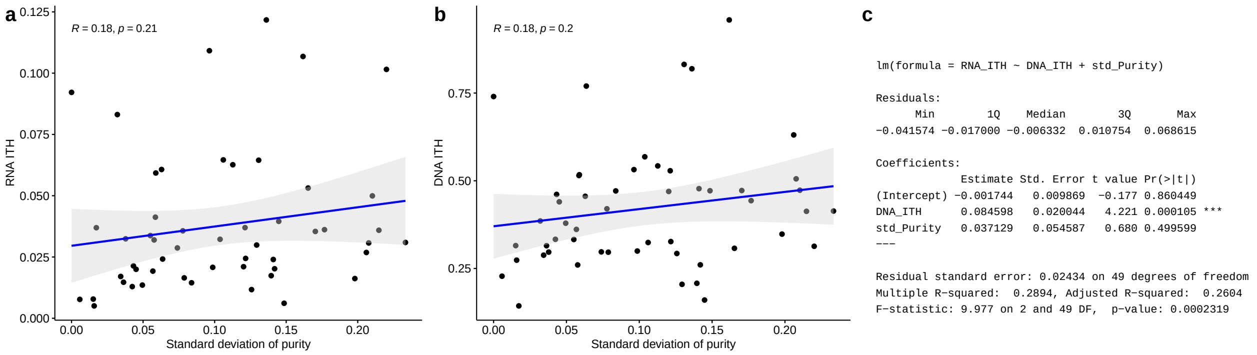

Supplementary Figure 9: **Genomic ITH and tumor purity.** a) Correlation between RNA ITH and standard deviation of purity (i.e. SD(purity)). b) Correlation between DNA ITH and SD(purity). c) Linear regression between RNA ITH and DNA ITH conditioning on SD(purity)

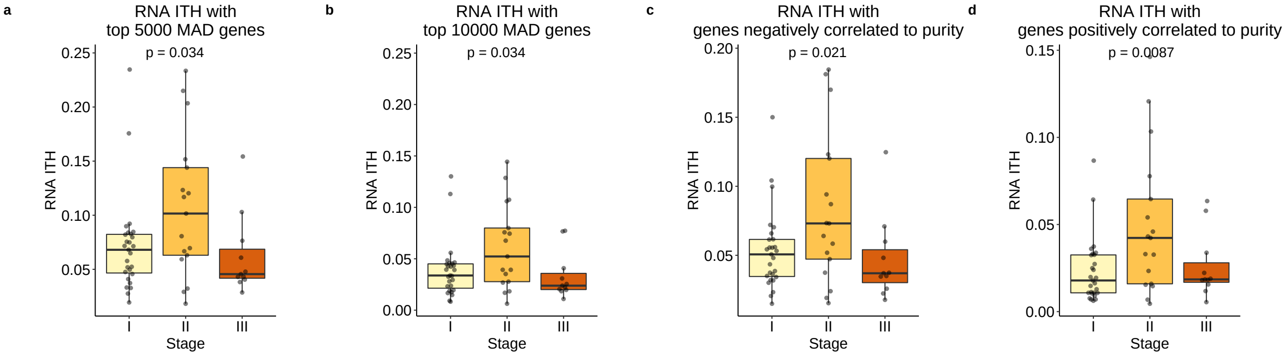

Supplementary Figure 10: **ITH and tumor stage.** a) RNA ITH calculated using top 5000 most variable genes (using Median Absolute Deviation, MAD). b) RNA ITH calculated using top 10000 most variable genes. c) RNA ITH calculated using genes negatively correlated with tumor purity (n=2648), d) RNA ITH calculated using genes positively correlated with tumor purity (n=1461).

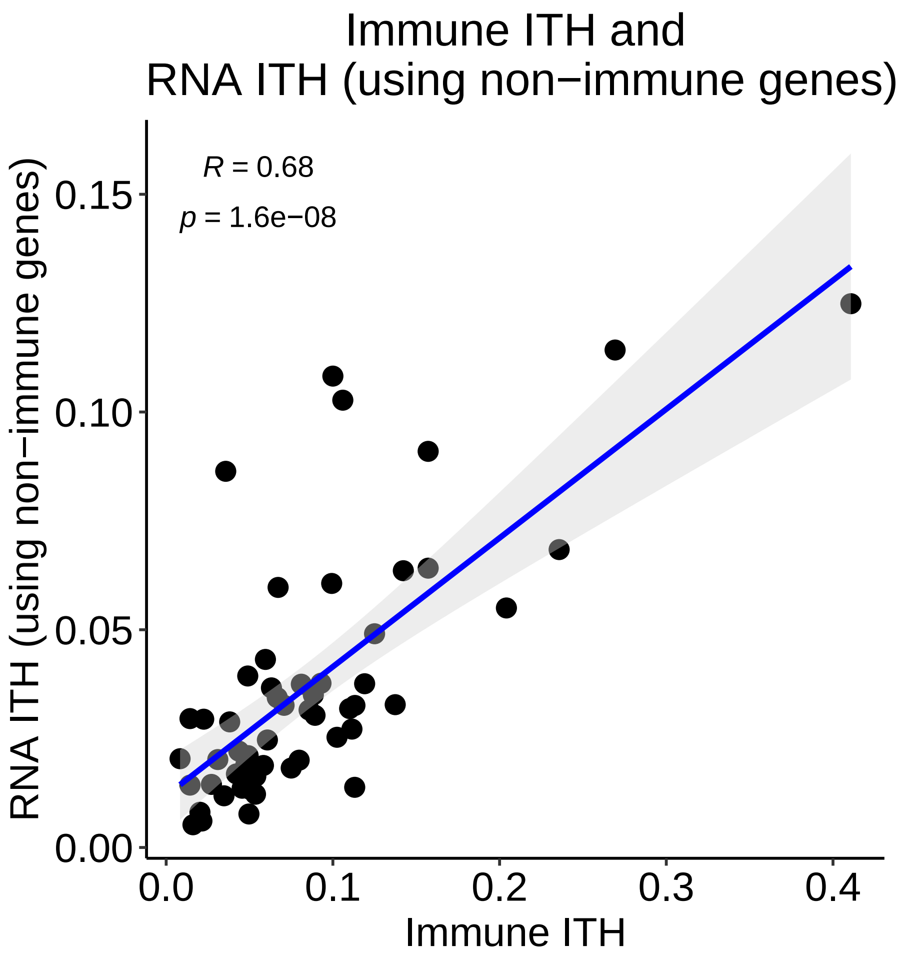

Supplementary Figure 11: **The relationship between immune ITH and RNA ITH (using non-immune genes).** RNA ITH is calculated using genes unrelated to immune function (see Methods). Immune ITH is calculated using RNA deconvolution of immune cells.

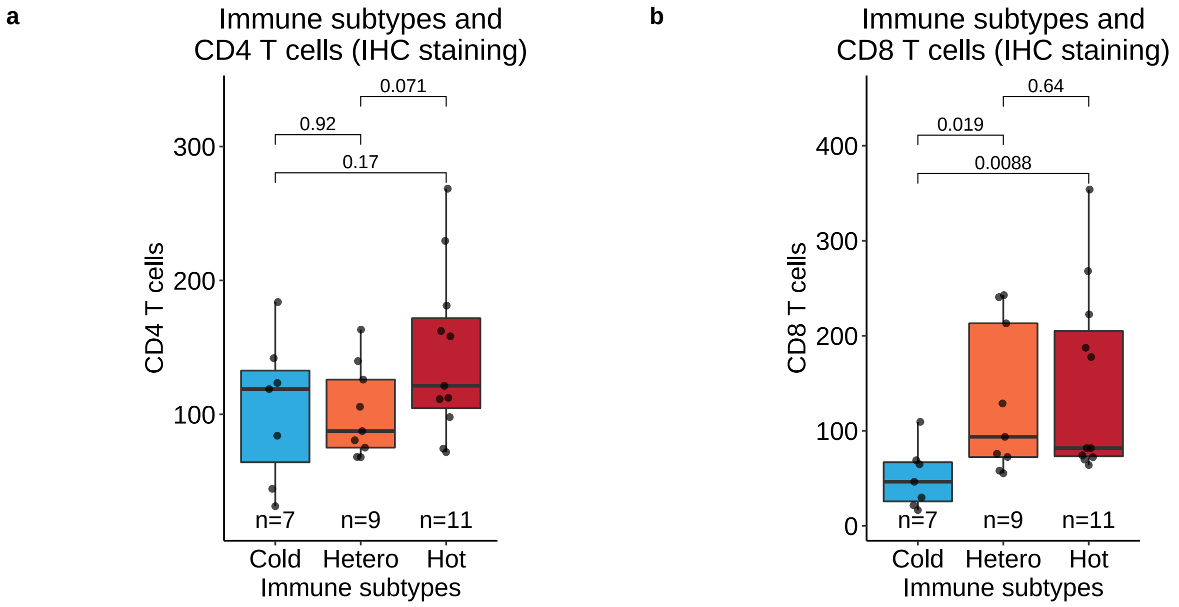

Supplementary Figure 12: **The level of CD4 and CD8 cells (IHC) in immunologically cold, heterogeneous and hot tumors** a) The level of CD4 cells (scored using IHC) in immunologically cold, heterogeneous and hot tumors (scored from RNA deconvolution), b) The level of CD8 cells (scored using IHC) in in immunologically cold, heterogeneous and hot tumors (scored from RNA deconvolution)

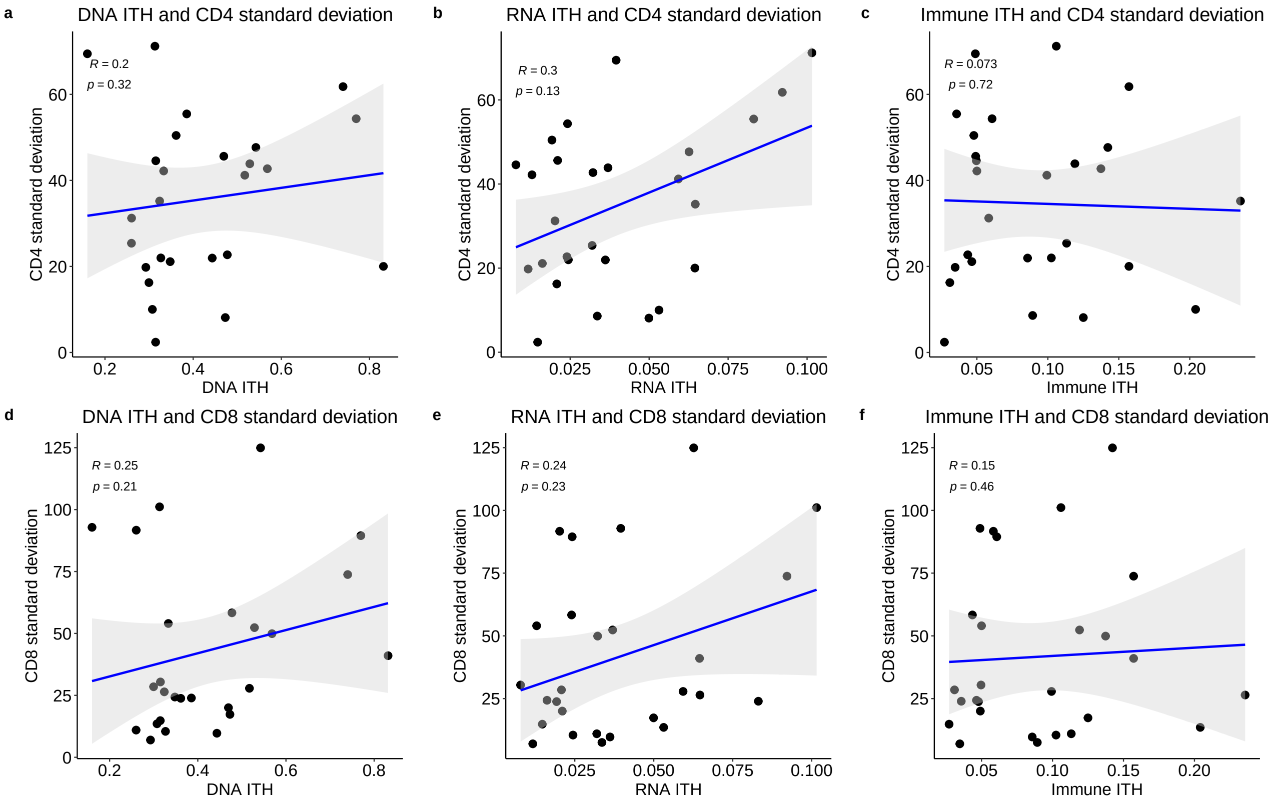

Supplementary Figure 13: **Immune heterogeneity scored using IHC and genomic ITH** a) Immune heterogeneity (scored using CD4 cells from IHC staining) and DNA ITH, b) Immune heterogeneity (scored using CD4 cells from IHC staining) and RNA ITH, c) Immune heterogeneity (scored using CD4 cells from IHC staining) and immune ITH (with RNA deconvolution), d) Immune heterogeneity (scored using CD8 cells from IHC staining) and DNA ITH, e) Immune heterogeneity (scored using CD8 cells from IHC staining) and RNA ITH, f) Immune heterogeneity (scored using CD8 cells from IHC staining) and immune ITH (with RNA deconvolution),

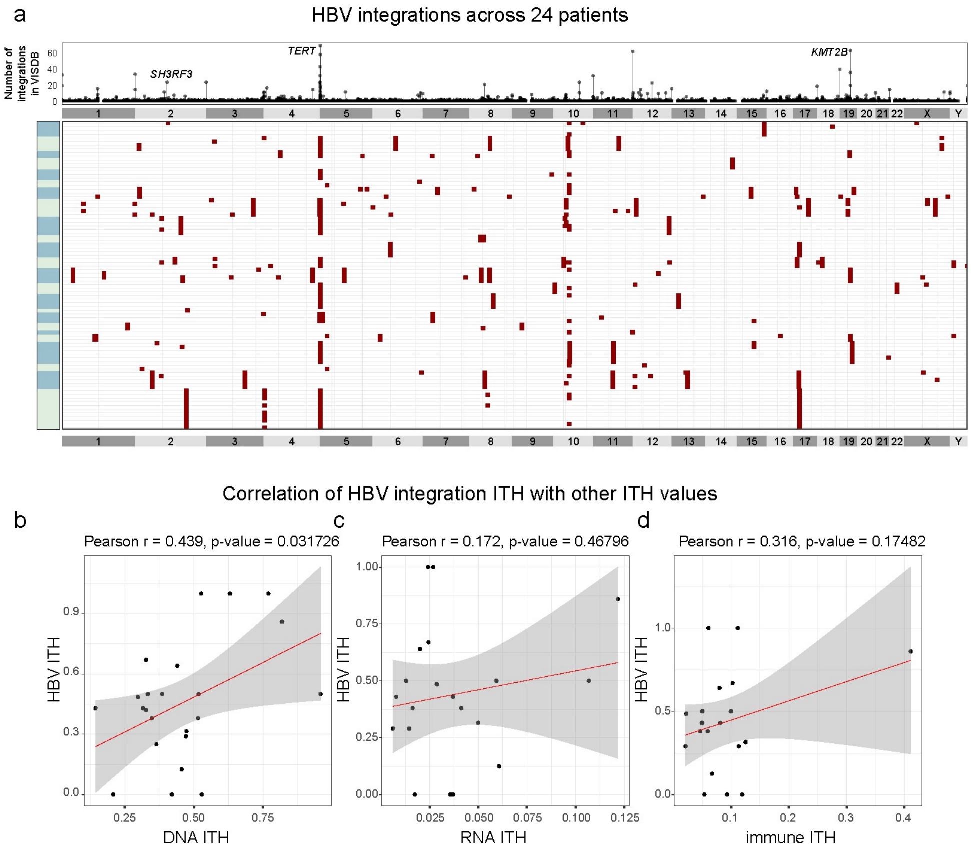

Supplementary Figure 14: **Viral integration across the PLANET cohort** a) Viral integration across 24 patients, b) DNA ITH vs HBV viral integration ITH, c) RNA ITH vs HBV viral integration ITH, d) Immune ITH vs HBV viral integration ITH

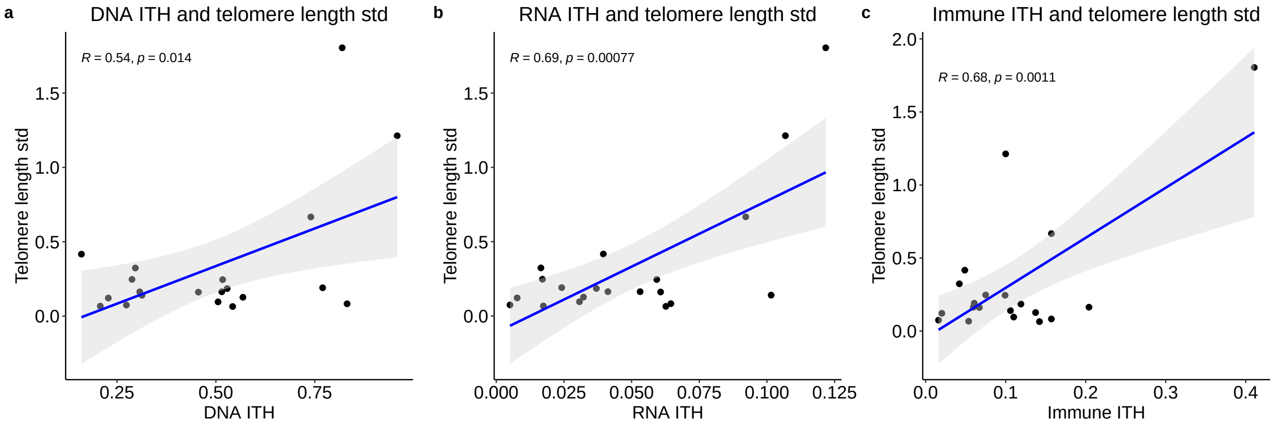

Supplementary Figure 15: **The correlation between telomere length ITH (standard deviation) and genomic ITH** a) The correlation between telomere length ITH (standard deviation) and DNA ITH, b) The correlation between telomere length ITH (standard deviation) and RNA ITH

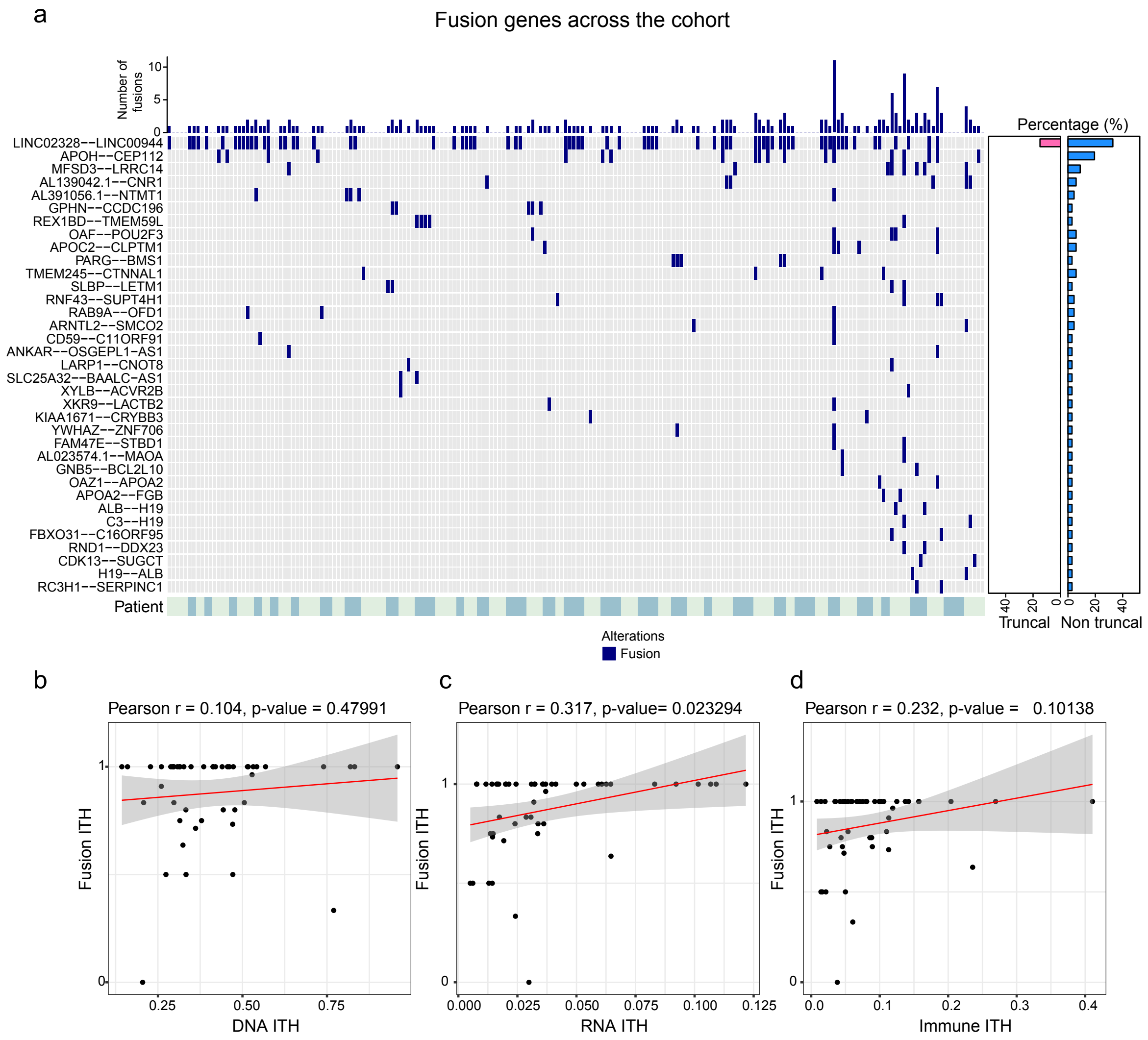

Supplementary Figure 16: **Fusion gene landscape across the PLANET cohort** a) Fusion gene landscape across patients. Only fusion genes which were identified in at least two patients are shown in the figure. Truncal and non-truncal fusion event percentages are shown as barplots (right). Number of total fusions are also shown as a barplot for each sample (top). Scatter plots showing associations between b) fusion ITH vs DNA ITH, c) fusion ITH vs RNA ITH, d) fusion ITH vs immune ITH.

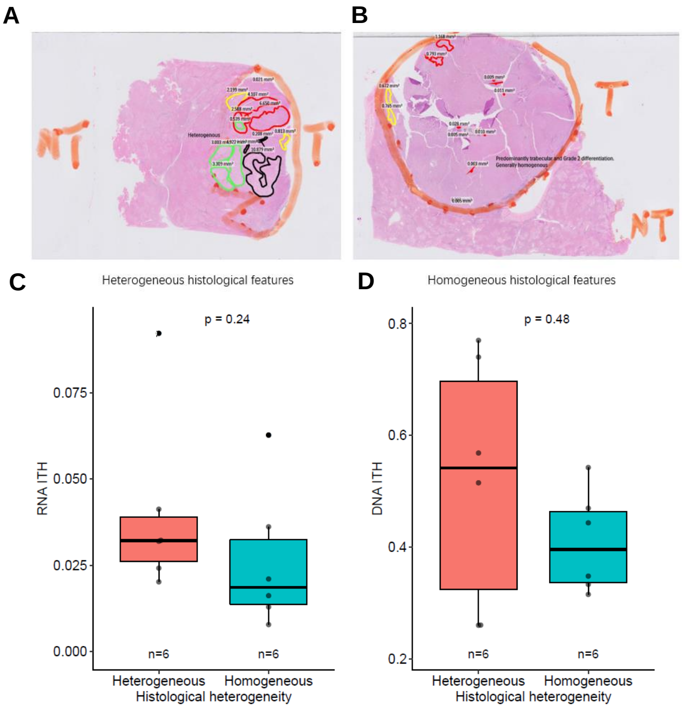

Supplementary Figure 17: **Histological heterogeneity and genomic ITH** a) Scoring of histological heterogeneity on H&E-stained section of patient tumour slides. Examples of a very heterogenous tumor (a) and a homogeneous tumor (b) are shown. Color schemes are: Red: Pseudoglandular, Yellow: Steatohepatitic / Steatosis, Green: Clear cell, Blue: Scirrhous, Black: Macrotrabecular / Chromophobe subtypes. c) Correlation between RNA ITH and histological heterogeneity (n=6), d) Correlation between DNA ITH and histological heterogeneity (n=6).

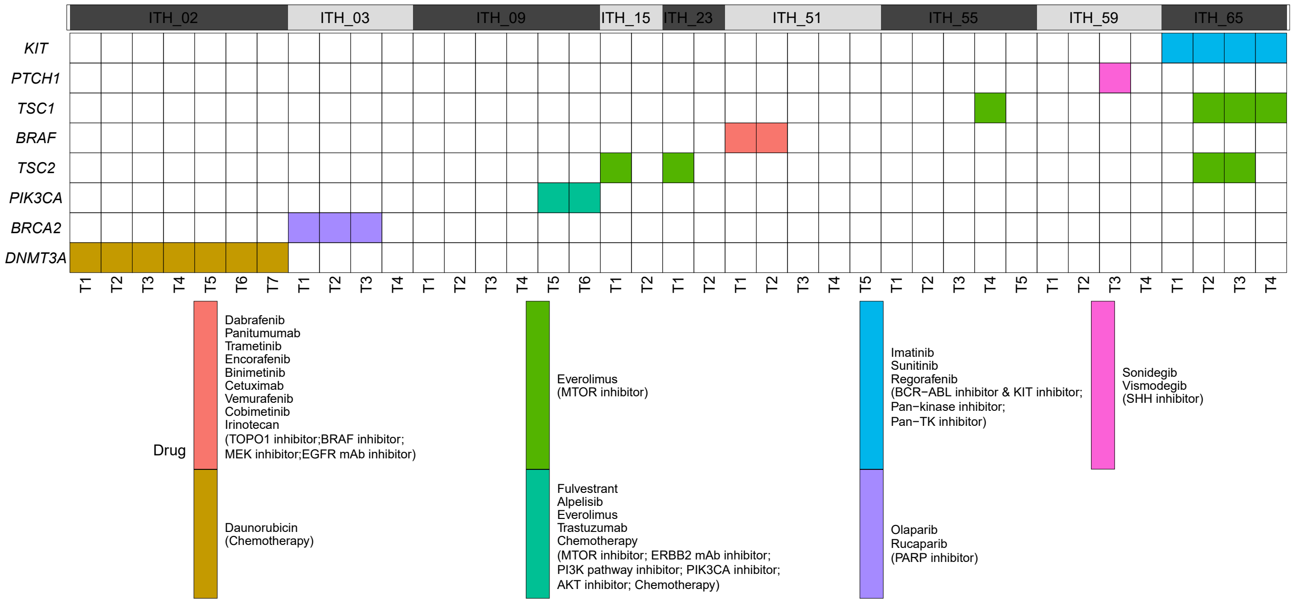

Supplementary Figure 18: **Druggable targets in the cohort** Heatmap shows sectors and patients (n=9) with mutations that are biomarkers in clinical guidelines (Level 1 mutations) according to CGI and OncoKB. Drugs for each mutation are shown in the figure legend. Out of the 12 different mutations (note that two different mutations cooccurred in TSC1 for patient ITH_65), only two were truncal (shared across all sectors from the same patient).

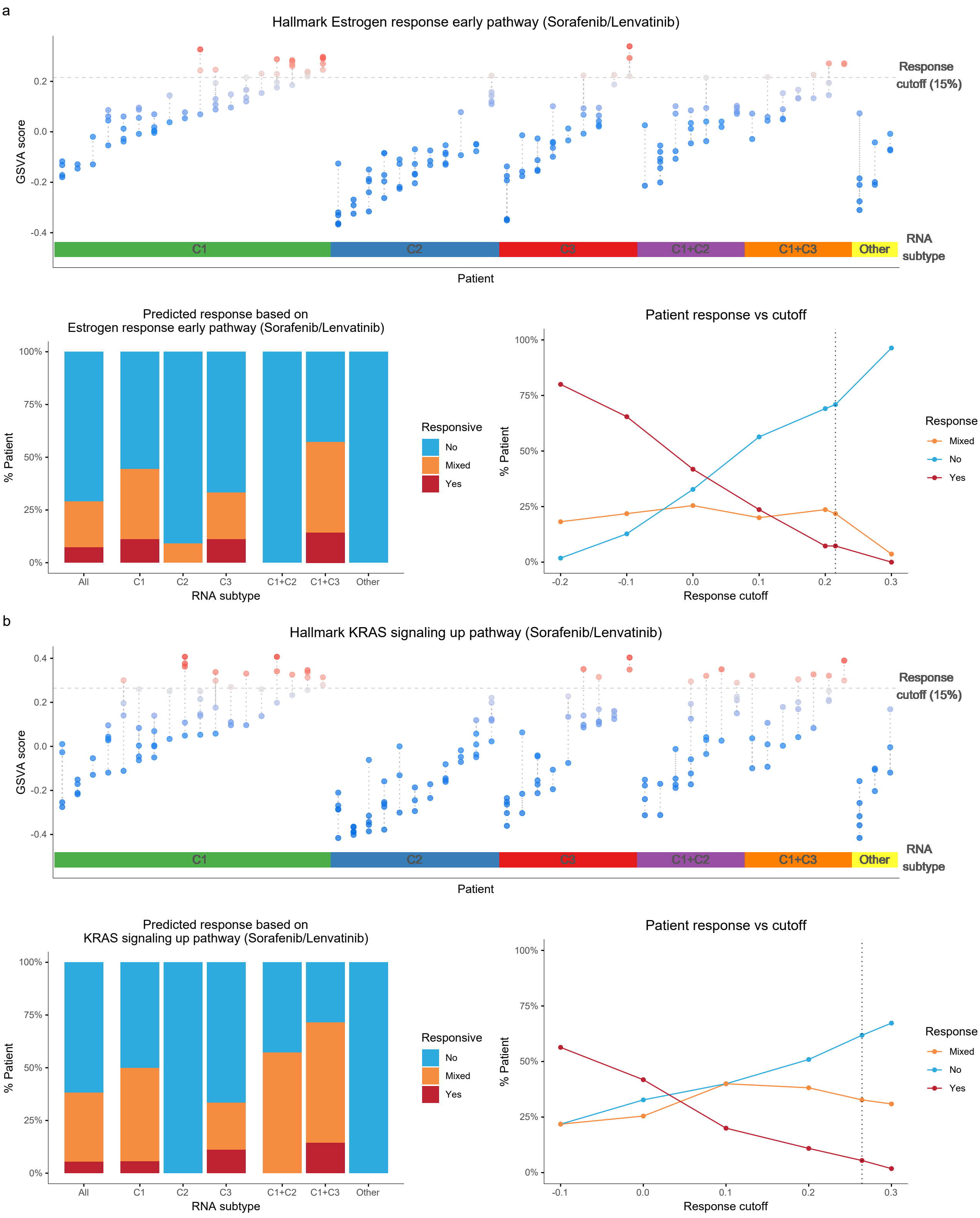
a
Hallmark Estrogen response early pathway (Sorafenib/Lenvatinib)
GSVA score
Response cutoff (15%)
C1
C2
C3
C1+C2
C1+C3
Other
RNA subtype
Patient
Predicted response based on Estrogen response early pathway (Sorafenib/Lenvatinib)
% Patient
Responsive
No
Mixed
Yes
All
RNA subtype
Patient response vs cutoff
Response
Response cutoff
b
Hallmark KRAS signaling up pathway (Sorafenib/Lenvatinib)
Predicted response based on KRAS signaling up pathway (Sorafenib/Lenvatinib)

c

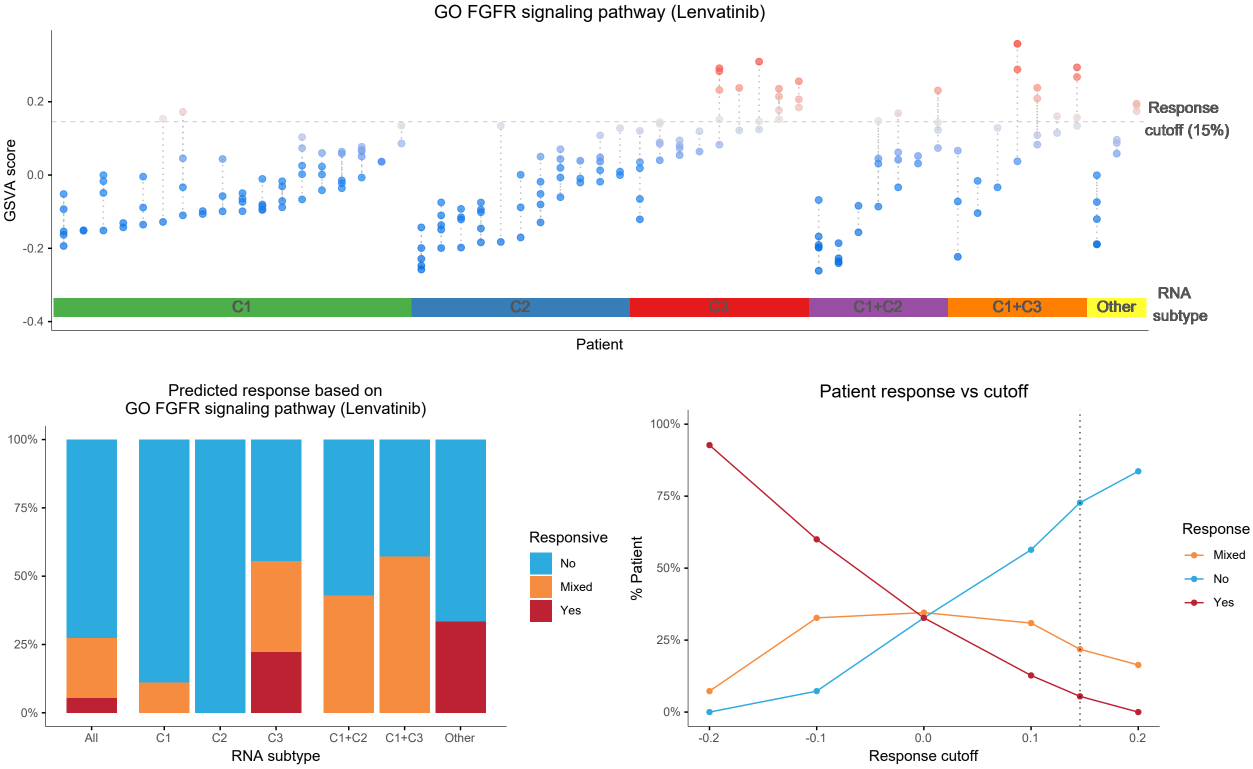

d

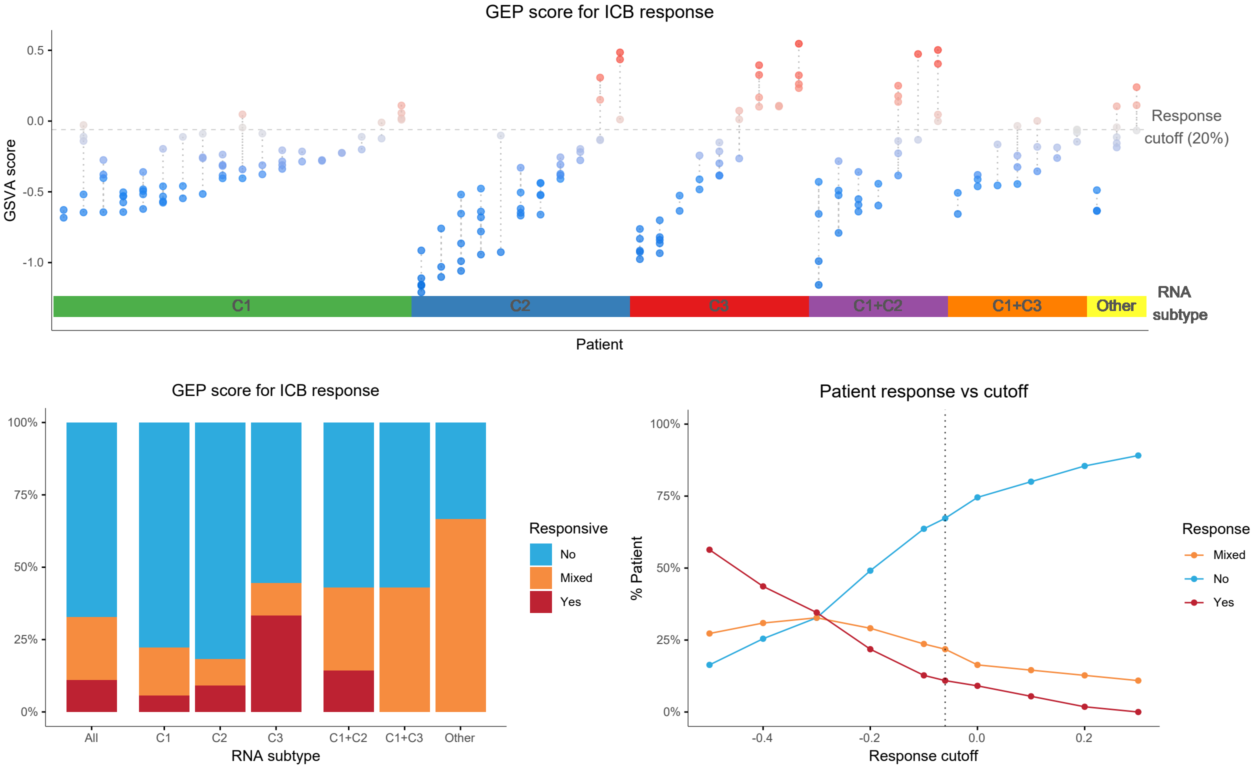

Supplementary Figure 19: **Impact of ITH in treatment** Related to Figure 5, in each panel, top plots show the pathway activation level with GSVA score or GEP score for ICB treatment response, while bottom left plots show the predicted response across patients based on cutoff values, and bottom right plots show the predicted response based on varying levels of cutoff values (Methods). Relevant drugs were indicated in panel titles. (a) hallmark estrogen response early pathway (Sorafenib/Lenvatinib), (b) Hallmark KRAS signaling up pathway (Sorafenib/Lenvatinib) (c) FGFR signaling pathway (Lenvatinib, based on Gene Ontology annotation of genes), (d) GEP score for ICB response

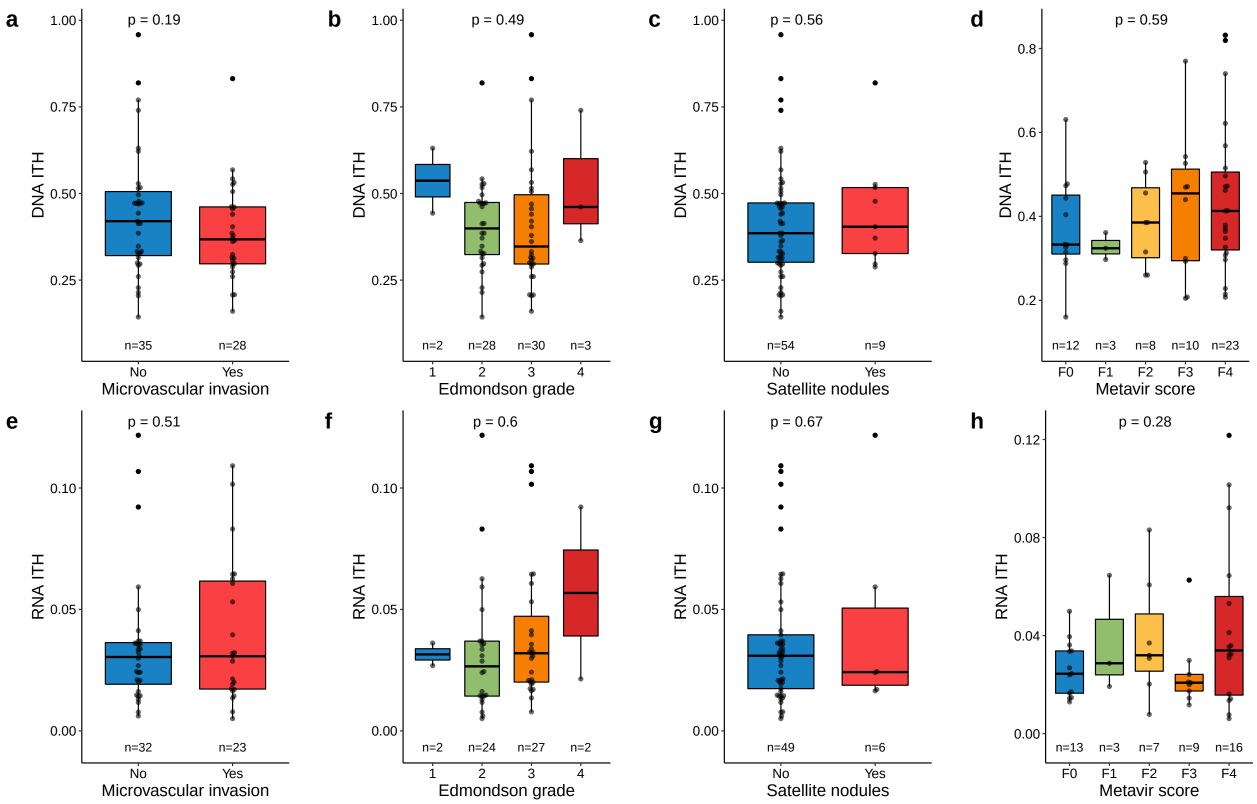

Supplementary Figure 20: **Histological levels and genomic heterogeneity** a) DNA ITH vs MVI, b) DNA ITH vs histological levels in differentiation (Edmondson grade), c) DNA ITH vs satellite nodule, d) DNA ITH vs histological levels in Metavir score, e) DNA ITH vs MVI, f) DNA ITH vs histological levels in differentiation (Edmondson grade), g) DNA ITH vs satellite nodule, h) DNA ITH vs histological levels in Metavir score

Variable		N	Hazard ratio	p−value
**Stage**	I	23	ref	
	II	16	12.97 (2.09, 80.37)	0.00591
	III	11	15.68 (2.73, 90.12)	0.00204
**Sex**	Female	13	ref	
	Male	37	0.87 (0.19, 3.92)	0.85560
**Age**		50	7.72 (2.05, 29.10)	0.00254
**Viral.status**	NBNC	19	ref	
	Hep B carrier	31	0.74 (0.17, 3.34)	0.69714
**AA.signature**		50	0.88 (0.27, 2.80)	0.82362
**Aflatoxin.signature**		50	2.18 (1.09, 4.37)	0.02796
**TP53**	non−mut	25	ref	
	mut	25	6.37 (1.61, 25.28)	0.00841
**CTNNB1**	non−mut	35	ref	
	mut	15	0.14 (0.01, 1.33)	0.08678
**TERT**	non−mut	37	ref	
	mut	13	0.48 (0.05, 4.87)	0.53296
**GD**	nGD	33	ref	
	GD	17	0.30 (0.08, 1.18)	0.08468
**RNA.subtype**	C1	16	ref	
	C2	11	35.54 (1.50, 839.98)	0.02692
	C3	8	69.35 (3.31, 1453.70)	0.00632
	mixed	15	207.32 (4.37, 9836.33)	0.00675
**DNA.ITH**		50	4.59 (1.30, 16.19)	0.01777
**RNA.ITH**		50	0.13 (0.03, 0.56)	0.00612
**Immune.ITH**		50	1.36 (0.62, 2.97)	0.43624
**Immune.subtype**	cold	18	ref	
	hot	17	0.02 (0.00, 0.20)	< 0.001
	mixed	15	0.16 (0.03, 0.99)	0.04839

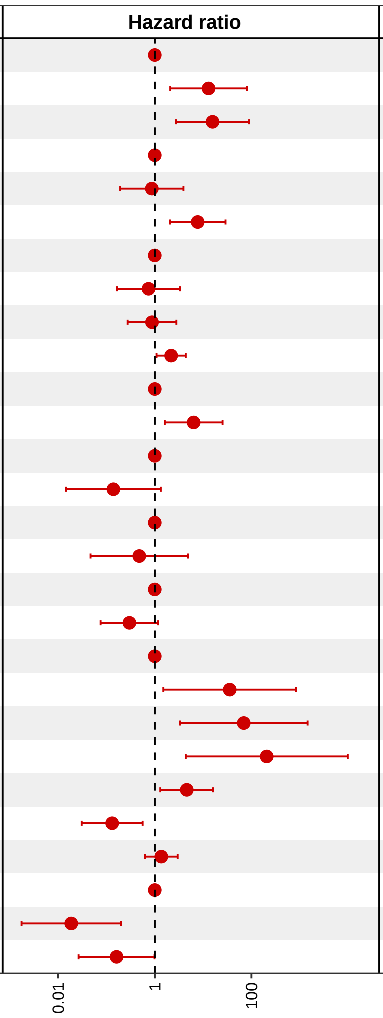

Supplementary Figure 21: **Forest plot for the full Cox model.** Related to Fig. 6b, levels for each features and numbers were shown. Hazard ratio as well as confidence intervals of hazard ratio were also plotted.

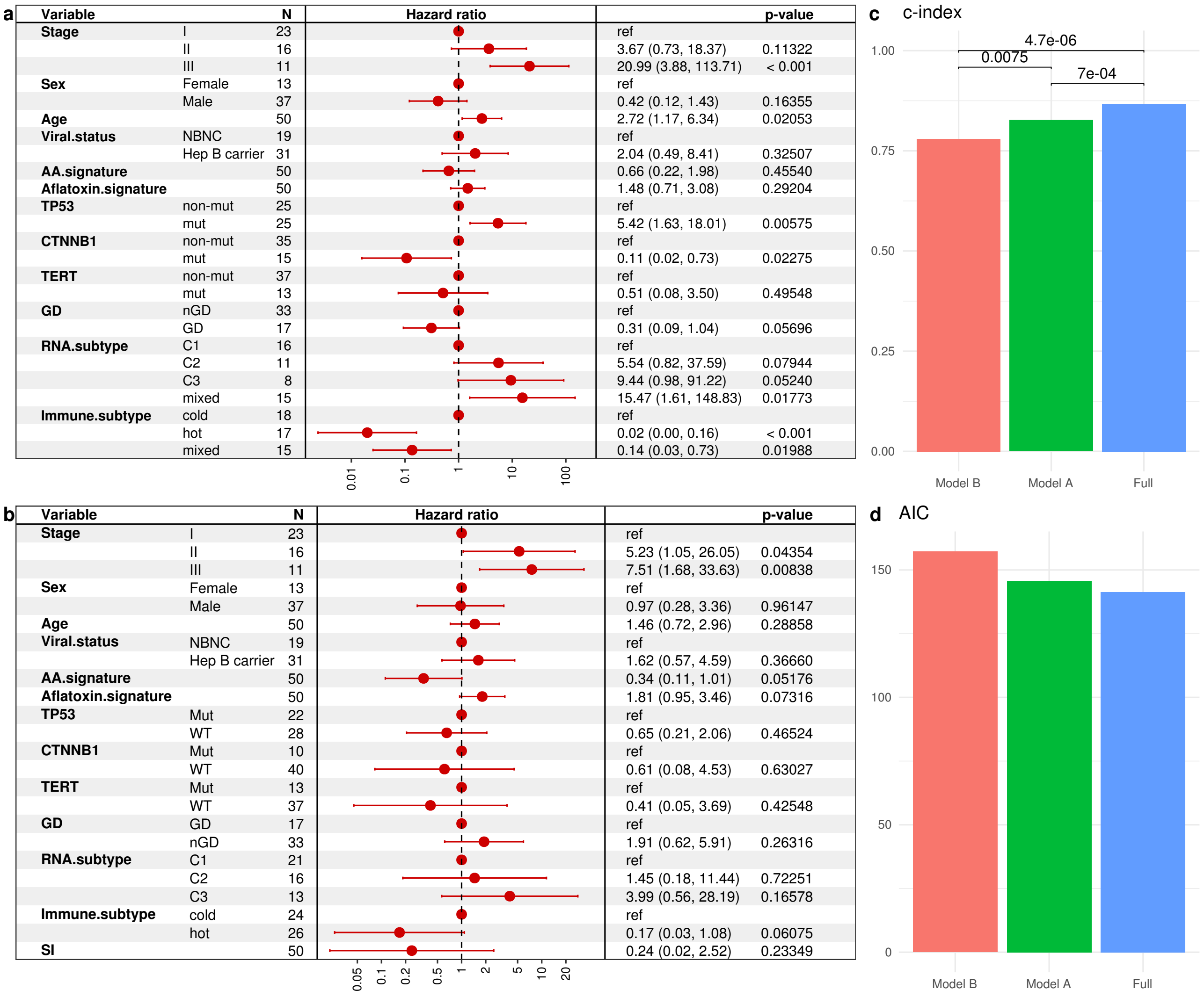

Supplementary Figure 22: **Statistical comparison between different survival models** a) Forest plot of the Cox model A (see Supplementary Note 5 for details). b) Forest plot of the Cox model B. c) d) c-index and AIC values of the full model, model A and model B.

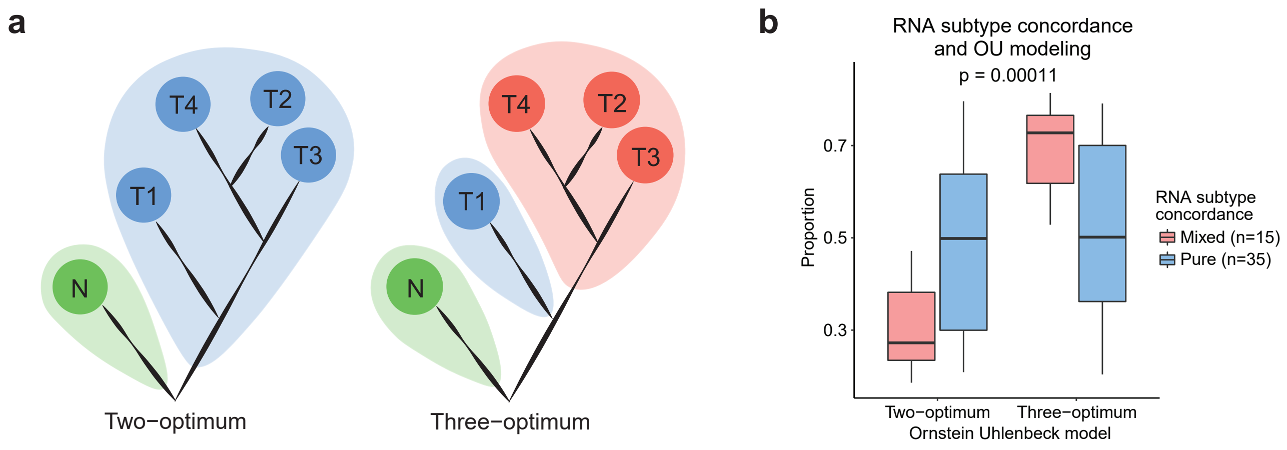

Supplementary Figure 23: **OU modeling of gene expression pattern** a) An illustration of a patient with two or three optima modeled using the OU process. In the latter, there are two optima within the tumor samples. b) Percentage of genes supporting the two or three-optima OU model in different patients.
