# Supplementary material for: Dynamic phenotypic heterogeneity and the evolution of multiple RNA subtypes in hepatocellular carcinoma: the PLANET study
Source: Natl Sci Rev. 2021 Oct 29;9(3):nwab192. doi: 10.1093/nsr/nwab192 (PMC8973408; doi:10.1093/nsr/nwab192)
Supplement: nwab192_Supplemental_Files [file nwab192_supplemental_files.zip › Supplementary_Methods_and_Notes.pdf]

Dynamic phenotypic heterogeneity and the evolution of multiple RNA subtypes in Hepatocellular Carcinoma: the PLANET study

Weiwei Zhai[1,2,21,25,26], Hannah Lai[1,25], Neslihan Arife Kaya[1,3,25], Jianbin Chen[1,25], Hechuan Yang[1,2,25], Bingxin Lu[1,4,25], Jia Qi Lim[1], Siming Ma[1], Sin Chi Chew[5], Khi Pin Chua[1], Jacob Josiah Santiago Alvarez[1], Pauline Jieqi Chen[1], Mei Mei Chang[1], Lingyan Wu[5], Brian K.P. Goh[6], Alexander Yaw-Fui Chung[6], Chung Yip Chan[6], Peng Chung Cheow[6], Ser Yee Lee[6], Juinn Huar Kam[6], Alfred Wei-Chieh Kow[7], Iyer Shridhar Ganpathi[7], Rawisak Chanwat[8], Jidapa Thammasiri[9], Boon Koon Yoong[10], Diana Bee-Lan Ong[10], Vanessa H. de Villa[11], Rouchelle D. Dela Cruz[12], Tracy Jiezhen Loh[13], Wei Keat Wan[13], Zeng Zeng[14], Anders Jacobsen Skanderup[1], Yin Huei Pang[15], Krishnakumar Madhavan[7], Tony Kiat-Hon Lim[13], Glenn Bonney[7], Wei Qiang Leow[13],Valerie Chew[16], Yock Young Dan[17], Wai Leong Tam[1,3,23,24], Han Chong Toh[18], Roger Sik-Yin Foo[1,19], Pierce Kah-Hoe Chow[1,5,6,20,22,26]

[1]Genome Institute of Singapore, Agency for Science, Technology and Research, Singapore 138672, Singapore.

[2]Key Laboratory of Zoological Systematics and Evolution, Institute of Zoology, Chinese Academy of Sciences, Beijing, China

[3] School of Biological Sciences, Nanyang Technological University, Singapore 637551, Singapore

[4] Cell & Developmental Biology, Division of Biosciences, Faculty of Life Sciences, Bloomsbury, London WC1E 6AP, UK

[5] Division of Surgery and Surgical Oncology, National Cancer Centre, Singapore 169610, Singapore.

[6] Department of Hepato-Pancreato-Biliary and Transplant Surgery, Singapore General Hospital, Singapore 169608, Singapore.

[7] Division of Hepatobiliary & Pancreatic Surgery, Department of Surgery, University Surgical Cluster, National University Health System, Singapore 119228, Singapore.

[8] Hepato-Pancreato-Biliary Surgery Unit, Department of Surgery, National Cancer Institute, Bangkok, Thailand.

[9] Division of Pathology, National Cancer Institute, Thailand.

[10] Department of Surgery, Faculty of Medicine, University of Malaya, Kuala Lumpur, Malaysia.

[11] Department of Surgery and Center for Liver Disease Management and Transplantation, The Medical City, Pasig City, Metro Manila, Philippines.

[12] Department of Laboratories, The Medical City, Pasig City, Metro Manila, Philippines.

[13] Department of Pathology, Singapore General Hospital, Singapore 169608, Singapore.

[14] Institute for Infocomm Research, A*STAR, Singapore 138632, Singapore

[15] Department of Pathology, National University Health System, Singapore 119228, Singapore.

[16] Translational Immunology Institute (TII), SingHealth Duke-NUS Academic Medical Centre, Singapore.

[17] Division of Gastroenterology and Hepatology, University Medicine Cluster, National University Hospital, Singapore.

[18]Division of Medical Oncology, National Cancer Center Singapore, 169610 Singapore, Singapore.

[19]Cardiovascular Research Institute, National University of Singapore, National University Healthcare System, Singapore 119228, Singapore.

[20] Singhealth-Duke-NUS Academic Surgery Program, Duke-NUS Graduate Medical School, Singapore 169857, Singapore.

[21]Center for Excellence in Animal Evolution and Genetics, Chinese Academy of Sciences, Kunming 650223, P.R. China

[22]Institute of Molecular and Cell Biology, Agency for Science, Technology and Research, Singapore 138673, Singapore.

[23]Department of Biochemistry, Yong Loo Lin School of Medicine, National University of Singapore, 8 Medical Drive, Singapore 117597

[24]Cancer Science Institute of Singapore, National University of Singapore, 14 Medical Drive, Singapore 117599

[25] These authors contributed equally

[26]Correspondence should be addressed to pierce.chow.k.h@singhealth.com.sg and weiweizhai@ioz.ac.cn

Supplementary Methods

Patient recruitment and grid sampling

67 patients were recruited from several regional hospitals from the Asia-Pacific Hepatocellular Carcinoma (AHCC) trial group. In particular, 59 patients were recruited under a Translational and Clinical Research (TCR) Flagship Programme: Precision medicine in Liver cancer across an Asia-pacific NETwork (PLANET, NCT03267641, https://clinicaltrials.gov/ct2/show/NCT03267641), funded by the Singapore National Medical Research Council (NMRC) program (Supplementary Table 1). PLANET program involved multiple hospitals in Singapore (National Cancer Centre Singapore, Singapore General Hospital, and National University Hospital), Malaysia (University Malaya Medical Centre), Thailand (National Cancer Institute-Thailand) and Philippines (Medical City). Patient with early stage liver cancer based on AASLD imaging criteria were recruited. PLANET recruitment required the patients to have no extra-hepatic metastasis (defined as lymph node <2 cm, lung modules < 1 cm, farther lymph nodes < 2 cm) with R0 or R1 resection and Child-Pugh ≤ 7 points without clinical ascites. A full set of patient recruitment criteria is described in the Supplementary Note 1. This study has been approved by Singhealth Centralized Institutional Review Board (2016/2626 and 2018/2112). Informed consent was taken from each patient before enrollment.

Tissue extraction and library preparation

Within the PLANET program, tissue resection followed similar procedures as our previous study [1]. Resected surgical specimens were retrieved from the surgical field in the operating room and transported on ice in a temperature-controlled cooler container to a pathologist. A single slice was harvested in the tumor through the capsule and the slice was reviewed for necrosis, fibrosis, haemorrhage and cystic changes. Multiple sectors (regions) along one axis of the tumor were then harvested, often ≥ 1 cm apart and cut into pieces measuring 1cm x 0.5cm x 0.5 cm. Normal non-tumor liver tissue (≥ 2 cm away) from the tumor was also harvested. Circumstances such as necrosis would reduce the number of tumor sectors harvested and the numbers of sectors harvested depend on the size of the tumors.

Genomic DNA and mRNA were extracted from the normal and tumor tissues using Qiagen AllPrep DNA/RNA Mini Kit. Quality check of the extracted DNA and RNA were conducted using gel electrophoresis and Agilent 2100 Bioanalyzer respectively. Qualified genomic DNA were shortened by sonication using the Covaris system and quality check of the fragments were performed with Agilent 2100 Bioanalyzer. The fragments were end-repaired, adaptor-ligated, amplified and sequenced by Novogene-AIT Inc. For WES, NimbleGen SeqCap EZ Human Exome Library v3.0 was used for exome capture before

sequencing[1]. mRNA-seq libraries were prepared through a series of processes including poly-A selection, reverse-transcription, fragmentation, end-repair and adaptor-ligation. The final mRNA libraries were sequenced at Genome Institute of Singapore.

Somatic mutation calling

Raw paired end read were mapped to human reference genome (GRCh37) using Burrows-Wheeler Aligner (BWA) (version 0.7.12) [2]. Duplicated reads were removed using sambamba (version 0.6.4) [3] and base quality recalibration and local realignment were conducted using the Genome Analysis Toolkit (GATK, version 3.1-1). For the two WES patients with higher than average sequencing depth, their bam files were down-sampled to match the mean sequencing depth of WGS samples. Somatic point mutations were called using Mutect (1.1.7) algorithm by comparing normal and tumor samples [4]. Strelka (version 1.0.14) was used for indel calling [5].

***De novo* signature analysis and timing of signatures**

De novo mutation signatures were inferred using BayesNMF [6]. Optimal number of signatures was found to be 17 based on 50 independent iterations and mutations were decomposed to these *de novo* signatures using deconstructSigs [7]. After filtering out the signatures with low frequency (those with mean proportion across samples < 0.02 or maximum proportion across all the samples < 0.2), we obtained 14 *de novo* mutational signatures. These 14 signatures were subsequently matched to 17 known COSMIC signatures (cosine similarity >0.7) [8,9], where 13 of them were used for the downstream analysis after filtering out the low frequency signatures using the same criteria as described above. Mutations were then decomposed to these 13 known signatures using R program deconstructSigs. In order to understand time dependent changes in the mutational process, we partitioned the mutations into truncal (shared across all sectors) and non-truncal events. Signature contributions (i.e proportions) were inferred separately for the truncal and non-truncal mutations.

Driver gene identification

Somatic mutation data from four biggest HCC cohorts including a Japanese cohort(n=514) [10], a French cohort from ICGC (n=242) [11], a US cohort from the TCGA (n=373) [12] and a Korean cohort (n=231) [13] were complied. In total we used, 7,242 indels and 129,292 somatic point mutations from coding regions of the genome. Somatic mutations were annotated using the Oncotator (Version 1.9.2.0) [14]. To capture different features of driver genes, three driver identification algorithms including MutSigCV [15], TUSON Explorer [16] and 20/20+ [17] were used. In total, 62 candidate driver genes were identified by combining drivers from three different algorithms (q-value <0.1, Supplementary Note 3).

Copy number analysis

Somatic copy number variations were identified using Sequenza (version 2.1.2) [18] and segmental copy numbers were converted to arm and cytoband level CNVs using the GISTIC algorithm [19]. In order to compare CNVs in our cohort with the public cohort, GISTIC was used to identify significantly amplified or deleted regions in the TCGA cohort (TCGA-LIHC). Genome wide SNP array level 3 data (copy number segmental information) was downloaded from the GDC Data portal. In order to find arm level events, ploidy adjusted copy number segments were calculated by subtracting ploidy from total copy number. Subsequently, cytoband annotation were performed using copy number positions. For each chromosome arm, segment lengths were summed if they have copy number event in the same direction. Chromosome arms were determined as amplified or deleted if at least 70% of the arm has alteration in the same direction. To find subclonal and clonal CNVs, significantly altered cytobands (q value<0.01) were first identified from the TCGA cohort. Subsequently, amplification and deletion events were identified in our samples using "all_thresholded_by_genes.txt" output from the GISTIC results. If all samples have the same CNV event for a cytoband, it was called as a clonal CNV. If the CNV event occurs to a subset of sectors, this event will be called as a subclonal event. In order to annotate important driver genes in the cytobands, we combined genes from the Cancer Gene Census genes (CGC, n=723), liver driver genes (n=62) and a subset of driver genes that were reported in a few large-scale HCC studies [20].

DNA ITH and missing variability

We measured the level of tumor heterogeneity in DNA (DNA ITH) as the number of private mutations divided by the total number of mutations (Fig. 2a). Since the proportion of shared mutations were affected by the number of sectors, for patients with more than 2 sectors, we calculated DNA ITH for all combinations of sample pairs and computed the mean value. To measure the amount of increase in variability (i.e. number of somatic mutations) as a function of number of sectors, we calculated the fold increase as M_x/M_1, where M_i is the average number of detected somatic mutations when we randomly sample i sectors from the total sample list. For exome sequencing data, we downsampled the coverages to the mean coverage of the WGS and recalculated the DNA ITH based on mutations called with the downsampled data.

Spatially mixing (SM) and spatially separated (SS) tree patterns

For any three sectors sampled from a tumor, we tested whether the phylogenetic relationship matched the physical locations of the tumor sectors (spatially separated (SS)) or not (spatially mixed (SM)), Fig. 2c). For each of the 49 patients with at least 3 tumor samples, we took all possible combinations of 3 tumor

samples and computed the proportion of trees with SS pattern. K-mean clustering was applied to classify patients' trees into SS and SM patterns.

Phylogenetic reconstruction, Fixation index (FST) and isolation-by-distance (IBD)

Using the list of somatic mutations called from each sample, we calculated the hamming distance between all sample pairs and inferred the phylogenetic relationship between tumor samples using the Neighbor-joining algorithm [21]. FST measures the amount of population differentiation between samples taken from different populations. We used the unbiased estimator from Weir and Cockerham 1984 [22]. In order to explore the relationship between the physical distance and genetic divergence (i.e FST), we applied the linear model between FST values and the physical distance between the sectors. A positive and significant linear relationship indicates an isolation-by-distance pattern (IBD) observed among tumor sectors.

Clonal decomposition using Pyclone and PhyloWGS

Two methods: PyClone [23] (version 0.13.1) and PhyloWGS [24] were used for the clonal decomposition. Due to the computational constraint, 500 somatic variants were randomly selected across the whole genome for PyClone and PhyloWGS. For PyClone, copy number variations (CNVs) were obtained from the output of Sequenza. For PhyloWGS, CNVs were estimated using TitanCNA [25]. PyClone and PhyloWGS predicted number of clones, clonal structure, mutation assignment and mutation VAF distribution in each clone as outputs. PhyloWGS also estimated optimal clonal phylogeny for each patient, while CITUP [26] was used to find the optimal clonal phylogeny from the PyClone output. Clonal phylogenies were visualized using ggplot2 package in R. For a patient, PyClone and PhyloWGS gave the clonal prevalence of clones in tumor sectors and cosine distances between vectors of clonal prevalence of all pairwise tumor sectors were computed. At a physical distance, cosine distances of all pairwise sectors with the same physical distance were used to draw a boxplot and a linear model was subsequently fitted between the physical distance and their cosine distance in the clonal composition across sectors.

Site frequency spectrum (SFS) and neutrality

We computed cancer cell fraction (CCF) of all mutations adjusting tumor purity and copy number using the method provided in R package EstimateClonality (available on https://bitbucket.org/nmcgranahan/pancancerclonality/src/master/) [27]. Under the neutral model presented in Williams et al. [28], the number of subclonal mutations detected in the tumor should accumulate linearly with the inverse of their VAF. Thus, neutrality was measured as the goodness-of-fit between 1/VAF and number of cumulative mutations (i.e. R-square of the linear regression). In our neutrality analysis, only tumor

sectors with purity of ≥ 50% were included, and adjusted VAF values were used to compute R-square statistic.

RNA clustering and homologous subgroups across cohorts (submap)

198 tumor samples from 55 patients were used to identify molecular subtypes of HCC in this cohort. Short reads from RNA samples were mapped to the reference genome using STAR [29]. Subsequently, RSEM [30] was used to quantify the level of gene expression for each gene (available at https://github.com/gis-rpd/pipelines). We normalized the RSEM raw count using DESeq2 [31] and performed log2 transformation on the normalized counts to stabilize variance of all genes. We then selected the top 3000 most variable coding genes based on their median absolute deviation (MAD) across the cohort. To ensure robustness of RNA subtype, we used NMF clustering and bootstrapping to assign subtypes to samples of each patient [32]. For each of 1000 bootstrap samples, 3000 genes were randomly selected from the top 3000 MAD genes with replacement and NMF clustering was performed on all RNA samples using the resampled genes. We counted how many times out of 1000 bootstraps the patients have mixed RNA subtypes. 17 patients had mixed RNA subtypes in more than 700 (70%) bootstraps and were classified as mixed RNA subtype. The other 38 patients were classified as pure RNA subtype. To identify common subgroups between different datasets, an unsupervised subclass mapping method was used with the SubMap module (http://software.broadinstitute.org/cancer/software/genepattern/) [33] and p-values were adjusted using the Bonferroni multiple test correction procedure.

Gene set enrichment analysis and RNA ITH

Gene set enrichment analysis was carried out using GSVA[34] package in R. We utilized Hallmark [35], Reactome [36], and liver-related chemical and genetic perturbations (CGP) gene sets in our analysis. CGP pathways were curated from several publications and are also available on GSVA website (https://www.gsea-msigdb.org/) [37-39]. For each patient, Spearman distances (1-Spearman correlation) between coding gene expression vectors of all pairwise tumor samples were computed. Mean of all pairwise sector distances was presented as RNA ITH value of the patient. In order to calculate RNA ITH using genes unrelated to immune function, we used AmiGO (http://amigo.geneontology.org/) to filter for immune genes[40]. Genes annotated under the biological process of "immune system process" were defined as immune genes (n=3,276). For each patient, Spearman distances between non-immune gene expression vectors of all pairwise tumor samples were computed. Mean of all pairwise sector distances was presented as non-immune RNA ITH value of the patient. For genes correlated with tumor purity, we correlated tumor purity with gene expression levels using the Spearman correlation test. After the multiple test correction with the

Benjamini and Hochberg method, genes correlated with tumor purity were extracted based on the adjust p-value of 0.05.

Fusion gene identification and fusion gene ITH

FusionCatcher (v1.33)[41] was used to identify fusion genes using raw RNA-seq fastq files. Fusion events were further filtered if; 1) found in normal samples, 2) annotated as 'probably false positive' by FusionCatcher, 3) the number of commonly mapping reads is greater than 10, 4) the number of spanning reads is less than 3. Only known fusions were used for the analysis. Fusion ITH was calculated as the ratio of non-truncal fusions to the total number of fusions identified in a patient.

Modeling gene expression using the Ornstein-Uhlenbeck (OU) process

To understand the evolutionary mode of gene expression changes within a tumor, especially how this change might lead to the discordance of RNA subtypes across tumor samples, we employed the Ornstein Uhlenbeck (OU) process to model gene expression changes across tumor sectors using the ouch package implemented in R[42]. Using expression of each gene as a trait, we modeled how each trait evolves along a patient's DNA tree. We only applied the OU process to 50 patients with at least two tumor samples with both RNA and DNA data (35 patients with concordant and 15 patients with discordant samples). We tested whether there should there were multiple optima (corresponding to different RNA subtypes) within the tumor samples (Fig. 3K). In the baseline model, gene expression evolved as an OU process with two optima (tumor and normal samples). In the advanced model, there were two optimums within tumor samples, i.e. an OU process with three optima (one for the normal and two optima for tumor samples). For each patient, the two models were fitted for each coding gene on their DNA tree. Specifically, for discordant patients, in the OU process with three optima, three populations consisted of one with only the normal sample, and two with tumor samples belonging to two different subtypes. While for concordant patients, their tumor samples were randomly assigned to two groups which corresponded to two subpopulations. Using Akaike information criterion (AIC), the most fitted model was chosen for each gene from each patient. We computed the percentage of genes that had most fitted model as OU process with two optima or three optima.

Immune subtypes, immune ITH and immune scores

Immune cell populations of tumor samples with available RNA-seq data were estimated using the method of Danaher et al. [43]. There were 15 estimated immune cell populations: CD8+ T cells (CD8), exhausted CD8+ T cells (CD8 exhausted), regulatory T cells (Treg), helper T cells (TH1), dendritic cells (dendritic), B cells (B cell), mast cells (mast), natural killer cells (NK), natural killer CD56− cells (NK CD56−),

neutrophils, macrophages, CD45+ cells (CD45), total T cells (T cells), total TILs (total TIL) and cytotoxic cells (cyto). Using these immune-infiltrate estimates, k-means clustering (k=2) was applied to identify two immune subtypes (i.e. hot and cold). To calculate immune ITH of each patient, Spearman distance of immune-infiltrate estimates between each pair of tumor sectors was computed and the mean of all pairwise distances was taken as immune ITH of the patient. GEP scores were calculated using the Ayers et al. [44] method. The Jiang et al. [45] immune scores were estimated using the TIDE web interface (http://tide.dfci.harvard.edu/).

Immunohistochemistry (IHC) and histological subtypes

IHC on 16 patients' tissues was performed as previously described with anti-human CD4 (Abcam, clone EPR6855, 1:200) and CD8 (DAKO, clone C8/144B, 1:200) antibodies [46]. The density of CD4+ and CD8+ T cells was quantified as number of cells/mm^2 from ten 3mm^2 representative fields. We then calculated the mean of cell density across tumor regions, which reflects the level of infiltration for each tumor. Representative H&E-stained sections of a subset of the tumours (n=12) were also taken by our hepato-pathologists and were annotated for various histological features. Based on the relative comparison of these tumours, we divided the tumours into 'histological heterogenous' and 'histological homogenous' categories (Supplementary Figure 17).

HBV integration and telomere length

After quality control including removing patient samples with low tumor purity (purity <0.3), BatVI (v1.02) [47] used for detecting viral integration in 177 WGS samples from 37 HBV positive patients. Read counts within 500bp for the identified integrations were merged and integrations with only one supporting read were filtered away from the integration sites. In order to calculate the heterogeneity of viral integrations, integration sites within 500bp were treated as the same integration. ITH level of HBV integrations for a patient is calculated as the mean proportion of private integrations when we randomly sample two samples from the tumor sectors. Telomere length was estimated using the TelSeq method (version 0.0.1)[48]. We set the threshold of the amount of TTAGGG/CCCTAA repeats in a read to be considered telomeric to be 7. Standard deviation of the telomere length across sectors was used as the telomere length ITH of the patient.

Drug target annotation and treatment response

All mutations were annotated using CGI[49] and OncoKB[50]. From CGI, each druggable mutation was classified into four categories: a) in clinical guidelines; b) used in clinical trials; c) supported by clinical case studies; and d) supported by pre-clinical studies. From OncoKB (levels of evidence V1), each

druggable mutation was classified into 4 large categories: a) recognized by FDA; b) standard of care; c) supported by compelling clinical evidence; and d) supported by non-clinical evidence. Combining these categories, we classified druggable mutations into four levels: 1) in clinical guidelines (CGI a, OncoKB a and b); 2) biomarkers supported by clinical data (CGI b and c, OncoKB c); 3) Biomarkers supported by pre-clinical data (CGI d and OncoKB d); and 4) mutations that were not the exact match of known biomarkers, but occur in the target genes or in the gene list of the widely used FoundationOne CDx genomic test. Only the highest level of each mutation was used. To investigate the clonality of these potential targets, druggable mutations in each level were defined as truncal if they occurred in all sectors, and as non-truncal otherwise. To demonstrate the increased proportion of patients found with therapeutic targets with the increased number of sampling, different numbers of sectors (from 1-5) were randomly sampled with replacement from the primary tumor of a patient to examine if any therapeutic target at a given level exists. The mean proportion of patients that harbor therapeutic targets and the standard deviation (error bar) were reported from 50 random sampling (Fig. 5c).

To explore the ITH in expression for the target pathways of sorafenib and lenvatinib [51-53], we investigated the activation level of the targeted pathways using GSVA[34]. As a proof of principle, we assumed that for tumor sectors that are potentially responsive to a targeted therapy, the activation level of the targeted pathway would be above a certain level, which can be approximated based on the reported drug response rate. For angiogenesis pathway, the cutoff was set to match a 15% response rate across all samples [54,55]; for immunotherapy (i.e. GEP score), the cutoff was set to match 20% response rate [56,57]; for the rest of the candidate pathways, 15% response rate was assumed for demonstration purpose. Based on the above cutoff settings, patients were then classified into fully responsive, fully non-responsive and mixed based on the proportion of sectors above the cutoff value. To demonstrate the possible effect of different thresholds, we also varied the cutoffs across possible the GSVA scores (Fig. 5f, Supplementary Fig. 19).

Feature correlation and integrative survival analysis

From the patient cohort, we curated several important clinical (stage, sex, age, viral status), molecular (AA signature, aflatoxin signature, mutation status of driver mutations at TP53/ CTNNB1/TERT, RNA subtypes, genome doubling, immune subtypes), and ITH features (DNA ITH, RNA ITH and immune ITH). For testing correlation among variables, Fisher's exact test was used for two categorical variables. Linear regression was used for two continuous variables and Kruskal–Wallis analysis of variance test was applied in case of mixed categorical and continuous variables.

For a prospective cohort, we used the patient relapse free survival integrating all 15 variables in the model (using the coxph function in R). In order to compare importance of the variables, likelihood ratio test was

applied to the model with and without each variable across all variables. Likelihood ratio Chi Square value is used as the indicator of importance using Anova function in R. Importance was calculated as the percentage of each variable over the sum of all chi-square values. Using the multivariate cox model, we divided patients to three groups based on the predicted hazard values. Kaplan-Meier survival curves of three survival groups were plotted using the surv_fit function in R. To compare performance of the model with and without ITH features, Harrell's concordance index (c-index) was calculated using concordance.index function from *survcomp* R package. For DNA ITH in single sector sampling (model B in Supplementary Note 5), clonal frequencies from the selected sector calculated from Pyclone were used to calculate the Shannon index by using the formular: $SI = -\sum_{i=1}^{Num.Clone} p_i \ln p_i$, where p_i is the frequency of clone i.

Supplementary Note 1: patient cohort and the inclusion and exclusion criteria for the PLANET study

PLANET was set-up to study the impact of ITH on the clinical trajectory of resected HCC (NCT03267641). The recruitment criteria for PLANET are：

Inclusion Criteria

1. Male and female patients, 21 to 90 years of age at the time of signature of the informed consent form.
2. Clinically AND histologically proven HCC or hepatocellular-cholangiocarcinoma after liver resection.
3. HCC or hepatocellular-cholangiocarcinoma limited to the liver with no extra-hepatic metastasis on CT or MRI of the abdomen and chest (defined as lymph node <2 cm, lung modules < 1 cm, further lymph nodes < 2 cm) according to the AASLD criteria.
4. R0 or R1 resection on histology.
5. Eligibility according to tumour size based on pre-op imaging:
    a. Large tumour >= 5 cm (preferred)
    b. Smaller tumours >=2cm and < 5cm
    c. Multifocal tumours - maximum of 3 total tumours with at least one with size >=2cm detected from CT-scan.
6. Child-Pugh ≤ 7 points without clinical ascites before surgery.
7. ECOG performance status 0-1 before surgery.
8. Scheduled for liver resection within 6 weeks.
9. The patient has received no anti-cancer specific treatment for HCC or hepatocellular-cholangiocarcinoma eg. previous liver resection, loco-regional therapy (e.g. RFA, TACE,

SIRT), radiotherapy, immunotherapy, chemotherapy or neo-adjuvant chemotherapy other than the planned surgery. However, patient who has received previous HCC resection more than 5 years ago is deemed to have a de-novo liver tumour and therefore can be included.

10. Adequate bone-marrow reserve, renal function and hepatic function as assessed by standard laboratory criteria:

    Absolute neutrophil count ≥ 1.0 x 10^9/L
    Platelet count ≥ 50 x 10^9/L
    Haemoglobin ≥ 9.0 g/dL
    INR ≤ 2.0 or Prothrombin time not more than 3 seconds above control
    Serum creatinine ≤ 1.5 times the Upper Limit of Normal (ULN)
    Albumin ≥ 2.5 g/dL
    Total bilirubin ≤ 1.5 times the ULN
    Alanine transaminase (ALT) ≤ 2.5 times the ULN
    Aspartate Transaminase (AST) ≤ 2.5 times the ULN

Exclusion Criteria

1. Patients unable to give inform consent.
2. Single lesion < 2 cm at the time of pre-op imaging.
3. The patient has previous or concomitant malignancies at other sites, except effectively treated non-melanoma skin cancers or carcinoma in situ of the cervix or effectively treated malignancy that has been in remission for over 5 years and highly likely to have been cured.
4. Encephalopathy
5. The patient has received a major organ allograft.
6. The patient is known to be positive for the Human Immunodeficiency Virus (HIV).
7. The patient has an uncontrolled bleeding disorder.

8. The patient has uncontrolled congestive heart failure or hypertension, unstable heart disease (coronary artery disease or myocardial infarction) or uncontrolled arrhythmia at the time of enrolment.

9. The patient has psychiatric or addictive disorders that may compromise his/her ability to give informed consent, or to comply with the study procedures.

10. The patient has other concurrent severe medical problems, unrelated to the malignancy, that would significantly limit full compliance with the study or expose the patient to unacceptable risk.

11. The patient has received any investigational or non-registered medicinal product (drug or vaccine) within the 30 days preceding the date of enrolment, or plans to receive such a drug during the study period.

12. For female patients: the patient is pregnant or lactating.

Supplementary Note 2: the genomic landscape of the patient cohort

The basic genomic landscape of HCC including the mutation burden, driver landscape, mutational signatures as well as copy number profiles have been described in several previous studies. In here, we focus on the strength of the multi-sectoring approach and present in detail what we have discovered in this cohort:

1) Tumor mutation burden and mutational signatures

By comparing the genomes of tumor sectors against the adjacent normal, we detected somatic mutations across multiple sectors of the same tumor. For all 67 patients, tumor mutation burden (TMB) varied greatly ranging from 0.5 to 16.3 mutations/Mb (median: 3.967 mutations/Mb) (Figure 1b, Supplementary Fig. 2a). To dissect the mutational process in the history of tumorigenesis for HCC, BayesNMF was used [6] and 13 known signatures from the extended COSMIC signatures [8] were found (Table S1c, Supplementary Fig. 5a) [7]. While classical age-related signatures SBS1 and SBS5 accounted for a high proportion of the somatic single nucleotide

variations (SNVs) (mean per sample contribution: 19% and 32%), a new age-related signature SBS40 was also present in high frequency in this cohort (7%). In addition, signature SBS4 (smoking, 8%), SBS6 (DNS-mismatch, 1%), SBS12 (unknown etiology, 6%), SBS22 (aristolochic acid (AA) exposure, 8%) and SBS24 (aflatoxin B1 exposure, 9%) were also detected in our cohort [58]. Of note, the proportion of AA signatures (SBS22) strongly correlated with the total mutation burden in both our cohort (R^2=0.54, p-value= 3.8×10^{-23}) and in the public TCGA cohort (R^2= 0.36, p−value= 1.0×10^{-6}) (Supplementary Fig. 5b). The other dominant signature, SBS24 (Aflatoxin signature, 9%) was known to be associated with the R249S mutation in tumor suppressor TP53 [59] and was also confirmed in our cohort (Supplementary Fig. 5c). Thus, a wide range of mutational processes existed in HCC, leading to high variability in TMB across tumors.

2) Common genetic drivers arise early, but rare ones arise late

In order to identify driver mutations in our patients, we compiled genomes from the four largest public HCC repositories (n=1,349 patients) and identified 62 driver genes using several statistical methods (see Methods, Supplementary Note 3). In total, 48 drivers were found in the current cohort. *TP53* (49%) and *CTNNB1* (31%) were the most frequently mutated drivers (Supplementary Fig.3), while known HCC drivers such as *ALB* (12%), *ARID1A* (10%) and *ARID2* (9%) were also found across several samples. In the noncoding regions, classical activating mutations G228A (24%) and G250A (5%) in the *TERT* promoter were also frequently found (Fig. 1b). Importantly, we also observed several novel HCC drivers in our cohort such as *ATRX* (4%) and *FRG1* (6%) (Supplementary Note 3). While most of the driver mutations were truncal events and tended to be shared across sectors (Fig. 1b), drivers that were less frequent at the population level across patients often occurred at the subclonal level within each patient (p-value=0.03, Fig. 1c). For instance, 79% of *TP53* mutations and 57% of *CTNNB1* mutations were truncal (Supplementary Fig.4), whereas the rare novel candidate *FRG1* was non-truncal in all the mutated cases (n=4), likely representing a late mutation event. This suggests that there are potentially many subclonal drivers in HCC, which might be missed by the conventional single sector approach, but could be discovered by the multi-regional sampling. In addition to this temporal sequence of driver alterations, we compared mutational signatures across the history of the tumorigenesis. Signatures related to external stimulus such as aristolochic acid (SBS22), smoking (SBS4) and aflatoxin B1 (SBS24) were more frequent in the early (truncal) part of the evolution (Fig.1d), implying their

active roles in tumor initiation (Supplementary Fig. 5d). In summary, even though classical driver mutations arise early in the history of tumorigenesis, there are significant amount of subclonal driver mutations across many HCCs, empowering subsequent clonal expansion and evolution.

3) Large chromosomal alterations appear early and active focal driver CNVs are frequent

Using the genome wide sequencing coverage as well as the frequency of germline variants, we inferred copy number alterations (CNA) and purity of our samples [18]. The most frequent chromosomal arm-level events included amplifications at 1q (68%), 8q (64%) and 7p (41%) and deletions at 8p (80%), 17p (64%) and 16q (64%) (Supplementary Fig. 6a, Supplementary Table 1d). Importantly, these arm-level amplification and deletion patterns were very similar across multiple sectors of the same patient and were also concordant with the TCGA cohort (Supplementary Fig. 6a). This suggests that large chromosomal alterations were likely early events in the history of tumorigenesis [60]. In addition to large scale copy number changes, we also performed driver CNA identification using the TCGA cohort (GISTIC analysis and Table S1e). Several focal CNAs containing cancer related genes such as *TERT* and *MYC* were recurrently amplified, while tumor suppressor genes such as *RB1* and *CASP3* were frequently deleted (Supplementary Fig. 6b, Supplementary Table 1e). Even though high frequency CNAs across patients also tend to be truncal events within individual patient (Figure 1c), there are many focal CNAs that are subclonal in many patients, Taken together, these observations suggest that while large-scale copy number events are often shared across sectors, there are active gain and loss of focal CNAs driving further diversification of each tumor.

Supplementary Note 3: integrative analysis of HCC drivers

In order to increase the power of detecting rare drivers in HCC, somatic mutations from four large HCC cohorts were collected. These include International Cancer Genome Consortium (ICGC) database (https://dcc.icgc.org/) which contains the Japanese cohort (Riken and National Cancer Center of Japan, n=514) and the French cohort (n=242). Mutation data for Japanese and French cohorts were downloaded from ICGC website (data release 18) (https://dcc.icgc.org/releases). For the TCGA (n=373) and Korean (n=231) cohorts, somatic mutation data were collected from the Firebrowse website (http://firebrowse.org/) and original publication respectively [13]. Somatic

mutations were first annotated using Oncotator (Version 1.9.2) [14]. We combined WES and WGS datasets by restricting our analysis to the coding regions of the genome.

Three different methods: MutSigCV (version 1.41) (a method based on mutation rate) [15], 20/20+ (a machine learning approach unifying multiple features of somatic mutations) [17] and TUSON Explorer (a method based on clustering of functionally important mutations) [16] were used to identify drivers in this combined cohort, targeting different aspects of the driver profile. Significantly mutated genes were identified based on FDR controlled q-values (q=0.1). Genes that were mutated in less than 1% of samples were further filtered and a final list of 62 driver genes were found combining all three methods. Among 62 drivers, 48 genes were found to be mutated in out cohort.

Supplementary Note 4: clonal deconvolution of patient tumors

Using patient ITH 52 as an example, we found that the branching pattern of evolutionary relationship clearly matched physical locations of tumor sectors (Fig. 2d). Other than the phylogenetic reconstruction, an important population genetic statistic which measure the genetic differentiation between samples is the Fixation index (i.e. FST)[22]. By comparing pairs of samples with their physical distance, we observed a clear isolation-by-distance (IBD) pattern where physically proximal sectors were also genetically more similar (Fig. 2e, p-value=$1.2e^{-120}$). At the sample level, both phylogenetic inference and population genetic analyses supported a clear spatial organization of genetic lineages in this patient.

In addition to sample level analysis (phylogeny or population genetic analysis), clonal deconvolution of tumor samples also provided further evidence on tumor spatial heterogeneity [61]. Using PhyloWGS [24] and Pyclone [23], we partitioned the genetic variation into discrete clones (Methods, Fig. 2g and Supplementary Fig. 7). For example, in patient ITH_52, 11 different clones were inferred using PhyloWGS and the clonal composition also supported the sample relationship (Fig. 2g). Using the clonal composition of patient tumors, we calculated the cosine distance between samples at the clonal level and also observed a clear linear relationship between the physical distance of the sectors and their clonal composition (Figure 2f), matching previous phylogenetic and population genetic analyses. Interestingly, even though the clonal inference

shows a different clonal composition using Pyclone, the spatial organization of genetic lineages stayed consistent (Supplementary Fig. 7). Across all 39 patients, the regression slopes between FST and physical distance took a wide range of values, of which the vast majority are statistically significant (34 out of 39 patients). In summary, using multiple approaches including phylogenetic reconstruction, population genetic analysis, and clonal deconvolution, we observed a clear IBD pattern in majority of HCC, which is very different from the pattern observed in colorectal tumors.

Supplementary Note 5: the prognostic value of the ITH features from the multi-sectoring approach

One key question in a multi-sectoring study is: whether ITH information gathered from the multi-sectoring approach can provide statistically significant information about patient survival. We employed two different approaches in addressing this question:

1) We compared the performance of the survival (Cox) models between a simplified model without the ITH features (model A, Supplementary Fig. 22a) and the full model with ITH features (Fig. 6b). Using Harrell's concordance index (c-index), we observed substantial decrease in prediction power for model A (c-index 0.87 vs 0.83; paired t-test p-value 7×10^{-4} , Supplementary Fig. 22c) [62]. Comparing the goodness of fit of the two models, we also observed lower performance for the model A than the full model using Akaike information criterion (AIC, 145.77 vs 141.30, Supplementary Fig. 22d) or using the likelihood-ratio test (Chi square test with p-value = 0.015).

2) An alternative approach is to test the full model against a model where we collect ITH information only from one of the sectors (model B, Supplementary Fig. 22b). For the DNA ITH in this scenario, we used Shannon index calculated from the clonal deconvolution results (from Pyclone) of the selected single sector (Methods). When we compare model B with the full model, we again observed substantial decrease in prediction power (c-index, 0.87 vs 0.77; p-value, 4.7×10^{-6}, Supplementary Fig. 22c). Comparing the goodness of fit, we also observed much lower performance for model B than the full model using AIC (157.23 vs 141.3, Supplementary Fig. 22d).

In summary, ITH information collected from the multi-sectoring approach provides significant information for patient prognosis and survival.

Supplementary Note 6: Modeling gene expression using the Ornstein-Uhlenbeck (OU) process

In order to understand how gene expression changes can drive the coexistence of multiple subtypes within a single tumor, we employed the classical stochastic process known as the Ornstein-Uhlenbeck (OU) process to model gene expression changes between the adjacent normal and multiple tumor sectors. Specifically, we asked the question whether a simpler model with two optima (i.e. the adjacent normal sample and a single RNA subtype from tumor, Supplementary Fig. 23a, Methods) or the more complex model with three optima (i.e. the adjacent normal and multiple tumor RNA subtypes) could fit the data better. We found that there are indeed more genes supporting the three optima model in patients with mixed subtypes (Supplementary Fig. 23, Supplementary Table 6, p-value=0.00011). In summary, different sectors could evolve to different subtypes and tumors with mixed-subtypes likely represented the transitory stage during disease progression where multiple RNA subtypes co-exist in the tumor before the more aggressive phenotypes (i.e. C2 and C3) become dominant in the population.

In addition to the statistical modeling, there is a coupling problem what are the factors that could lead to the transcriptomic changes. During disease progression, multiple factors including changes in the tumor cells (e.g. CNAs) or changes in the tumor microenvironment can drive the phenotypic evolution in the tumor population. Teasing apart these factors will require additional analysis such as novel deconvolutional methods looking at multiple layers of the tumor population or single cell sequencing, worth pursuing in a future study.

Supplementary Note 7: Spatial mixing in different tumor types

In the “big bang” model[63], the authors found that early subclonal (i.e. non-dominant) private alterations were pervasive within the tumor and can be found in distant regions, thus appearing variegated in the tumor. This variegation pattern is only found in the carcinoma samples, but not in adenoma. The authors hypothesized that this variegation pattern is due to aberrant sub-clone

mixing in the primordial tumor, followed by scattering during expansion and spatial mixing can be indicative of tumor progression (Figure 1 in the "big bang" work). However, in the study of HCC, we found a clear spatial segregation of genetic lineages within the tumor and tumor populations follows an isolation-by-distance pattern where physically closer sectors are genetically more similar. Through computational simulations[1], we found that a wide range of processes where tumors grow and expand outwards could be compatible with the data and might not require spatial mixing. This raised an interesting question whether the difference in spatial mixing can be due to cancer type specific evolution or whether these observations can still be compatible.

In the big bang study, the authors were able to sample a single gland and perform sequencing of highly clonal tissues. However, in our sampling strategy, we took multiple local cell populations which might be rather heterogeneous. In other words, these two sampling strategies can be interrogating tumor heterogeneity at different scales due to their sampling schema. At this point, we think the observations in CRC and HCC can still be compatible with each other because local spatial mixing can leave a clear trace when we sample locally clonal tissues, but not detectable when we sample macro-level populations.

Supplemental table titles

Table S1a: Clinical information of the cohort.

Table S1b: Sequencing coverage of the cohort

Table S1c: Signature proportions of the cohort

Table S1d: Arm level CNV changes across samples

Table S1e: Focal level CNV changes across samples

Table S2a: DNA ITH across the patients

Table S2b: Spatial mixing pattern across the patients

Table S2c: Neutrality test results across the samples

Table S3a: RNA subtypes of the samples

Table S3b: RNA ITH of the patients

Table S4a: Immune subtypes of the tumor samples

Table S4b: Immune ITH values of the patients

Table S4c: Truncal status of the targetable mutations

Table S5a: Correlation between clinical, molecular and ITH features

Table S5b: Univariate Cox model

Table S6: Proportion of genes supporting OU modeling with 2 or 3 optima

Supplementary References

1 Zhai, W. *et al.* The spatial organization of intra-tumour heterogeneity and evolutionary trajectories of metastases in hepatocellular carcinoma. *Nature Communications* **8**, 4565 (2017).

2 Li, H. & Durbin, R. Fast and accurate long-read alignment with Burrows–Wheeler transform. *Bioinformatics* **26**, 589-595 (2010).

3 Tarasov, A., Vilella, A. J., Cuppen, E., Nijman, I. J. & Prins, P. Sambamba: fast processing of NGS alignment formats. *Bioinformatics* **31**, 2032-2034 (2015).

4 Cibulskis, K. *et al.* Sensitive detection of somatic point mutations in impure and heterogeneous cancer samples. *Nature Biotechnology* **31**, 213 (2013).

5 Saunders, C. T. *et al.* Strelka: accurate somatic small-variant calling from sequenced tumor–normal sample pairs. *Bioinformatics* **28**, 1811-1817 (2012).

6 Kasar, S. *et al.* Whole-genome sequencing reveals activation-induced cytidine deaminase signatures during indolent chronic lymphocytic leukaemia evolution. *Nature Communications* **6**, 8866 (2015).

7 Rosenthal, R., McGranahan, N., Herrero, J., Taylor, B. S. & Swanton, C. deconstructSigs: delineating mutational processes in single tumors distinguishes DNA repair deficiencies and patterns of carcinoma evolution. *Genome Biology* **17** (2016).

8 Alexandrov, L. B. *et al.* The Repertoire of Mutational Signatures in Human Cancer. *bioRxiv*, 322859 (2018).

9 Alexandrov, L. B. *et al.* Signatures of mutational processes in human cancer. *Nature* **500**, 415-+ (2013).

10 Zhang, J. *et al.* International Cancer Genome Consortium Data Portal--a one-stop shop for cancer genomics data. *Database (Oxford)* **2011**, bar026 (2011).

11 Schulze, K. *et al.* Exome sequencing of hepatocellular carcinomas identifies new mutational signatures and potential therapeutic targets. *Nat Genet* **47**, 505-511 (2015).

12 Ally, A. *et al.* Comprehensive and Integrative Genomic Characterization of Hepatocellular Carcinoma. *Cell* **169**, 1327-1341.e1323 (2017).

13 Ahn, S.-M. *et al.* Genomic portrait of resectable hepatocellular carcinomas: Implications of RB1 and FGF19 aberrations for patient stratification. *Hepatology* **60**, 1972--1982 (2014).

14 Ramos, A. H. *et al.* Oncotator: Cancer Variant Annotation Tool. *Human Mutation* **36**, E2423-E2429 (2015).

15 Lawrence, M. S. *et al.* Mutational heterogeneity in cancer and the search for new cancer-associated genes. *Nature* **499**, 214 (2013).

16 Davoli, T. *et al.* Cumulative Haploinsufficiency and Triplosensitivity Drive Aneuploidy Patterns and Shape the Cancer Genome. *Cell* **155**, 948-962 (2013).

17 Tokheim, C. J., Papadopoulos, N., Kinzler, K. W., Vogelstein, B. & Karchin, R. Evaluating the evaluation of cancer driver genes. *Proc Natl Acad Sci U S A* **113**, 14330-14335 (2016).

18 Favero, F. *et al.* Sequenza: allele-specific copy number and mutation profiles from tumor sequencing data. *Annals of Oncology* **26**, 64-70 (2015).

19 Mermel, C. H. *et al.* GISTIC2.0 facilitates sensitive and confident localization of the targets of focal somatic copy-number alteration in human cancers. *Genome Biology* **12**, R41 (2011).

20 Shibata, T. & Aburatani, H. Exploration of liver cancer genomes. **11**, 340 (2014).

21 Saitou, N. & Nei, M. The neighbor-joining method: a new method for reconstructing phylogenetic trees. *Mol Biol Evol* **4**, 406-425 (1987).

22 Weir, B. S. & Cockerham, C. C. Estimating F-Statistics for the Analysis of Population Structure. *Evolution* **38**, 1358-1370 (1984).

23 Roth, A. *et al.* PyClone: statistical inference of clonal population structure in cancer. *Nature Methods* **11**, 396 (2014).

24 Deshwar, A. G. *et al.* PhyloWGS: Reconstructing subclonal composition and evolution from whole-genome sequencing of tumors. *Genome Biology* **16** (2015).

25 Ha, G. *et al.* TITAN: inference of copy number architectures, in clonal cell populations from tumor whole-genome sequence data. *Genome Research* **24**, 1881-1893 (2014).

26 Malikic, S., McPherson, A. W., Donmez, N. & Sahinalp, C. S. Clonality inference in multiple tumor samples using phylogeny. *Bioinformatics (Oxford, England)* **31**, 1349-1356 (2015).

27 McGranahan, N. *et al.* Clonal status of actionable driver events and the timing of mutational processes in cancer evolution. *Science Translational Medicine* **7**, 283ra254 (2015).

28 Williams, M. J., Werner, B., Barnes, C. P., Graham, T. A. & Sottoriva, A. Identification of neutral tumor evolution across cancer types. *Nature Genetics* **48**, 238 (2016).

29 Dobin, A. *et al.* STAR: ultrafast universal RNA-seq aligner. *Bioinformatics* **29**, 15-21 (2012).

30 Li, B. & Dewey, C. N. RSEM: accurate transcript quantification from RNA-Seq data with or without a reference genome. *BMC Bioinformatics* **12**, 323 (2011).

31 Love, M. I., Huber, W. & Anders, S. Moderated estimation of fold change and dispersion for RNA-seq data with DESeq2. *Genome Biology* **15**, 550 (2014).

32 Lee, D. D. & Seung, H. S. in *Advances in Neural Information Processing Systems 13* (eds T. K. Leen, T. G. Dietterich, & V. Tresp) 556-562 (MIT Press, 2001).

33 Hoshida, Y., Brunet, J.-P., Tamayo, P., Golub, T. R. & Mesirov, J. P. Subclass Mapping: Identifying Common Subtypes in Independent Disease Data Sets. *PLoS One* **2** (2007).

34 Hanzelmann, S., Castelo, R. & Guinney, J. GSVA: gene set variation analysis for microarray and RNA-seq data. *BMC Bioinformatics* **14**, 7 (2013).

35 Liberzon, A. *et al.* The Molecular Signatures Database Hallmark Gene Set Collection. *Cell Systems* **1**, 417-425 (2015).

36 Croft, D. *et al.* Reactome: a database of reactions, pathways and biological processes. *Nucleic Acids Res* **39**, D691-D697 (2011).

37 Hoshida, Y. *et al.* Integrative Transcriptome Analysis Reveals Common Molecular Subclasses of Human Hepatocellular Carcinoma. *Cancer research* **69**, 7385-7392 (2009).

38 Lee, J. S. *et al.* Classification and prediction of survival in hepatocellular carcinoma by gene expression profiling. *Hepatology* **40**, 667-676 (2004).

39 Yamashita, T. *et al.* EpCAM and α-Fetoprotein Expression Defines Novel Prognostic Subtypes of Hepatocellular Carcinoma. *Cancer Research* **68**, 1451 (2008).

40 Carbon, S. *et al.* AmiGO: online access to ontology and annotation data. *Bioinformatics* **25**, 288-289 (2009).

41 Daniel Nicorici, M. Ş., Henrik Edgren, Sara Kangaspeska, Astrid Murumägi, Olli Kallioniemi, Sami Virtanen, Olavi Kilkku. FusionCatcher – a tool for finding somatic fusion genes in paired-end RNA-sequencing data. *bioRxiv* (2014).
42 Cressler, C. E., Butler, M. A. & King, A. A. Detecting Adaptive Evolution in Phylogenetic Comparative Analysis Using the Ornstein–Uhlenbeck Model. *Syst Biol* **64**, 953-968 (2015).
43 Danaher, P. *et al.* Gene expression markers of Tumor Infiltrating Leukocytes. *Journal for ImmunoTherapy of Cancer* **5**, 18 (2017).
44 Ayers, M. *et al.* IFN-γ–related mRNA profile predicts clinical response to PD-1 blockade. *The Journal of Clinical Investigation* **127**, 2930-2940 (2017).
45 Jiang, P. *et al.* Signatures of T cell dysfunction and exclusion predict cancer immunotherapy response. *Nature Medicine* **24**, 1550-1558 (2018).
46 Garnelo, M. *et al.* Interaction between tumour-infiltrating B cells and T cells controls the progression of hepatocellular carcinoma. *Gut* **66**, 342-351 (2017).
47 Tennakoon, C. & Sung, W. K. BATVI: Fast, sensitive and accurate detection of virus integrations. *BMC Bioinformatics* **18**, 71 (2017).
48 Ding, Z. *et al.* Estimating telomere length from whole genome sequence data. *Nucleic Acids Res* **42**, e75 (2014).
49 Tamborero, D. *et al.* Cancer Genome Interpreter annotates the biological and clinical relevance of tumor alterations. *Genome Med* **10**, 25 (2018).
50 Chakravarty, D. *et al.* OncoKB: A Precision Oncology Knowledge Base. *JCO Precis Oncol* **2017** (2017).
51 Adnane, L., Trail, P. A., Taylor, I. & Wilhelm, S. M. Sorafenib (BAY 43-9006, Nexavar), a dual-action inhibitor that targets RAF/MEK/ERK pathway in tumor cells and tyrosine kinases VEGFR/PDGFR in tumor vasculature. *Methods Enzymol* **407**, 597-612 (2006).
52 Liu, L. *et al.* Sorafenib blocks the RAF/MEK/ERK pathway, inhibits tumor angiogenesis, and induces tumor cell apoptosis in hepatocellular carcinoma model PLC/PRF/5. *Cancer Res* **66**, 11851-11858 (2006).
53 Tohyama, O. *et al.* Antitumor activity of lenvatinib (e7080): an angiogenesis inhibitor that targets multiple receptor tyrosine kinases in preclinical human thyroid cancer models. *J Thyroid Res* **2014**, 638747 (2014).
54 Boige, V. *et al.* Efficacy, safety, and biomarkers of single-agent bevacizumab therapy in patients with advanced hepatocellular carcinoma. *Oncologist* **17**, 1063-1072 (2012).
55 Siegel, A. B. *et al.* Phase II trial evaluating the clinical and biologic effects of bevacizumab in unresectable hepatocellular carcinoma. *J Clin Oncol* **26**, 2992-2998 (2008).
56 El-Khoueiry, A. B. *et al.* Nivolumab in patients with advanced hepatocellular carcinoma (CheckMate 040): an open-label, non-comparative, phase 1/2 dose escalation and expansion trial. *Lancet* **389**, 2492-2502 (2017).
57 Finn, R. S. *et al.* Pembrolizumab As Second-Line Therapy in Patients With Advanced Hepatocellular Carcinoma in KEYNOTE-240: A Randomized, Double-Blind, Phase III Trial. *J Clin Oncol* **38**, 193-202 (2020).
58 Letouzé, E. *et al.* Mutational signatures reveal the dynamic interplay of risk factors and cellular processes during liver tumorigenesis. *Nature Communications* **8**, 1315 (2017).
59 Zhang, W. *et al.* Genetic Features of Aflatoxin-associated Hepatocellular Carcinomas. *Gastroenterology* (2017).
60 Gao, R. *et al.* Punctuated copy number evolution and clonal stasis in triple-negative breast cancer. *Nat Genet* **48**, 1119-1130 (2016).
61 Greaves, M. & Maley, C. C. Clonal evolution in cancer. *Nature* **481**, 306-313 (2012).

62 Haibe-Kains, B., Desmedt, C., Sotiriou, C. & Bontempi, G. A comparative study of survival models for breast cancer prognostication based on microarray data: does a single gene beat them all? *Bioinformatics* **24**, 2200-2208 (2008).

63 Sottoriva, A. *et al.* A Big Bang model of human colorectal tumor growth. *Nature Genetics* **47**, 209-+ (2015).
